# Genome sequencing reveals *CCDC88A* variants in malformations of cortical development and immune dysfunction

**DOI:** 10.1093/hmg/ddaf081

**Published:** 2025-05-22

**Authors:** Johanna Lehtonen, Anna H Hakonen, Antti Hassinen, Sanne Iversen Lurås, Meri Kaustio, Virpi Glumoff, Francisca Hinrichsen, Weiwei Li, Anna-Maija Sulonen, Sanna Wickman, Henrikki Almusa, Minttu Polso, Maarit Palomäki, Sirpa Kivirikko, Kristiina Avela, Kaarina Heiskanen, Vilja Pietiäinen, Kristiina Aittomäki, Janna Saarela

**Affiliations:** Centre for Molecular Medicine Norway (NCMM), University of Oslo, Oslo Science Park, Gaustadalléen 2, Oslo 0349, Norway; Institute for Molecular Medicine Finland (FIMM), HiLIFE, University of Helsinki, Biomedicum 2, Tukholmankatu 8, Helsinki 00290, Finland; Folkhälsan Research Center, Biomedicum 1, Haartmaninkatu 8, Helsinki 00290, Finland; Department of Medical Genetics, Oslo University Hospital, Building 25, Kirkeveien 166 (Ullevål), Oslo 0450, Norway; Department of Clinical Genetics, HUSLAB, HUS Diagnostic Center, Helsinki University Hospital and University of Helsinki, Topeliuksenkatu 32, Helsinki 00290, Finland; Institute for Molecular Medicine Finland (FIMM), HiLIFE, University of Helsinki, Biomedicum 2, Tukholmankatu 8, Helsinki 00290, Finland; Centre for Molecular Medicine Norway (NCMM), University of Oslo, Oslo Science Park, Gaustadalléen 2, Oslo 0349, Norway; Institute for Molecular Medicine Finland (FIMM), HiLIFE, University of Helsinki, Biomedicum 2, Tukholmankatu 8, Helsinki 00290, Finland; Medical Research Laboratory Unit, Faculty of Medicine, University of Oulu, Pentti Kaiteran katu 1, Oulu 90570, Finland; Centre for Molecular Medicine Norway (NCMM), University of Oslo, Oslo Science Park, Gaustadalléen 2, Oslo 0349, Norway; Centre for Molecular Medicine Norway (NCMM), University of Oslo, Oslo Science Park, Gaustadalléen 2, Oslo 0349, Norway; Institute for Molecular Medicine Finland (FIMM), HiLIFE, University of Helsinki, Biomedicum 2, Tukholmankatu 8, Helsinki 00290, Finland; Department of Pediatric Neurology, Hyvinkää Hospital, Helsinki and Uusimaa Hospital District, Sairaalankatu 1, Hyvinkää 05850, Finland; Institute for Molecular Medicine Finland (FIMM), HiLIFE, University of Helsinki, Biomedicum 2, Tukholmankatu 8, Helsinki 00290, Finland; Institute for Molecular Medicine Finland (FIMM), HiLIFE, University of Helsinki, Biomedicum 2, Tukholmankatu 8, Helsinki 00290, Finland; Department of Radiology, Helsinki University Hospital, Stenbäckinkatu 9, Helsinki 00290, Finland; Department of Clinical Genetics, HUSLAB, HUS Diagnostic Center, Helsinki University Hospital and University of Helsinki, Topeliuksenkatu 32, Helsinki 00290, Finland; Department of Clinical Genetics, HUSLAB, HUS Diagnostic Center, Helsinki University Hospital and University of Helsinki, Topeliuksenkatu 32, Helsinki 00290, Finland; Turku University Hospital, University of Turku, Kiinamyllynkatu 4-8, Turku 20520, Finland; New Children’s Hospital, HUS, Helsinki University Hospital and University of Helsinki, Stenbäckinkatu 9, Helsinki 00290, Finland; Institute for Molecular Medicine Finland (FIMM), HiLIFE, University of Helsinki, Biomedicum 2, Tukholmankatu 8, Helsinki 00290, Finland; Department of Medical and Clinical Genetics, University of Helsinki, Fabianinkatu 33, Helsinki 00100, Finland; Centre for Molecular Medicine Norway (NCMM), University of Oslo, Oslo Science Park, Gaustadalléen 2, Oslo 0349, Norway; Institute for Molecular Medicine Finland (FIMM), HiLIFE, University of Helsinki, Biomedicum 2, Tukholmankatu 8, Helsinki 00290, Finland; Department of Medical Genetics, Oslo University Hospital, Building 25, Kirkeveien 166 (Ullevål), Oslo 0450, Norway

**Keywords:** CCDC88A, girdin, malformations of cortical development, immunodeficiency, actinopathy

## Abstract

Malformations of cortical development (MCDs) encompass a diverse group of genetic and clinical disorders. Here, we aimed to determine a genetic etiology for two siblings manifesting MCD, microcephaly, epilepsy, intellectual disability, and susceptibility to infections. A missense variant (NM_018084:c.929A > C, p.Asp310Ala) and an intragenic deletion (exons 14–16) in *CCDC88A* were identified as compound heterozygous in patients by genome sequencing. Truncating homozygous *CCDC88A* variants are known to cause an ultra-rare syndrome manifesting with MCD, microcephaly, seizures, and severe neurological impairment. *CCDC88A* encodes girdin, which is essential for various cell functions, such as actin remodeling and cell proliferation. Western blot analysis showed that the missense variant allele was expressed in fibroblasts at a level compatible with a heterozygous allele, whereas a truncated protein from the deletion allele was barely detectable. Proliferation and wound-healing assays revealed that girdin-deficient fibroblasts proliferated faster and migrated slower than controls. High-content imaging highlighted girdin-deficient fibroblasts as smaller and their actin remodeling disrupted, leading to perinuclear accumulation of endolysosomal organelles. To confirm these cellular phenotypes resulted from girdin loss, CRISPR-Cas9 edited knockout models of healthy fibroblasts were created, replicating the observations in patient cells. Additionally, the siblings exhibited reduced monocytoid and plasmacytoid dendritic cells, suggesting compromised immunity due to girdin deficiency. In summary, the study describes the first case of a *CCDC88A* missense variant and intragenic deletion associated with MCD. It demonstrates altered immunity and girdin-related cellular changes, such as cell morphology and proliferation-migration dichotomy, in patient and knockout fibroblasts, reinforcing the pathogenic relevance of these variants.

## Graphical Abstract

**Graphical Abstract:**
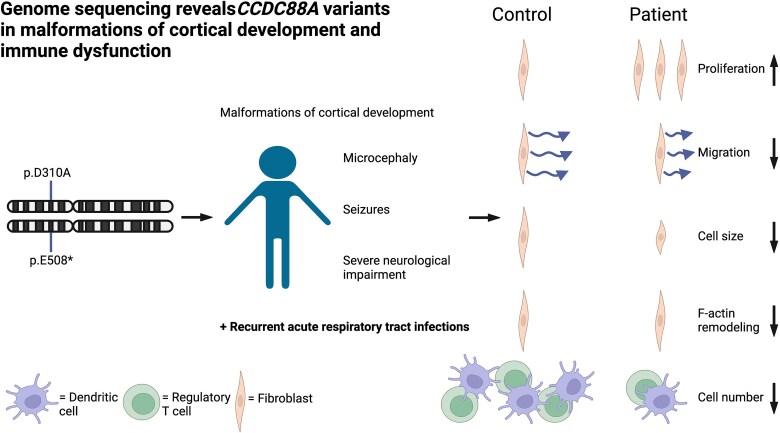

## Introduction

Malformations of cortical development (MCD) are genetically and clinically heterogeneous disorders typically manifesting with developmental delay, intellectual disability (ID), and severe epilepsy—leading to significant neurological morbidity. Whole genome sequencing (WGS) offers means for unveiling the underlying genetic defects in MCD, but variant interpretation is often difficult and may require additional functional studies.

*CCDC88A* encodes for girdin, ‘girders of actin filaments’, also known as Akt phosphorylation enhancer (APE), Gα-interacting vesicle-associated protein (GIV), and Hook-related protein 1 (HkRP1) [1–4]. Girdin is expressed ubiquitously in vertebrates [1, 3]. It is highly conserved throughout evolution although the C-terminus shows considerable homology only in vertebrates [5] which suggests that girdin’s ability to bind and activate guanine nucleotide-binding (G) proteins is vertebrate-specific [6]. The N-terminal domain of girdin has a Hook-like domain that can bind to microtubules [2] and dynamin 2 [7]. The central part of girdin is predicted to form a long coiled-coil domain that can homodimerize and oligomerize [1]. The C-terminal domain of girdin links cytoskeletal actin to the plasma membrane [1] and binds multiple other proteins, such as epidermal growth factor receptor (EGFR) [3, 4, 8–10]. Additionally, a long isoform of girdin contains a PDZ-binding motif, a highly abundant protein–protein interaction domain, that localizes to cell–cell junctions [6]. Girdin interacts with Disrupted-in-Schizophrenia 1 (DISC1), a protein that is involved in neurite outgrowth and cortical development through its interaction with other proteins, possibly through multiple domains [11, 12]. Altogether, girdin is primarily a cytoplasmic protein that can translocate to the plasma membrane, where it acts as a multi-modular scaffolding protein and orchestrates many cellular functions through its interactions.

In cells, girdin interacts with the PI3K/AKT/mTOR signaling pathway that responds to growth factors and regulates many vital cell functions such as migration, proliferation, apoptosis, cell survival, growth, and cellular metabolism [1, 4]. Additionally, girdin has an essential role in various cell functions such as cytoskeletal actin integrity and organization [1, 13–15], cell division [16], neuron maturation and development [11], macrophage chemotaxis [17], reduction of macrophage proinflammatory responses [18], immunological synapse formation [19], selective endocytosis [7], and regulation of autophagy [8, 20]. Yet, one of the main roles of girdin is to orchestrate the migration-proliferation dichotomy of cells [21]. The G protein α subunit (Gα) binding and activating (GBA) domain in the C-terminus of girdin functions as a guanine-nucleotide exchange factor (GEF) that can activate Gα activity-inhibiting polypeptide 1 (Gαi) [22], and a guanine nucleotide dissociation inhibitor (GDI) that can inhibit Gα group S (Gαs) [21]. Taken together, girdin has myriad roles in cells and it functions as a link between environmental signals and intracellular responses.

In mice, the expression of *CCDC88A* RNA is spatiotemporally regulated during embryogenesis [2], and in the developing nervous system, it is more expressed in differentiating than in proliferating neurons [12]. Studies of girdin-deficient mice have shown that in the developing brain, the function of girdin is more crucial in the postnatal rather than in the embryonic period [11, 23]. Girdin is critical for the dentate granule cell (DGC) migration and positioning at the postnatally formed dentate gyrus [11]. In response to growth factors, girdin also regulates neuroblast chain migration postnatally in the rostral migratory stream [23]. In contrast to DGC migration, chain migration of subventricular zone neuroblasts is independent of girdin-DISC1 interaction, suggesting that girdin regulates migration with differing mechanisms in different neuronal cell types [11, 23]. Furthermore, girdin-deficient mice manifested hypoplasia of the olfactory bulb in the brain [11, 23], suffered from postnatal growth retardation, and died prematurely by one month of age [24, 25].

Here we describe compound heterozygous *CCDC88A* variants identified by WGS in two siblings with MCD and a severe neurological phenotype. The *CCDC88A* variants include the first missense variant and the first intragenic deletion in *CCDC88A* underlying human encephalopathies. Previously, truncating homozygous *CCDC88A* variants have been reported in association with MCD, congenital microcephaly, seizures, profound cognitive delay, severe cortical visual inattention, and progressive brain atrophy in two consanguineous pedigrees [26, 27]. Two other families with truncating homozygous *CCDC88A* variants have been described with a neurological phenotype but with very little clinical information [28, 29]. We sought to further characterize this newly emerging disease and its pathogenetic mechanisms. Our findings suggest that girdin dysfunction may also lead to impaired function of the immune system and further infection susceptibility in patients. Additionally, we characterize the functional effects of girdin variants for the first time in primary patient fibroblasts and show aberrations in migration-proliferation dichotomy and cell organelle morphology, and repeat the most important findings with CRISPR-Cas9 knockout (KO) experiments.

## Results

### Study design

The study aimed to identify a molecular genetic diagnosis for the family with two siblings presenting with developmental delay, intellectual disability (ID), severe epilepsy, and infection susceptibility. Cellular consequences of the plausible candidate variants, identified in *CCDC88A,* were studied by Western blot, proliferation assay, migration assay, and high-content image-based cell phenotyping in patient’s fibroblasts. Although previous reports in patients with truncating variants in *CCDC88A* had not described immune defects in connection with the disease, our patients showed increased susceptibility to upper and lower respiratory infections. Thus, we set to further characterize their immune phenotype. Lastly, we silenced *CCDC88A* with CRISPR-Cas9 in the control fibroblasts and recapitulated with this CRISPR model the proliferation assay and part of the image-based cell phenotyping results that the patient fibroblasts manifested. The migration phenotype and cell size alterations were not studied with the KO model.

### A missense variant and a deletion were identified in *CCDC88A*

**Figure 1:**
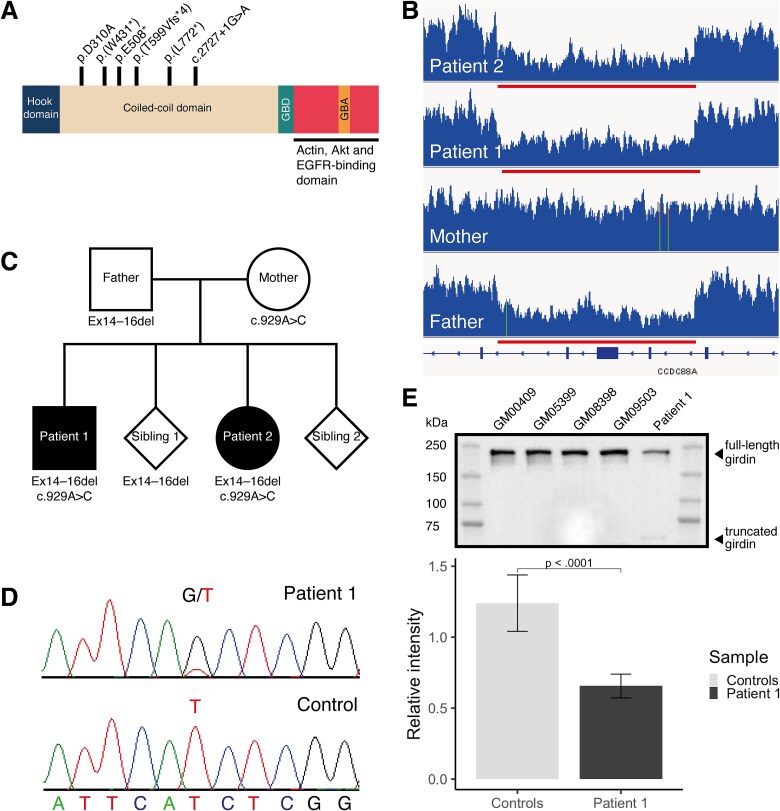
Both affected siblings were compound heterozygous for *CCDC88A* variants. (A) The figure illustrates the location of newly identified girdin variants p.D310A and p.E508* (ex14-16del truncates girdin), and the earlier identified variants p.(W431*), p.(T599Vfs*), p.(L772*), and c.2727 + 1G > a [26–29]. The N-terminus of girdin has a microtubule-binding hook-like domain [2]. The central part of girdin consists of a coiled-coil domain which can homodimerize and oligomerize [1]. The Gα-binding domain (GBD) includes the PI4P-binding domain that translocates girdin to the plasma membrane [1]. The Gα binding and activating (GBA) domain can function as a guanine-nucleotide exchange factor (GEF) activating Gαi [22] or a guanine nucleotide dissociation inhibitor (GDI) inhibiting Gαs [21]. The C-terminus of girdin binds to multiple proteins such as actin [1], Akt [4], and EGFR [10]. Only major interactions are annotated. (B) The integrative genomics viewer (IGV) visualizes the lower read depth in the deletion region. Exons 14–16 of *CCDC88A* are deleted in the paternally inherited allele in the patient samples. (C) The family pedigree illustrates the segregation of the intragenic deletion and the missense variant (c.929A > C:p.Asp310Ala) identified in *CCDC88A*. (D) RT-PCR analysis and sanger sequencing focusing on nucleotide c.929 indicated that very little truncated girdin is expressed in patient 1 fibroblasts from the paternal allele. The sequencing electropherograms are in the reverse strand. (E) A western blot image shows that full-length girdin (~220 kDa) and small amounts of truncated protein (~59 kDa) are present in the patient 1 fibroblasts. A barplot illustrates that the amount of full-length girdin with p.D310A missense variant in patient 1 fibroblasts is ~ 50% of the amount in controls. This corresponds to an expression level of a heterozygous allele. Data are shown as mean ± SD. Wilcoxon matched-pairs signed rank test was used.

Analysis of WGS data performed for the affected siblings and their parents identified a maternally inherited heterozygous missense variant and a paternally inherited intragenic deletion in *CCDC88A* encoding for girdin (Fig. 1A), in both patients. The *CCDC88A* variant NM_001135597.1:c.929A > C, p.Asp310Ala is located in the coiled-coil domain of girdin that can dimerize and oligomerize. The missense variant changes a highly conserved negatively charged aspartic acid (Asp) with a hydrophobic alanine (Ala). The *in-silico* algorithms AlphaMissense, REVEL, CADD phred, and GERP++ rejected substitutions scores were > 0.9609; 0.594; 29.6; and 5.26; respectively, supporting variant pathogenicity. This variant was present in Finns at a very low minor allele frequency (MAF) of 0.0000924 but absent in other populations in the Genome Aggregation Database (gnomAD, v2.1.1), and it was therefore compatible with a recessive mode of inheritance. The heterozygous deletion encompassing exons 14–16 of *CCDC88A* was observed both with genome-wide, read-depth-based copy number variant (CNV) analysis and by visualization with the Integrative Genomics Viewer (IGV; Fig. 1B) in both patients and their father. Breakpoints of the intragenic deletion were estimated with split-read analysis, and the deletion size (9796 bp) and location (NC_000002.11:g.55556194_55565990del) were confirmed using capillary sequencing (Supplementary Material, Fig. S1). Figure 1C presents segregation of the *CCDC88A* variants in the family. Other WGS results are included in the Supplementary Material.

### *CCDC88A*/Girdin expression was reduced in patient fibroblasts

Girdin RNA expression was investigated in Patient 1 (P1) fibroblasts using RT-PCR. Minimal expression of the wildtype allele c.929 T harboring the intragenic deletion was detected in a sample (Fig. 1D). This suggested that nonsense-mediated decay was incomplete because all the RNA including a premature stop codon was not degraded. This was verified by western blotting, which showed a faint truncated protein band of ~ 59 kDa in size (Fig. 1E and Supplementary Material, Fig. S2). The deletion truncates girdin at the coiled-coil domain. The quantification of the protein bands showed that the levels of full-length girdin in P1 fibroblasts were ~ 50% of the girdin protein levels in four controls’ fibroblasts.

### Girdin variants increase fibroblast proliferation

**Figure 2:**
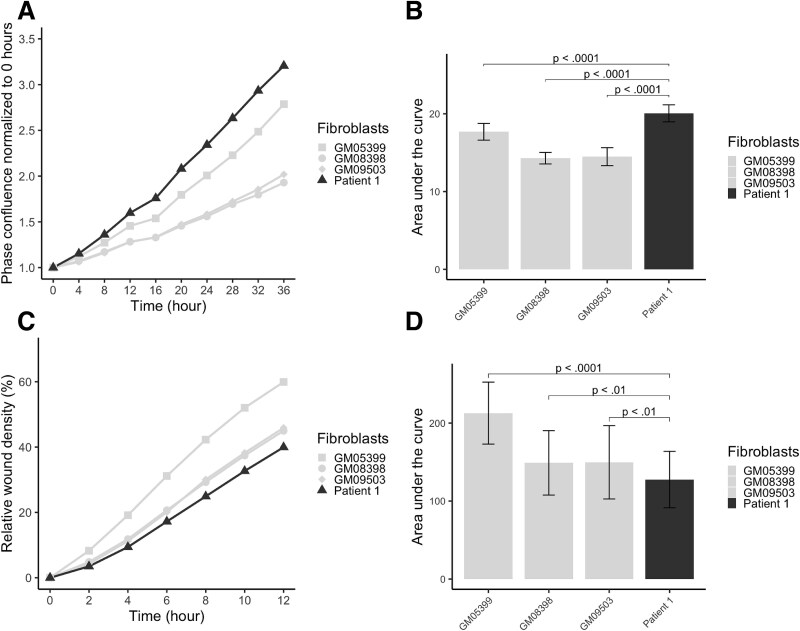
Proliferation and migration processes are altered in patient 1 primary fibroblasts. (A) The proliferation assay was based on the quantification of the change in cell confluency over time. Mean phase confluency normalized to analysis starting point and time are plotted. (B) Area under the curve (AUC) values were calculated for the proliferation assay replicates (*n* = 60 per primary fibroblasts). Patient 1 fibroblasts proliferated significantly more rapidly than the control fibroblasts. (C) The fibroblast migration over time was studied in a scratch wound-healing assay. The time and percentage of mean relative wound density (RWD) are plotted for the fibroblasts studied. (D) Area under the curve (AUC) values were calculated for the wound-healing assay replicates (*n* = 54 per primary fibroblasts) until a 12 h time point. A comparison of AUC values shows that patient 1 fibroblasts migrated slower compared with all the control fibroblasts. Data are shown as mean ± SD and an unpaired t-test was used.

Girdin-EGFR interaction inhibits Gαs at the endosomes, which further inhibits the Ras/Raf/MAPK/ERK pathway and cell proliferation [30]. The proliferation of P1 and control fibroblasts was examined using live cell imaging to study the possible effects of girdin variants on cell proliferation. The proliferation rates of different fibroblasts were compared. The proliferation activity of control cells suggested an association between cell proliferation and age: the fibroblasts from a one-year-old control (GM05399) proliferated significantly more than the fibroblasts from the eight (GM08398, *P* ≤ 0.0001) and ten-year-old (GM09503, *P* ≤ 0.0001) controls. However, the P1 fibroblasts, taken at the age of five years, proliferated even more rapidly than those from the youngest control (*P* ≤ 0.0001) (Fig. 2A and B).

### Girdin variants decrease fibroblast migration

PI3K-Akt-dependent cell migration requires phosphorylation of the C-terminal domain of girdin by Akt at the leading edge of the migrating cells [1, 24, 31]. Also, girdin autophosphorylates EGFR upon epidermal growth factor (EGF) stimulation, which leads to the activation of Gαi at the plasma membrane and enhancement of the PI3K-Akt-dependent cell migration [32]. The neuronal migration is, however, suggested to be dependent on girdin’s C-terminal basic amino acid residue-rich region and not on girdin phosphorylation by Akt, and therefore, the migration mechanism differs between neuronal and non-neuronal cells [23]. Considering the central role of girdin on cell migration, we examined how the studied variants affect cell migration of P1 fibroblasts. The cell migration was studied with a wound-healing assay utilizing live cell imaging while the fibroblast proliferation was blocked. Fibroblasts from the youngest control migrated significantly more than the fibroblasts from the school-aged controls GM08398 (*P* ≤ 0.0001) or GM09503 (*P* ≤ 0.0001). Still, P1 fibroblasts migrated slower than any of the control fibroblasts (Fig. 2C and D). The difference in migration velocity was most significant (*P* ≤ 0.0001) between P1 fibroblasts and the youngest control GM05399 (aged one year). A correlation between the migration velocity and age at fibroblast biopsy sampling has been shown previously [33]. Our findings demonstrate that the ratio between proliferation and migration was altered in P1 fibroblasts.

### Girdin variants reduced fibroblast size

**Figure 3:**
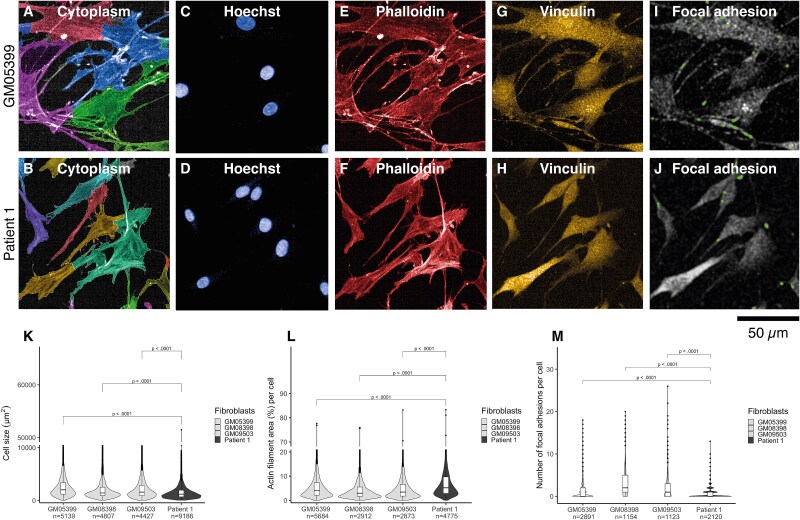
Patient 1 fibroblasts were smaller, had altered cytoskeletal actin remodeling, and a reduced focal adhesion number compared with control fibroblasts. An algorithm-based mask for the cytoplasm of (A) GM05399 and (B) Patient 1; cell nucleus staining of (C) GM05399 and (D) Patient 1; cytoskeletal actin staining of (E) GM05399 and (F) Patient 1; vinculin staining of (G) GM05399 and (H) Patient 1; an algorithm-based mask for focal adhesion structures that are vinculin-positive and colocalize with F-actin in (I) GM05399 control and (J) Patient 1; (K) a violin plot illustrates that patient 1 fibroblasts are smaller than controls; (L) a violin plot shows that the F-actin structures take a larger area in patient 1 fibroblast compared with controls. There is more variation in F-actin structures in patient 1 fibroblasts than controls (the violin has a longitudinal rather than latitudinal shape); (M) a violin plot reveals that the focal adhesion number was reduced in patient 1 fibroblasts. GM05399 was selected to represent controls in the images, data for cell size analysis were pooled from three different technical replicates, and for F-actin/focal adhesion analysis from one technical repeat, no EGF stimulation, unpaired t-test, and *n* = cell number.

We examined, with high-content (HC) confocal microscopy, whether the girdin variants display phenotypic effects in P1 fibroblasts. For imaging, the cells were stained for actin cytoskeleton and with different antibodies to detect cell organelles or proteins (Supplementary Material, Table S1). The single cells in the microscopic images were segmented for quantitative analysis of the phenotypic features (Supplementary Material, Table S2). First, the cell size was quantified using a segmentation mask from a combined image from both phalloidin and tubulin staining. Figure 3A–J shows exemplary images of immunofluorescence staining and algorithm-based masks. The examination of cell size showed that the P1 fibroblasts were significantly (*P* ≤ 0.0001) smaller in size than any of the control fibroblasts (mean cell size [μm^2^], ±SD: GM05399 2599 ± 2106, GM08398 2149 ± 2195, GM09503 2233 ± 2062, P1 1558 ± 1575; Fig. 3K). Second, the membrane and the cytoplasm sections of the total cell area were quantified as the outer 10% and inner 90% of the cell area respectively. Both the cytoplasm region (*P* ≤ 0.0001) and the membrane region (*P* ≤ 0.0001) of the total cell area were reduced in P1 fibroblasts compared with any of the controls (mean cytoplasm region [%] of total cell area, ±SD: GM05399 50.9 ± 10.3, GM08398 50.1 ± 11.5, GM09503 50.8 ± 12, P1 49.1 ± 12.4; mean membrane region [%] of total cell area: GM05399 39.7 ± 12.5, GM08398 36.9 ± 13.5, GM09503 36.6 ± 13.1, P1 33.4 ± 12.5; Supplementary Material, Fig. S3).

### Girdin variants affect cytoskeletal actin remodeling

As girdin has various effects on the cytoskeletal actin organization [1, 13–15], F-actin structures were studied in fibroblasts by phalloidin staining with HC confocal microscopy (Fig. 3E and F). The analysis of the features of phalloidin staining showed that the actin filament area was larger (*P* ≤ 0.001) and the actin filaments were both longer (*P* ≤ 0.0001) and wider (*P* ≤ 0.05) in P1 fibroblasts compared with any control fibroblasts (mean actin filament area [μm^2^], ±SD: GM05399 94 ± 58, GM08398 101 ± 55, GM09503 104 ± 61, P1 109 ± 68; mean actin filament length [μm], ±SD: GM05399 23.14 ± 8.93, GM08398 24.82 ± 8.94, GM09503 25.63 ± 10.48, P1 26.79 ± 10.86; mean actin filament width [μm], ±SD: GM05399 3.14 ± 0.58, GM08398 3.13 ± 0.46, GM09503 3.13 ± 0.5, P1 3.16 ± 0.58; Supplementary Material, Fig. S4). Further analysis of the width-to-length ratio of actin filaments confirmed that they were especially lengthened rather than widened (*P* ≤ 0.0001) in P1 fibroblasts in comparison with any control fibroblasts (mean width-to-length ratio, ±SD: GM05399 0.181 ± 0.033, GM08398 0.179 ± 0.028, GM09503 0.173 ± 0.03, P1 0.167 ± 0.035; Supplementary Material, Fig. S4). These results indicate that cytoskeletal actin remodeling is altered in P1 fibroblasts. These alterations in cytoskeletal actin remodeling also resulted in a larger actin filament area per cell (*P* ≤ 0.0001) in P1 fibroblasts than in any control fibroblasts (mean actin filament area [%] per cell, ±SD: GM05399 6.2 ± 7; GM08398 4.8 ± 6.1; GM09503 5.7 ± 7.4; P1 7.8 ± 8.3; Fig. 3L). These results suggest that in P1 fibroblasts, the actin network is sparse with few crosslinks to other actin molecules and that the actin filaments are thereby aggregated compared with control fibroblasts.

### Girdin variants reduce the number of focal adhesion structures

Vinculin is located in the focal adhesions—foci of girdin signaling and integrin-focal adhesion kinase (FAK) signaling via activation of G protein [34]. Because girdin is essential for focal adhesion integrity [34], the focal adhesion structures were investigated by vinculin immunostaining (Fig. 3G and H). Vinculin staining showed no systematic differences in vinculin spot size and intensity between P1 fibroblasts and control fibroblasts (mean vinculin spots [μm^2^], ±SD: GM05399 7.7 ± 4, GM08398 10.4 ± 6, GM09503 11 ± 8.2, P1 9.9 ± 10.5; mean vinculin intensity in focal adhesions, ±SD: GM05399 20 113 ± 13 675, GM08398 29 222 ± 22 383, GM09503 31 638 ± 35 839, P1 27 017 ± 49 239, Supplementary Material, Fig. S5). However, the vinculin area-to-number ratio was increased in P1 fibroblasts compared with controls (mean vinculin area-to-number ratio, ±SD: GM05399 4.4 ± 3.7, GM08398 4.6 ± 5.4, GM09503 5.6 ± 7.7, P1 7.2 ± 10.2, Supplementary Material, Fig. S5). This suggests a difference in focal adhesion number which was confirmed by segmenting the focal adhesion sites by vinculin staining and by quantifying colocalized sites of vinculin-positive focal adhesion sites within F-actin positive structures (Fig. 3I–J). The results show a reduced number of focal adhesions between P1 fibroblasts and controls (mean focal adhesion number per cell, ±SD: GM05399 1.2 ± 2.1; GM08398 3.2 ± 3.4; GM09503 2.5 ± 3.2; P1 0.7 ± 1.2; (Fig. 3M).

**Figure 4:**
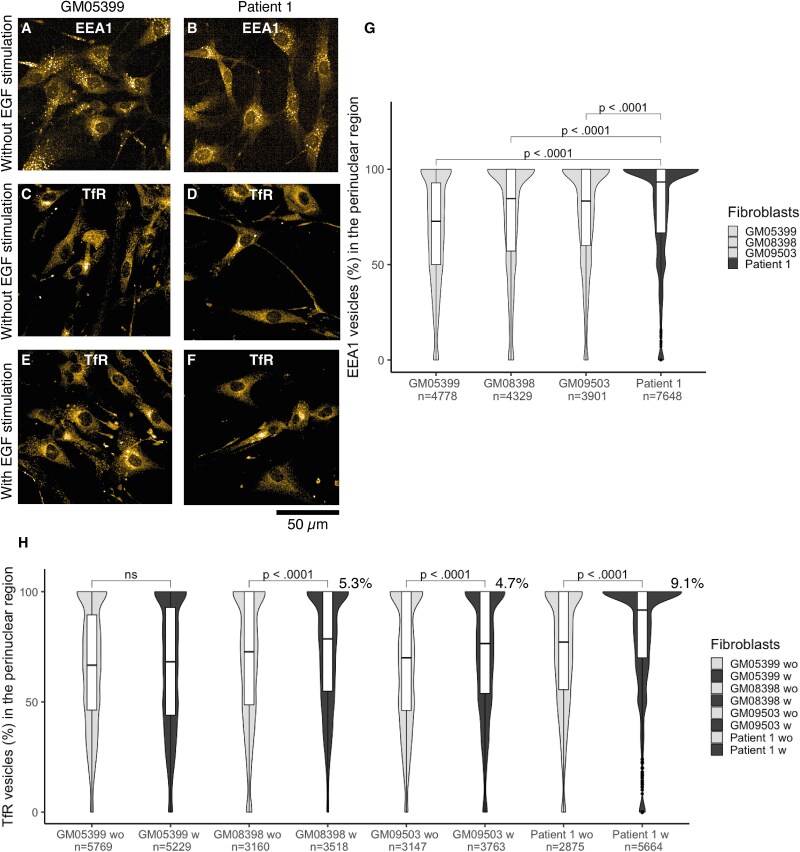
Girdin variants in patient 1 fibroblasts lead to perinuclear accumulation of early endosome (EEA1) and transferrin receptor (TfR). EEA1 staining without EGF stimulation in (A) GM05399 and (B) Patient 1; TfR staining without EGF stimulation in (C) GM05399 and (D) Patient 1; TfR staining with EGF stimulation in (E) GM05399 (F) and patient 1; (G) a violin plot shows that EEA1 vesicles accumulate significantly more in the perinuclear region of the patient 1 fibroblast than in controls; (H) a violin plot highlights how EGF stimulation enhances the TfR accumulation in the perinuclear region in all the fibroblasts except GM05399. The effect was strongest (9.1% increase) in the patient 1 fibroblasts. GM05399 was selected to represent controls in the images, data are pooled from three different technical replicates, *n* = cell number, unpaired t-test, wo = without and w = with epidermal growth factor stimulation.

### Girdin variants alter the location, number, and size of cell organelles of the endolysosomal pathway

Because girdin is known to regulate endosomal pathways and autophagosomes [7, 8, 20], these pathways were studied by immunofluorescence staining of P1 fibroblasts by HC confocal microscopy. Alterations in the endolysosomal pathway were observed in P1 fibroblasts (Fig. 4A–F). Early endosomes (EEA1), lysosome-associated membrane proteins I (LAMP1) and II (LAMP2), lysosomal glycoprotein III (LIMP), and a cargo, the transferrin receptor (TfR), were accumulated in the perinuclear region of P1 fibroblasts, and they were not spread throughout the cytoplasm as in control fibroblasts (Fig. 4G, and Supplementary Material, Table S3 and Fig. S6). Additionally, the number of EEA1, LAMP1, LAMP2, LIMP, and TfR-positive vesicles was reduced in P1 fibroblasts compared with controls (Supplementary Material, Table S3, Fig. S7, and S8). The EEA1, LAMP2, LIMP, and TfR-positive endolysosomal vesicles were smaller in P1 fibroblasts than in controls (Supplementary Material, Table S3 and Supplementary Material, Fig. S9). The LAMP1 vesicles were smaller in P1 fibroblasts except in comparison with the GM08398 control (Supplementary Material, Table S3 and Fig. S9).

Because girdin interacts with EGFR [10] and this interaction is known to affect i.e. proliferation-migration dichotomy of the cells [30, 32], EGF was added into the cell media to examine if this stimulation could enhance some of the phenotypes that the studied girdin variants caused in the P1 fibroblasts. Indeed, such an effect was observed, as EGF addition enhanced the TfR accumulation by 9.1% in the perinuclear region of P1 fibroblasts, whereas, in the control fibroblasts, the effect was milder with a 5.3% and 4.7% increase (GM08398 and GM09503, respectively). In GM05399 control fibroblasts, EGF stimulation did not affect TfR accumulation (Fig. 4H and Supplementary Material, Table S3). EGF stimulation did not alter the number of TfR-positive vesicles systematically. When EGF was added to the cell media, the average vesicle number increased in GM05399 and GM08398 fibroblasts but decreased in GM09503 and P1 fibroblasts compared with the vesicle numbers without EGF addition (Supplementary Material, Table S3 and Fig. S8). The EGF stimulation increased the size of the TfR-positive vesicles in all the fibroblasts. Still, the TfR-positive vesicles were the smallest in P1 fibroblasts in both conditions (Supplementary Material, Table S3 and Fig. S9).

As expected, we also observed some significant differences between the healthy donor fibroblasts using HC confocal microscopy (Supplementary Material, Table S4). These are likely explained by the individual genetic background and by age-dependent differences, given that the cellular phenotypes were most similar between the two school-aged controls. However, in most girdin-related analyses, the P1 had the most extreme cellular phenotype.

### Knockout of *CCDC88A* in healthy donor fibroblasts recapitulates the discovered phenotype of patient fibroblasts

**Figure 5:**
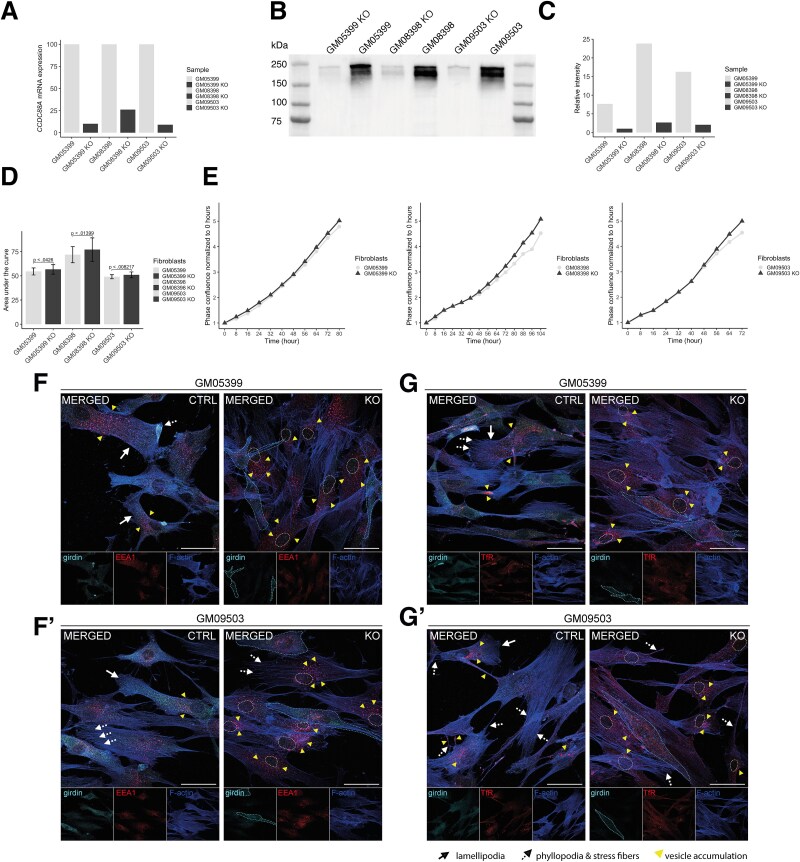
Silencing of *CCDC88A* with CRISPR-Cas9 gene editing recapitulates the phenotype observed in the patient fibroblasts. (A) RT-qPCR shows that gene editing silences *CCDC88A* mRNA expression. (B) Western blot image confirms that girdin (~220 kDa) expression in the fibroblasts is low. (C) The analysis of the normalized relative intensity values demonstrates girdin knockout efficiency in the fibroblasts. (D) Analysis of area under the curve (AUC) values of proliferation assay, *n* = 27–30 per fibroblast. Data are shown as mean ± SD and Mann–Whitney U-test was used. (E) Mean phase confluency normalized to the first image and changes in the confluency over time in three biological replicates in the proliferation assay. (F and G) Representative confocal microscopy images of endosomes and actin cytoskeleton in control and Girdin knockout fibroblasts, immunostained for Girdin (cyan), EEA1 (red) (F), and transferrin receptor (TfR) (red) (G), with F-actin (blue). The controls are represented by GM05399 (F and G) and the knockout by GM09503 (F′, G’). Images are representative of 10 different fields of view imaged per coverslip. Scale bar = 50 μm. Arrows indicate lamellipodia structures, dashed arrows point to filopodia and stress fibers, and yellow arrowheads highlight vesicle accumulation. Independent channel images are shown for each marker. In the knockout merged images, Girdin-positive cells are outlined with a dashed line. The knockout cell nucleus is marked with a dashed line.

To confirm that the increased proliferation rate observed in the P1 fibroblasts was due to girdin deficiency, we generated *CCDC88A* KO models by editing three control fibroblast lines using CRISPR-Cas9. RT-qPCR showed a KO efficiency of 90% (GM05399), 74% (GM08389), and 91% (GM09503) after 5–13 days of electroporation (Fig. 5A). Western blot confirmed that girdin expression was reduced by 87% (GM05399), 89% (GM08389), and 87% (GM09503) fibroblasts (Fig. 5B and C). All three girdin KO fibroblast models show an elevated proliferation rate compared with the wild-type (Fig. 5D and E), replicating the cellular phenotype of P1 and indicating that the excessive proliferation is caused by loss of girdin. To assess cellular phenotypic alterations in primary fibroblasts lacking girdin, we performed immunostaining for girdin, EEA1, TfR, and Phalloidin to visualize the actin cytoskeleton using confocal microscopy (Fig. 5; Supplementary Material, Table S1 and Fig. S10). Images were acquired from both control and KO fibroblast lines GM05399 (Fig. 5F and G) and GM09503 (Fig. 5F and G).

In migrating fibroblasts, the typical phenotype is characterized by an elongated cell shape with a leading edge in the direction of movement, enriched in fibrillar actin structures known as lamellipodia [35]. Here, vesicle trafficking is highly active, as this region is a site of active endocytosis that facilitates extracellular matrix removal, a process essential for cell migration [36]. Indeed, control fibroblasts appear polarized, with EEA1-positive vesicles predominantly localized near the leading edge of lamellipodia structures (Fig. 5F-F). In KO cells, these structures are largely absent, with a noticeable accumulation of vesicles observed in the perinuclear region, consistent with findings in P1.

In wild-type cells, TfR immunoreactivity demonstrates a normal subcellular distribution, with recycling endosomes polarized toward the leading edge of migrating fibroblasts [37]. TfR is also present in filopodia, membrane regions undergoing active recycling, and cell-to-cell contact points (Fig. 5G-G). In girdin KO cells, however, TfR-positive vesicles are abnormally concentrated near the nucleus, with marked variability in aggregate size.

It is noteworthy that girdin immunoreactivity appears dynamic in wild-type cells but is highly concentrated in areas of actin cytoskeleton remodeling (Fig. 5F and G). Furthermore, girdin KO cells exhibit a pronounced disruption of the actin cytoskeleton, characterized by severely weakened stress fibers, disorganized cortical actin, and a near-complete absence of lamellipodia and filopodia (Fig. 5F-F and G-G). As these structures are essential for the contractile ability of fibroblasts during movement [38], these findings are consistent with our earlier observations of impaired migration in patient-derived cells (Supplementary Material, Fig. S10).

### Immunophenotyping suggests immune defects due to girdin deficiency

As defects in cytoskeletal actin are known to cause inborn errors of immunity [39], and the patients were prone to infections, we set out to investigate their infection history and immune cell distribution and function in more detail. Both patients have been hospitalized due to recurrent pneumonias and have had over 30 infections requiring antibiotic treatment: at the age of 15 years, P1 has had 9 otitis media, at least 11 pneumonias, as well as upper respiratory tract infections. He was hospitalized for 9 days due to simultaneous infection with varicella zoster and adenovirus at 6 years 2 months of age. Patient 2 (P2) has had 18 otitis media, at least 6 pneumonias, upper respiratory tract infections, and 3 skin infections by 11 years of age. In addition, P2 developed inflammatory bowel disease at the age of 8 years and was treated with regular azathioprine and courses of prednisolon.

In immune cells, girdin is most highly expressed in monocytes, macrophages, and dendritic cells of the myeloid lineage and least expressed in T cells [18]. Consistently with this expression pattern, immunophenotyping showed that both patients had a reduced number of monocytoid and plasmacytoid dendritic cells (Supplementary Material, Table S5). Both patients had normal levels of IgA and IgE but IgG levels were elevated in P2 (Supplementary Material, Table S5). However, their response to the pneumococcal vaccine was poor (Supplementary Material, Table S5), and B cell differentials indicated reduced total B cell number in P2 and decreased percentages of switched memory B cells and plasmablasts in P1 (Supplementary Material, Table S6). T cell immunophenotyping showed that the patients had a normal total number of T cells, but the percentages of T effector memory (TEM) cells were reduced (Supplementary Material, Tables S5 and S6). Flow-based analyses of patient samples also revealed a reduced percentage of regulatory T cells (Treg) (Supplementary Material, Fig. S11, Table S5), but their capacity to suppress the proliferation of effector T cells (Teff) was normal (Supplementary Material, Fig. S12, Table S5). To further analyze T cell functionality, T cell proliferation was studied by stimulating the patient and the control peripheral blood mononuclear cells (PBMCs) for 4 days with either phytohemagglutinin (PHA) or anti-CD3/CD28-coated beads (Supplementary Material, Fig. S13). The percentage of both CD4^+^ and CD8^+^ patient T cells that started to proliferate in response to stimuli was similar to controls except for a somewhat weak response to PHA. The proliferation index, which indicates the average number of divisions per dividing cell, was also lower than that of controls upon PHA stimulation. In addition, the T cells of P2 went through fewer divisions in response to anti-CD3/CD28 bead-stimulation compared with controls, whereas the response of the T cells of P1 was similar to controls. These results may have been affected by the lingering effects of cortisone medication (P2) or the high number of naïve T cells present in patient samples compared with control samples. Clinical re-testing without cortisone medication of (P2) CD4^+^ and CD8^+^ T cells showed normal proliferation responses to both PHA and ConA mitogens in both patients (Supplementary Material, Table S5). Based on the upregulation of activation markers CD69 and CD25 at 16 and 40 h after stimulation, T-cell activation in girdin-deficient patient samples was normal (Supplementary Material, Fig. S14). Taken together, girdin deficiency does not prevent T cell activation and proliferation, but girdin deficiency may prolong the time needed for the completion of division cycles. However, the differences now observed between patients and controls may also have been due to secondary causes.

## Discussion

We studied two siblings with MCD, microcephaly, epilepsy, profound ID, and immunodeficiency. Using WGS, we found that both affected siblings were compound heterozygous for a rare missense variant p.Asp310Ala and a novel intragenic deletion encompassing three exons (Ex14–16) of *CCDC88A,* which encodes girdin. Both variants are in the coiled-coil domain of girdin. We characterized primary cells from these patients using several cellular assays, and the findings revealed many previously reported, girdin-associated phenotypes in the cells. Full-length girdin containing the missense variant was expressed at a level compatible with a heterozygous allele, and a truncated protein was expressed at a minimal level in P1 fibroblasts. P1 fibroblasts had high proliferation but a low migration rate when compared with healthy fibroblasts. P1 fibroblasts were also smaller in size, their cytoskeletal actin filaments were aggregated and sparse with few crosslinks, and organelles of endolysosomal pathway accumulated in the perinuclear region compared to controls. Furthermore, the cargo (TfR) accumulated to endosomes in an EGF-dependent manner in P1 fibroblasts. The cellular phenotypes were recaptured in CRISPR-Cas9 edited girdin KO fibroblasts. In addition to previous clinical descriptions of patients with truncating girdin variants [26–29], both patients were prone to infections requiring hospitalization and antibiotic treatment and had a low number of monocytoid and plasmacytoid dendritic cells and Tregs. This suggests that in addition to MCD, girdin-deficient patients may suffer from actinopathy-related inborn error of immunity.

Exonic *CCDC88A* deletions are rare: Database of Genomic Variants does not list any Gold Standard deletions overlapping any of the *CCDC88A* exons and gnomAD structural variants v4.0 lists two heterozygous deletions, that both have MAF of less than 0.00008. Homozygous truncating *CCDC88A* variants have been previously published in four families with variable details of phenotype description [26–29]. As the *CCDC88A* gene-disease validity awaits evaluation by the Clinical Genome Resource (ClinGen) [40] our report corroborates the gene-disease association. MCDs are heterogeneous disorders with heterogeneous genetic etiology, therefore, WGS is a powerful tool in resolving molecular genetic diagnoses of MCD. By identifying the small intragenic deletion in *CCDC88A*, our findings underline the importance of including high-resolution CNV analysis in the diagnostics of MCD.

Our patients manifested with postnatal onset microcephaly and cortical malformations, including absent or reduced gyration, dysgyria, and polymicrogyria. We were unable to specifically classify this combination of cortical malformations into any of the categories for their classification proposed in 2020 [41], but the phenotype shows features of ‘microcephaly with simplified gyral pattern’, as well as ‘microcephaly with polymicrogyria or dysgyria’. The normal thickness of the cortex differentiates the phenotype from classical lissencephaly spectrum malformations. Tubulinopathies manifest with a wide spectrum of MCD, but also non-cortical malformations: basal ganglia dysplasia (the hallmark of tubulinopathies), agenesis of the corpus callosum, enlarged tectum, as well as brain stem and cerebellar hypoplasia [42]. These non-cortical malformations are not present in our patients except for hypoplasia of the pons, and a thin corpus callosum. The similarity of our patients’ MRI findings in comparison to previously described patients with *CCDC88A* variants is striking [26], even though, ‘agyria’ and ‘pachygyria’ as descriptive terminology were previously used instead of absent or reduced gyration. The combination of absent or reduced gyration and polymicrogyria in the absence of pathognomonic features of tubulinopathies, should warrant a consideration of the *CCDC88A* variants. The pathogenetic mechanisms leading to reduced gyration in *CCDC88A*-associated disease remain to be elucidated in the future.

The neurological phenotype in our patients appeared very similar to the previously reported patients [26] with profound psychomotor retardation and epilepsy, but the onset of microcephaly differed from congenital [26] to postnatal microcephaly in our patients and the patient reported in 2023 [27]. The later onset microcephaly in our patients might reflect a partial loss-of-function of the missense variant or could be explained by other genetic factors. Other genetic factors most likely play a role, as the onset of microcephaly also differs between the previously reported families with truncating *CCDC88A* variants [26, 27]. We consider that the optic atrophy in our patients is most likely caused by retrograde axonal degeneration [43], and is a secondary consequence of the severe cortical defects, although it is impossible to exclude a primary pathology of the optic nerve. Moderate optic atrophy has also been described in conjunction with severe cortical visual inattention in previously reported patients [26], whereas the fundus appeared normal in one patient with early death [27]. The similarity of the *CCDC88A*-associated phenotype to progressive encephalopathy with edema, hypsarrhythmia, and optic atrophy (PEHO) syndrome caused by variants in *ZNHIT3* [44] was previously discussed [26]. Although the clinical features of patients with *CCDC88A* variants resemble clinical features of PEHO, the brain MRI findings are distinct: the cortical malformations, reported by us and previously [26, 27], are not compatible with a diagnosis of PEHO syndrome [45]. These findings highlight cortical malformations as an important feature in *CCDC88A*-associated disorder and emphasize the importance of including *CCDC88A* in the differential diagnostics of MCDs with prenatal or postnatal microcephaly.

We took advantage of patient-derived fibroblasts for molecular definition and functional phenotyping of the disease. As shown previously [26], we also observed an incomplete nonsense-mediated decay for the truncated allele using RT-PCR in the P1 sample. Previously [26], the Human Embryonic Kidney 293 (HEK293) cell line with girdin (p.Leu772*) allele was used to detect the expression of a truncated protein in an over-expression model. Because the study was not done with patient-derived cells, it could not be concluded if truncated (p.Leu772*) girdin was expressed *in vivo*. We showed that the allele with the intragenic *CCDC88A* deletion results in nearly non-detectable expression of truncated girdin (p.Glu508*) in the P1 fibroblasts. It is difficult to draw a definitive conclusion, about potential biological significance of the minimally expressed truncated girdin protein, whether it acts as a null allele or interferes with the function of the other allele. Dominant-negative effects are known to appear in proteins that dimerize and oligomerize, such as girdin. The expression of the other girdin allele with the p.Asp310Ala in P1 fibroblasts resulted in a full-length protein with an amount that is compatible with the expression of a heterozygous allele. The p.Asp310Ala is not located in any of the known binding domains but it is located at the coiled-coil domain of girdin [1], known to support the dimerizing and oligomerizing of the protein. Given that the parents of the studied family attended a mainstream school with curriculum adjustments due to their learning difficulties, it is possible that both alleles independently show some carrier manifestations. Indeed, heterozygous carriers are known to present milder phenotypes of some Mendelian disorders that were traditionally considered recessive [46].

As regulation of the proliferation-migration dichotomy is one of the main functions of girdin [30, 32], we performed both proliferation and wound-healing assays using P1 fibroblasts to assess the variants’ effect on this regulation. P1 fibroblasts having girdin variants migrated slower than control fibroblasts. The expression of the full-length girdin with the critical C-terminal domains from the missense variant allele might help to maintain a certain level of migration-related functions of girdin. This is consistent with the data showing that siRNA-mediated knockdown of girdin leads to decreased migration in many cell types, e.g. monkey kidney epithelial cells (Vero cells), human umbilical vein endothelial cells (HUVECs), human vascular smooth muscle cells (hVSMCs), and human cardiac fibroblasts [1, 24, 31, 47]. Interestingly, cell proliferation was markedly increased in P1 and recapitulated in girdin KO fibroblasts. The increase in proliferation was not as drastic using the KO model as it was in the P1 fibroblasts. Although girdin was silenced efficiently (KO efficiency 87–89%), there was still some functional girdin left in the KO cells. This could tone down the proliferation phenotype compared with P1 fibroblasts that might not express functional girdin at all. Inhibited migration and enhanced mitosis have been observed in HeLa cells when C-terminal binding domains of girdin were deleted [32]. A dominant-negative splicing variant leading to truncated girdin was also observed in rapidly proliferating poorly invasive cancer cells, whereas highly motile invasive cancer cells express full-length girdin [32]. It is possible that the minimal amount of truncated girdin in P1 fibroblasts could facilitate a similar dominant-negative proliferative effect as discovered in studies of cancer cells [32].

Quantitative phenotypic analysis of P1 fibroblasts revealed that they were smaller in size than the control fibroblasts. This can be affected by increased cell proliferation, or by the growth rate regulation. The cell size was not affected in HUVECs when girdin was silenced with siRNA [24] but the knockdown of drosophila girdin resulted in reduced Drosophila wing cell size downstream of Akt signaling [13]. Although drosophila girdin is an ortholog for human girdin, their C-terminal parts are divergent, and therefore drosophila might not be the best model organism for human girdin studies [13]. In mice, overexpression of girdin in neurons led to increased soma size and number of dendrites *in vivo* and this effect was reversed by treatment with mTOR inhibitor rapamycin [12]. Thereby, it is possible that our variants could negatively regulate the cell size through Akt/mTOR signaling. Furthermore, a possible relationship between increased cell proliferation and cell size decrease would warrant future studies.

Unlike in the control fibroblasts, the membrane region compared with the total cell area was reduced in P1 fibroblasts. This finding is in line with the previous studies as silencing of girdin with siRNA caused the shortening of lamellipodia around the hVSMCs upon platelet-derived growth factor stimulation [47] and around the HUVECs upon vascular endothelial growth factor (VEGF) stimulation [24]. In our experiment, the fibroblasts were stimulated with fetal bovine serum (FBS), recombinant human fibroblast growth factor basic (rhFGF-b), ascorbic acid, L-Glutamine, Hydrocortisone Hemisuccinate, and human recombinant (rh) insulin. As previously observed in hVSMCs [47] and Vero cells [1], the cytoskeletal actin remodeling was altered in P1 and girdin KO fibroblasts compared with controls. The cytoskeletal actin network in P1 and KO fibroblasts was sparse and aggregated. A similar sparse actin network with fewer crosslinks to other actin molecules was detected by electron microscopy imaging in Vero cells when girdin was silenced with siRNA [1]. We also observed fewer focal adhesion structures in P1 fibroblasts than in control fibroblasts as reported earlier in Cos7 cells by shRNA girdin depletion [34].

Finally, we found that early endosomes (EEA1 marker) and lysosomes (LAMP1, LAMP2, LIMP markers) had a reduced number and they accumulated in the perinuclear region of P1 fibroblasts, whereas in control fibroblasts, they were spread throughout the cytoplasm. Accordingly, the cargo of this pathway, TfR, was accumulated. These results were also recapitulated in girdin KO fibroblasts. Variants in components of the autophagy-lysosomal pathway are known to lead to neurodevelopmental disorders with epilepsy; reviewed in [48]. The accumulation of the autophagy-lysosomal pathway components in a cell is observed in many neurodevelopmental diseases [48]; for example, variants in *CLN3* cause neuronal ceroid lipofuscinosis and a specific accumulation of lysosomes in the perinuclear region have also been detected in CLN3 deficits [49]. Because neurons are extremely asymmetric and axons and dendrites are long, regulation of lysosome dynamics is especially crucial in neurons. Therefore, we speculate that the observed phenotype in P1 fibroblasts could be even more aggravated in neurons. Interestingly, we observed an enhanced TfR accumulation in the perinuclear region of the P1 fibroblasts when stimulated with EGF. TfR is an iron transporter, and it is important for neuronal cells. TfR-KO mice manifest progressive epileptic seizures and abnormal synapses [50]. Future studies of girdin-TfR interplay in neurons are needed. Girdin regulates autophagy by either inducing or inhibiting it: when cells are starved, girdin induces autophagy by inhibiting the G protein, and when cells are stimulated with growth factors, girdin inhibits autophagy by activating the G protein [20]. A previous study has shown that girdin depletion with siRNA in HeLa cells leads to EGFR accumulation in EEA1 endosomes, EGFR signaling prolongation, and enhanced proliferation [30]. Taken together, imaging-based phenotyping data shows that P1 cells manifest several phenotypes linked to girdin deficiency. This confirms that the compound heterozygote variants identified in our patients lead to full or partial loss of girdin function and result in defects of cell size regulation, actin cytoskeleton, focal adhesions, and endolysosomes.

Defects in cytoskeletal actin are known to cause inborn errors of immunity i.e. immune-related actinopathies; reviewed in [39]. Motility is crucial for immune cells to ensure their access to organs and tissues where their response is needed. As girdin is an actin-remodeling protein essential for macrophage chemotaxis [17] and actin assembly at the immunological synapse in Jurkat cells [19], it was not surprising that the patients had clinical signs of immunodeficiency even though the previous publications have not described them. Both patients consistently showed a reduced number of monocytoid and plasmacytoid dendritic cells, in which girdin is highly expressed [18], further indicating the importance of girdin for the function and motility of these cell types. Both patients had low counts of Treg, although they maintained their suppression capability. Patient-derived T cells did not show any significant activation or proliferation defects. Patients also had poor polysaccharide vaccine response suggesting aberrant B cell function. The B and T cell abnormalities could potentially be due to the low number or functional defectiveness of dendritic cells, which as antigen-presenting cells (APC) adjust B and T cell functions in adaptive immunity, or due to reduced motility or defective immune synapse formation as observed in other immune-related actinopathies [39]. P2 has been treated with corticosteroids and azathioprine for increased fecal calprotectin and symptoms compatible with inflammatory bowel disease. Interestingly, girdin deficiency led to hyperreactive immune responses by gut macrophages and increased the severity of inflammation in the dextran sodium sulfate -induced colitis mouse model which also manifested reduced colon length and increased weight loss [18]. Both dendritic cells (in steady state) and Tregs are key players in maintaining homeostasis and tolerance in the gut. Further investigation of the potential contribution of girdin deficiency in the immune and gut phenotypes warrants further studies.

Azathioprine can halt the proliferation of fast-dividing cells, reduce the numbers of circulating B and T lymphocytes, specific NK cell subpopulations, and dendritic cells [51]; and could thus influence the lymphocyte profile in P2. Importantly, however, the main immune cell deficiencies, i.e. low numbers of monocytoid and plasmacytoid dendritic cells, and Tregs, were observed in both patients, P1 not receiving immunosuppressive treatment. Based on the known function of girdin in remodeling the actin cytoskeleton and its expression in immune cells, it is conceivable that it has a role in immune cell function, particularly affecting dendritic cell motility, immune synapse formation and functions of innate immune cells. Defects in any of them can compromise B cell activation, antibody production, and activation and maintenance of Tregs. Future studies are needed to further characterize immunological phenotypes and potential pathogenic mechanisms of girdin deficiency, but our results highlight the importance of examining the infection history and immunological phenotype of girdin deficient patients to avoid immunological features being overlooked in patients with MCD, similarly as in other severe inherited brain malformations [52].

In conclusion, we describe here the first *CCDC88A* missense variant (p.Asp310Ala) and intragenic deletion (exons 14–16) underlying MCD. Our results underline the importance of considering *CCDC88A*—including the CNVs—in the diagnostics of MCDs. We show that these compound heterozygous variants cause girdin dysfunction in P1 fibroblasts, i.e. alterations in morphology and migration-proliferation dichotomy, and characterize defects in the immunity of these patients. Future studies are needed for p.Asp310Ala variant located at the coiled-coil domain of girdin: they might provide new insights into the structure of girdin or a potential interplay between different domains of this multimodular protein.

## Materials and methods

### Ethical approvals and patient consent

The study was approved by the Ethics Review Board of the Helsinki University Hospital and the Norwegian Research Ethical Committee (REK ID: 77492) and performed according to the Declaration of Helsinki. Written informed consent was collected from the family members and the parents signed it for the under-aged children as their legal guardians. All study methods were performed following relevant guidelines and regulations.

### Clinical characteristics

**Figure 6:**
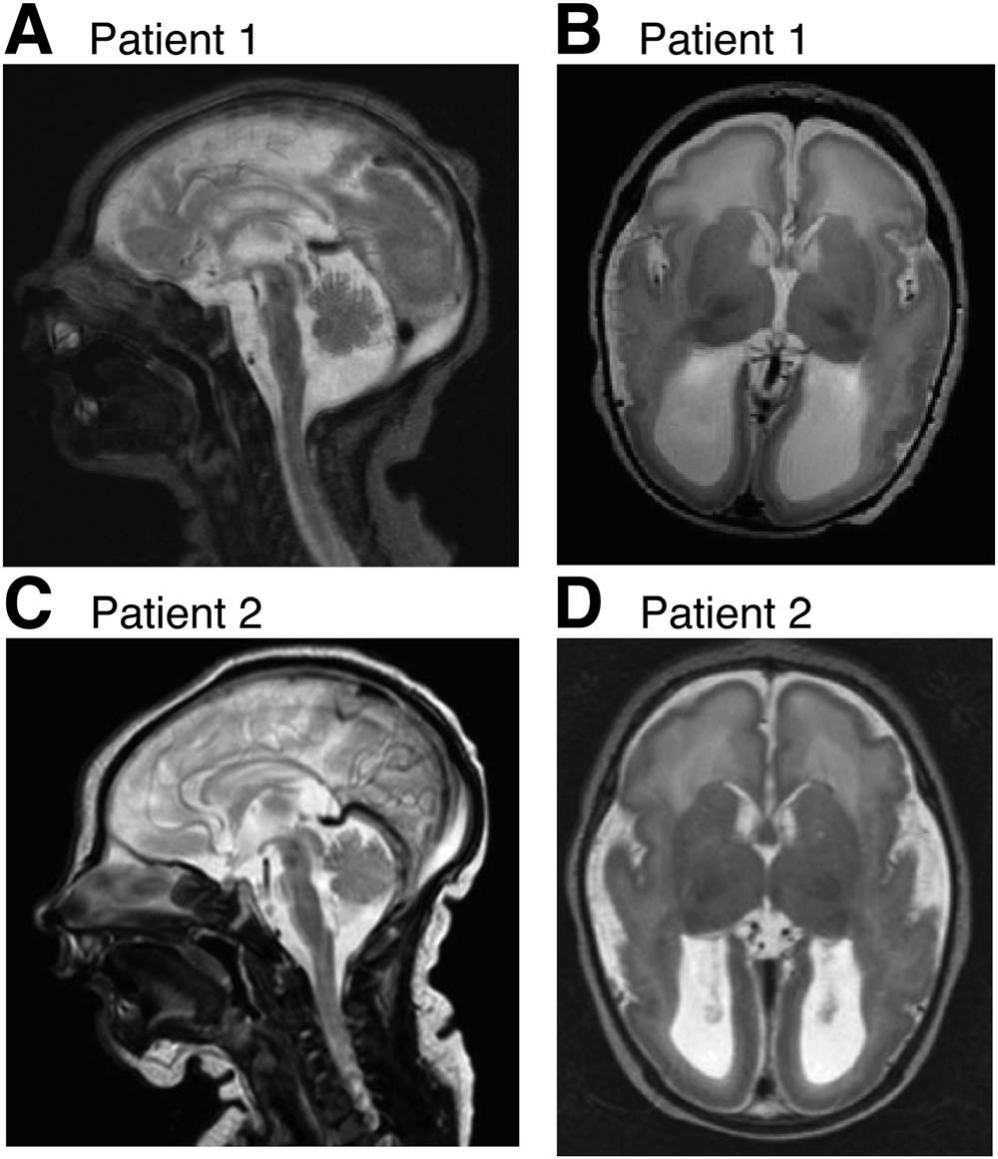
MRI images. (A and C) T2 weighted sagittal image shows a thin corpus callosum with hypoplastic splenium. It also shows hypoplastic pons and normal vermis. (B, D) T2 weighted axial image shows posteriorly enlarged ventricles with intraventricular cysts. There is temporo-parietal polymicrogyria, no cortical folding in the occipital lobes, and there is a simplified gyral pattern in the frontal lobes. Subcortical band heterotopia is seen posteriorly. The images were taken at the age of four days (patient 1) or five days (patient 2).

The patients are two siblings (male and female) born at term to non-consanguineous Finnish parents. The clinical features of the two siblings are summarized in Table 1 and described in detail in the Supplementary Material. The immunophenotype of the patients is presented in Supplementary Table S5 and T and B lymphocyte flow cytometry results are in Supplementary Table S6. Shortly, the phenotype included secondary and progressive microcephaly (HP:0005484; HP:0000253), profound ID (HP:0002187), cerebral visual impairment (HP:0100704), absent speech (HP:0001344), abnormal central motor function (HP:0011442), neonatal hypotonia (HP:0001319), later onset spasticity (HP:0001257), seizures (HP:0001250) that were early onset and recurrent despite polytherapy, recurrent acute respiratory tract infections (HP:0011948), scoliosis (HP:0002650), as well as cryptorchidism (HP:0000028) in the male patient (P1) and colitis (HP:0002583) in the female patient (P2). The brain MRI findings (Fig. 6A–D) included abnormal cortical gyration (HP:0002536) with absent or reduced gyration, dysgyria and polymicrogyria (depending on the cerebral region), hypoplasia of the pons (HP:0012110), lateral ventricle dilation (HP:0006956), and a thin corpus callosum (HP:0033725). A repeat brain MRI of P1 at 7 years 11 months of age also showed bilateral lesions of unknown cause that had developed after the previous MRI (Supplementary Material, Fig. S15).

**Table 1:** Clinical features and brain MRI findings of the two siblings compared with previously reported patients [26–28].

Clinical features	Patient 1	Patient 2	Nahorski et al. (P1)	Nahorski et al. (P2)	Nahorski et al. (P3)	Abdulkareem et al. (*n* = 2)	Issa et al. (*n* = 1)
Ventriculomegaly (detected at)	>30 weeks of pregnancy	>30 weeks of pregnancy	NR	NR	>20 weeks of pregnancy	NR	NR
Microcephaly	Secondary and progressive	Secondary and progressive	Primary and progressive	Primary and progressive	Primary and progressive	Yes	Secondary and progressive
Intellectual disability	Profound	Profound	Profound	Profound	Profound	Yes	Profound
Speech	Absent	Absent	Absent	Absent	Absent	Absent	Absent
Motor function	Wheelchair with head support, voluntary movements of upper extremities	Wheelchair with head support, voluntary movements of upper extremities	No progress, physically handicapped	Quadriplegia	Severe motor delay	Cannot walk	Did not achieve any motor milestones
Hypotonia	Neonatal	Neonatal	Neonatal (moderately severe, central)	Neonatal (moderately severe, central)	Neonatal (moderately severe, central)	Yes	Yes (severe axial hypotonia, no head support, hypotonic extremities)
Hypertonia/Spasticity	Spasticity (at 1 years 7 months)	Spasticity (at 11 months)	Hypertonia (Peripheral)	Hypertonia (Peripheral)	Hypertonia (Peripheral)	NR	NR
Cerebral visual impairment (= cortical visual impairment)	Severe (Reaction to light and high-contrast pictures)	Severe (Reaction to light and high-contrast pictures)	Severe	Severe	Severe	Delays in vision; poor visual responsiveness	Failure to follow objects or light
Seizures (onset)	Medication at 7 months	<24 h	First week of life	First day	<3 h	Early age	Second month
Recurrent infections	Pneumonias, otitis media, respiratory infections, simultaneous varicella zoster and adenovirus infections	Otitis media, pneumonias, respiratory infections, skin infections	NR	NR	NR	NR	NR*
Cryptorchidism	Yes	NA	Yes	NR	NA	NR	NA
Scoliosis	Yes	Yes	Yes	MI (in 2/3 patients reported by Nahorski et al.)	MI (in 2/3 patients reported by Nahorski et al.)	NR	NR
Colitis	No	Yes	NR	NR	NR	NR	NR
Peripheral edema	Yes	Yes	Yes	Yes	Yes	Yes	Yes
**Brain MRI findings**	**Patient 1**	**Patient 2**	**Nahorski et al. (P1)**	**Nahorski et al. (P2)**	**Nahorski et al. (P3)**	**Abdulkareem et al. (*n* = 2)**	**Issa et al. (*n* = 1)**
Abnormal cortical gyration	Yes (simplified gyral pattern, polymicrogyria, posterior double cortex)	Yes (simplified gyral pattern, polymicrogyria, posterior double cortex)	Yes	Yes	Yes	MI	Yes (abnormal gyral pattern with simplified areas and increased in others)
Hypoplasia of the pons	Yes	Yes	Yes	Yes	Yes	MI	Yes
Lateral ventricle dilatation	Yes	Yes	Yes	Yes	Yes	MI	Colpocephaly
Corpus callosum	Thin	Thin	Thin	Thin	Abnormal morphology	MI	Hypogenesis
Other MRI findings	Intraventricular cysts; Bilateral parietal gliotic changes of unknown etiology (at 7 years 11 months)	Intraventricular cysts	Reduced amount of white matter	Subependymal cysts	Cerebellar hypoplasia	Brain atrophy, dysmorphic features	Prominent basal ganglia, increased white matter signal

The patient reported in [29] is not included in the table as no detailed clinical information was available (the patient was only listed in the supplementary table of the article). NA = not applicable (due to female sex); NR = not reported in the original article; MI = unable to evaluate the presence of this feature due to missing information (MI). (*) Additional four siblings of the patient reported by [27] most likely manifested with the *CCDC88A*-associated disease and died of unexplained illnesses at 3–4 months of age. Whether infectious etiology played a role was not mentioned in the article.

### Genome sequencing

Genomic DNA was extracted from EDTA-blood samples using standard protocols. WGS was performed for the affected siblings and their parents. Sequencing libraries were prepared using the KAPA HyperPlus Kit (Roche, Basel, Switzerland) according to the manufacturer’s instructions at the Institute of Molecular Medicine Finland (FIMM), Helsinki, Finland. The sequencing was performed on NovaSeq 6000 platform (Illumina, San Diego, CA, USA). The sequencing data was aligned on GRCh37. Sequencing reads were analyzed using an in-house developed variant calling pipeline (VCP) 3.7 for quality control, short-read alignment, variant identification, and annotation [53].

### Variant identification

The analysis was primarily targeted at exonic and splicing variants. Synonymous variants, variants with MAF > 0.01 in gnomAD, and variants occurring frequently in our in-house database were filtered out. Homozygous, compound heterozygous, and heterozygous variants shared by the affected siblings in genes associated with a disease in Online Mendelian Inheritance in Man Database (OMIM) were interpreted. In the variant prioritization, *in silico* prediction tools were used. If a REVEL score was available, we only analyzed those variants with a REVEL score > 0.5. Intronic variants of the most interesting candidate genes were evaluated, and the WGS data was visually examined with IGV. We also analyzed the data using a virtual gene panel for brain malformations including 230 genes (Supplementary Material, Table S7). The gene panel was constructed based on searches in the Human Gene Mutation Database (HGMD, Professional 2019.1), OMIM, and genes reviewed previously in the context of the MCD [54] and polymicrogyria [55].

### Copy number variant analysis

To confirm the heterozygous intragenic *CCDC88A* deletion observed visually in the WGS data, we ran our in-house developed CNV analysis pipeline (copyCat, manuscript in preparation) for the patients and parents. WGS data of the family members were run against a control group formed from four genomes with similar read depths. CopyCat compares Reads Per Kilobase of exon model per Million mapped reads (RPKM) normalized [56] coverage signals between a test sample and a median of the control samples. The coverage signals were generated from read-depth information of partitioned genomic regions (here 1000 bp), and individual genomic partition was transformed to CNV calls by using a circular binary segmentation (CBS) from the R package DNAcopy [57]. The CNV analysis included autosomal chromosomes and was targeted on the gene panel for brain malformations having 230 genes (Supplementary Material, Table S7). After annotation of the CNV regions, we filtered the CNV calls in the following way: analysis covered only calls that were shared between both affected siblings, and all the CNVs containing > 50% of segmental duplications were filtered out. All the remaining CNV calls (five heterozygote deletions) were visualized on IGV and due to observed SNV heterozygosity and lack of paired-end anomalies in the called regions, most of them were excluded as false positive CNV calls (three). For the residual CNVs (two) we performed a split-read analysis to identify the exact DNA breakpoints. The other, a heterozygous deletion in *SLC25A24* (chr1:108733326–108 737 251, hg19 coordinates) was filtered out due to a high occurrence in the gnomAD structural variants v4.0 database (MAF = 0.2357), resulting in the one novel shared heterozygote deletion in *CCDC88A*.

### Variant validation

Capillary sequencing for both the missense variant (NM_001135597.1:c.929A > C, p.Asp310Ala) and the intragenic *CCDC88A* deletion (including exons 14–16) was performed to verify the findings and to study the segregation. The Sanger sequencing primers are listed in Supplementary Table S8. The missense variant fragment was amplified using the touchdown program: 98°C for 30 s (1 cycle); 98°C for 10 s and 72–58°C for 30 s and 72°C for 15 s (15 cycles); 98°C for 10 s and 58°C for 30 s and 72°C for 15 s (17 cycles); 72°C for 10 min, 10°C hold with Phusion DNA polymerase (Thermo Fisher Scientific, Waltham, MA, USA). The fragment containing the deletion was amplified with: 95°C for 3 min (1 cycle); 95°C for 30 s and 62°C for 30 s and 72°C for 30 s (32 cycles); 72°C for 10 min, 10°C hold with DreamTaq DNA polymerase (Thermo Fisher Scientific). The sequencing reactions were run on ABI3730XL DNA Analyser (ABI BigDye 3.1, Applied Biosystems, Waltham, MA, USA), and the sequences were analyzed using Sequencher (Gene Codes Corporation, Ann Arbor, MI, USA).

### Cell culture

All healthy control human primary dermal fibroblasts (1620, 3008; Sigma Aldrich, St. Louis, MO, USA) and (GM00409, GM05399, GM08398, GM09503; Coriell Institute, Camden, NJ, USA) were purchased commercially. Fibroblasts 1620 and 3008 were derived from adults, and gender-matched GM00409, GM05399, GM08398, and GM09503 cell lines were from individuals who were seven, one, eight, and ten years of age at sampling, respectively. The patient was five years old when the skin biopsy sample was taken. All fibroblasts were routinely cultured in humidified incubators at 37°C under 5% CO_2_. Cells were cultured using FibroGRO™ -LS Complete Media Kit (#SCMF002; Millipore, Burlington, MA, USA) thus basal media was supplemented with 5 ng/mL rhFGF-b; 50 μg/ml ascorbic acid; 7.5 mM L-Glutamine; 1 μg/ml Hydrocortisone Hemisuccinate; 5 μg/ml rh insulin; 2% FBS and 1% Penicillin–Streptomycin. The cell lines were routinely tested for mycoplasma contamination with LookOut Mycoplasma PCR Detection Kit (#D9307; Sigma Aldrich).

### Reverse transcription polymerase chain reaction analysis

RNA was extracted from primary dermal fibroblasts of P1 and a control cell line (1620; Sigma Aldrich) using miRNeasy Kit (Qiagen, Hilden, Germany). First-strand cDNA was synthesized using SuperScript VILO Kit (Invitrogen/Thermo Fisher Scientific). The primer information is listed in Supplementary Table S8 (spanning exons 8–12, CCDS46288.1). The RT-PCR was conducted with the program: 95°C for 3 min (1 cycle); 95°C for 30 s and 60°C for 30 s and 72°C for 40 s (32 cycles); 72°C for 10 min, 10°C hold with DreamTaq DNA polymerase (Thermo Fisher Scientific). The sequencing reactions were run on ABI3730XL DNA Analyser (ABI BigDye 3.1, Applied Biosystems) and the sequences were analyzed using Sequencher (Gene Codes Corporation).

### Western blotting

Whole-cell proteins were extracted by lysis of primary dermal fibroblasts of P1 and the control cell lines (GM00409, GM05399, GM08398, GM09503) in RIPA buffer (Thermo Fisher Scientific) with protease inhibitors and EDTA (#87786; Thermo Fisher Scientific). The total protein concentration was determined using the Pierce BCA Protein Assay Kit (Thermo Fisher Scientific). 10 μg of total protein sample was loaded on 4%–15% Mini-PROTEAN TGX Precast Protein Gels (Bio-Rad, Hercules, CA, USA), and proteins were transferred to low fluorescence PVDF membranes (Bio-Rad) with Trans-Blot Turbo Transfer System (Bio-Rad). Total protein staining was performed with No-Stain Protein Labeling Reagent (Invitrogen/Thermo Fisher Scientific). Membranes were blocked for 1 h in 5% milk in 0,1% TBST and followed by overnight incubation of primary antibody against N-terminal girdin (1:1000; ab179481; Abcam, Cambridge, UK) and 1-h incubation of a secondary antibody Swine Anti-Rabbit IgG/HRP (1:5000; P0399; Dako, Santa Clara, CA, USA) or Goat Anti-Rabbit IgG/HRP (1:2000; #7074; Cell Signaling Technology, Danvers, MA, USA). ECL reaction was performed with SuperSignal West Femto Maximum Sensitivity Substrate (Thermo Fisher Scientific), and the imaging was done using ChemiDoc MP Imaging System (Bio-Rad). The relative intensity and the total protein normalization were determined with Image Lab Software (Bio-Rad).

### Fibroblast proliferation assay

Primary dermal fibroblasts of P1 and the control cell lines (GM05399, GM08398, GM09503) were seeded in 100 μl of growth media at a density of 1.5 K cells per well into 96-well culture plate (#3904; Corning, Corning, NY, USA). When the proliferation was compared between P1 and controls, fresh growth media was changed twice: 6 and 47 h after cell seeding. In the KO proliferation experiments, growth media was changed only at the later time point. The proliferation assay was performed for four technical repeats, acquiring data from 60 wells per individual (P1 versus controls), and for three biological replicates (KO experiments), acquiring data from 27–30 wells per fibroblast. Plates were placed into IncuCyte® ZOOM or IncuCyte S3 (Sartorius, Göttingen, Germany) and were scanned with 10x objective every 2 h. The phase confluence-based imaging analysis was performed with IncuCyte® Software (Sartorius). The analysis includes data starting when fibroblasts were 18%–27% confluent until the most rapidly proliferating cell line was 86% (P1 versus controls) and 92% (KO experiment) confluent. The data of every well was normalized to its confluence value at the analysis starting point. The area under the curve values and statistical differences were calculated for the confluence ratio values every 4 h.

### Wound-healing assay

Primary dermal fibroblasts of P1 and the control cell lines (GM05399, GM08398, GM09503) were seeded in 100 μL of growth media at a density of 30 K cells per well into 96-well ImageLock™ tissue culture plate (#4379; Essen BioScience, Ann Arbor, MI, USA). Cells were grown to 100% confluency, and cell proliferation was inhibited with mitomycin C treatment (8 μg/mL for 3–4 h before wound making). The scratches were made simultaneously to all wells 25–26 h after seeding with WoundMaker™ (Essen BioScience). After wounding, the cells were washed two times with PBS, and fresh growth media was added. The plates contained 12–15 wound replicates per cell line, and the experiment was repeated four times. The plates were placed into IncuCyte® ZOOM (Sartorius), and the wounds were scanned with 10x objective every 2 h. The imaging analysis was performed with IncuCyte® Software (Sartorius) and the data analysis included relative wound density (RWD) values until the 12 h time point.

### CRISPR-Cas9 gene editing

For CRISPR-Cas9 gene editing, *CCDC88A* Alt-R CRISPR-Cas9 sgRNA was designed using CHOPCHOP [58] and they are listed in Supplementary Table S8. RNPs were synthesized by assembling *CCDC88A* individual guides (each at 500 pmol/sample) with 310 pmol/sample of Alt-R^®^ S.p. Cas9 Nuclease V3 (#1081059; Integrated DNA Technologies, Coralville, IA, USA) and incubated at room temperature (RT) for 15 minutes. Fibroblasts from three healthy controls (GM05399, GM08398, GM09503) were cultured until confluence reached 80%. They were resuspended in an electroporation buffer of P2 Primary Cell 4D-Nucleofector^®^ X Kit L (#V4XP-2024; Lonza, Basel Switzerland) at a density of 1.5 million fibroblasts/100 μl. Fibroblasts were electroporated on 4D Nucleofector^®^ X Unit (Lonza) with the ‘DT-130’ electroporation program. After electroporation, 500 μl of FibroGRO™-LS complete media (#SCMF002; Millipore) was added per sample, and fibroblasts were allowed to rest for 15 minutes at 37°C. Subsequently, fibroblasts were transferred into T75 flasks in 15 ml medium at a density of 5 × 10^4^/mL. When confluence reached 80% post-electroporation, fibroblasts were harvested for qPCR and Western blot analyses to evaluate the knock-out efficiency.

### RT-qPCR

AllPrep DNA/RNA Mini Kit (Qiagen) was used for RNA extraction. cDNA was synthesized by iScript™ Advanced cDNA Synthesis Kit (Bio-Rad) using T100™ Thermal Cycler (Bio-Rad). PowerTrack™ SYBR Green Master Mix (Thermo Fisher Scientific) was used for followed qPCR on CFX Opus 96 (Bio-Rad). The 2^−ΔΔCT^ method was used to evaluate the efficiency of CRISPR-Cas9 gene knock-out. *GAPDH* was used as the housekeeping gene. Primers were ordered from Integrated DNA Technologies and are listed in Supplementary Table S8.

### Immunofluorescence staining and imaging

P1 and control fibroblasts (GM05399, GM08398, GM09503) were seeded at a density of 800 cells per well into 384-well imaging plates (#6057300; Perkin Elmer, Waltham, MA, USA). After the cells were attached to the plates, media was exchanged with FibroGRO™ -LS Complete Media Kit (#SCMF002; Millipore) with supplements listed in the ‘Cell culture’ paragraph. Parallel experiments were performed using media supplemented with 42 ng/mL of EGF (#354052). Subconfluent fibroblasts were fixed with 4% paraformaldehyde (Biotek MultiFlo FX, Agilent Technologies, Santa Clara, CA, USA) for 20 minutes at RT, and washed with PBS by plate washer (Biotek EL406, Agilent Technologies). Cells were permeabilized with 0.3% Triton X-100 in PBS for 10 minutes and blocked with 3% BSA in PBS for an hour. Primary antibodies (see Supplementary Material, Table S1 for antibodies and dilutions) were transferred to the plates and incubated for an hour at 37°C. After washing the cells with PBS, the secondary antibodies and additional stains (Alexa Fluor 568 anti-mouse; Alexa Fluor 647 phalloidin; and Hoechst 33242) were added to the wells and incubated for an hour at RT. Stained cells were imaged with Opera Phenix confocal microscope (PerkinElmer) at FIMM High Content Imaging and Analysis unit (FIMM-HCA) by using 20x water immersion objective (NA 1.0; 3 planes in z-stack; 9 frames/well, 5% overlap). Cells were segmented and cellular features were extracted and quantified with Harmony 4.9. software package (PerkinElmer) as shown in Supplementary Table S2. In short, the nuclei were segmented according to the Hoechst staining, and the total cell area was segmented according to the respective staining pattern of phalloidin. The cell area was divided to the perinuclear ring region expanded outwards from the nuclear membrane (100% of the nuclear area, masked by the total cell area), as well as to cytoplasmic and membrane regions covering 90% and 10% of the corresponding total cell region. The vesicular objects were segmented using a spot detection algorithm specified in Supplementary Table S2. Similarly, the intracellular filament structures were segmented from the total cell region with a separate spot detection algorithm according to the phalloidin staining. Data from each analysis was exported as single-cell values for statistical analysis and visualization.

### Immunocytochemistry and confocal imaging of Girdin KO cells

Control and Girdin-KO primary fibroblasts (GMO9503 and GMO5399) were thawed and seeded onto uncoated coverslips in 24-well plates at a density of 60 000 cells per well. The cells were cultured overnight in supplemented FibroGRO™-LS Complete Media Kit (#SCMF002; Millipore). The following day, cells were washed three times with 1X DPBS containing calcium and magnesium (#C14784, Thermo Fisher Scientific) before fixation, following the protocol described by [59], with minor modifications. Briefly, cells were permeabilized with 0.3% Triton X-100 for 10 minutes at RT and subsequently incubated for 30 minutes in blocking buffer (4% BSA in 1X DPBS) at RT. Next, the cells were incubated for 1 h at 37°C with primary antibodies against Girdin, TfR, EEA1, and LAMP-1, as listed in Supplementary Material, Table S1. After primary antibody incubation, the cells were washed with 1X DPBS for 10 minutes and then incubated for 30 minutes at 37°C with the appropriate secondary antibodies: Alexa Fluor 488 anti-mouse, Alexa Fluor 594 anti-rabbit, and conjugated Phalloidin-405 for actin cytoskeleton visualization. Confocal Z-stack images were acquired from 10 fields of view (FOV) per sample using a Zeiss LSM880 Fast AiryScan microscope equipped with a 63X oil-immersion objective. Images were processed using ImageJ software (National Institutes of Health, Bethesda, MD, USA) for background subtraction and the generation of maximum intensity projections.

### Flow-based immune cell phenotyping

For Treg analysis, surface stainings were done with anti–CD4-PerCP (#345770), anti–CD25-APC (#555434), and anti–CD127-PE (#557938) monoclonal antibodies from BD Biosciences, Franklin Lakes, NJ, USA. Intracellular staining was done with Alexa Fluor 488 FOXP3 monoclonal antibody (#320112, BioLegend, San Diego, CA, USA) and eBioscience™ Foxp3/Transcription Factor Fixation/Permeabilization Concentrate and Diluent (#00–5521-00, Invitrogen/Thermo Fisher Scientific).

### Treg proliferation and suppression assay

CD4+ cells were isolated from whole blood with RosetteSep™ Human CD4+ T Cell Enrichment Cocktail (Stemcell Technologies, Vancouver, bc, Canada) according to the manufacturer. The isolated CD4+ cells were stained with anti–CD4-PerCP (#345770), anti–CD25-APC (#555434), and anti–CD127-PE (#557938) monoclonal antibodies from BD Biosciences, and sorted with BD FACSAria™ IIIu cell sorter (BD Biosciences) to isolate CD4+ Teffs (CD4 + CD127 + CD25-) and Tregs (CD4 + CD127-CD25high).

The sorted CD4+ Teffs were stained according to the manufacturer with CellTrace™ Violet Cell Proliferation Kit (Thermo Fisher Scientific). 10 K labeled Teffs per well were seeded in a 96-well U-bottom plate. Tregs were added to the CD4+ Teffs in different ratios: 2:1, 1:1, 1:2, or 1:4. To induce proliferation, Dynabeads™ Human T-Activator CD3/CD28 (Gibco/ThermoFisher Scientific) was used in 1:20 ratio to Teffs. The cells were stained with anti–CD4-PE (#561841, BD Biosciences) monoclonal antibody to measure the proliferation after 6 days of incubation. The suppressive capacity of Tregs was calculated by comparing the division of Teffs in the presence of Tregs to the control wells without Tregs.

### Activation marker assay

PBMCs derived from healthy controls and patients were thawed and incubated for monocyte attachment for 4 h at 37°C in cell culture-treated T25 flasks (Corning) at 1.5 M cells/ml of complete RPMI media (#ECB9006L, Euroclone, Milan, Italy) with 10% FBS (Gibco/Thermo Fisher Scientific), 1x L-glutamine and Penicillin–Streptomycin solutions (#ECB3000 and #ECB3001, Euroclone). Suspension cells were then collected, counted, and plated at 120 000/well on non-treated U-bottom 96-well microplates (Corning). Plates were rested for 1 h at 37°C before addition of the following stimulants (final concentration indicated): 1 μg/ml PHA (#11249738001, Merck, Rahway, NJ, USA) or anti-CD3/CD28-coated beads (Dynabeads™ Human T-Activator CD3/CD28, #11131D, Gibco/Thermo Fisher Scientific) at 1:2 bead-to-cell ratio. All conditions were prepared in duplicate, and plates were incubated at 37°C for 16 or 40 h. For unstimulated samples, complete RPMI was added instead of a stimulation mix, and samples were incubated for 16 h. Cells were then moved to a 96-well V-bottom plate (Nunc #249935, Thermo Fisher Scientific), spun down for 6 min at 400 g, and resuspended in a staining mix containing FACS buffer (PBS with 2% FBS and 2 mM EDTA) and the following reagents and antibodies: eBioscience™ Fixable Viability Dye eFluor™ 660 (1:2800, #65–0864-14 Invitrogen/Thermo Fisher Scientific), CD4 BV421 (1:100, #562424), CD69 BV786 (1:280, #563834), CD25 PE-CF594 (1:80, #562403) and CD14 FITC (1:50, #345784) from BD Biosciences, and CD8 BV570 (1:50, #301038), CD3 APC-Fire (1:50, #300470) and CD19 AF488 (1:200, #302219) from Biolegend. Cells were stained for 30 minutes on ice, washed twice, and resuspended in FACS buffer before analysis on the IntelliCyt® iQue Screener PLUS (Violet-Blue-Red laser configuration) (Sartorius) flow cytometer. Antibody compensation samples were prepared with the UltraComp eBeads™ Compensation Beads (Invitrogen/ThermoFisher Scientific). Flow data was analyzed with FlowJo software (v10.8.1, BD Biosciences).

### T cell proliferation assay

PBMCs derived from healthy controls and patients were thawed and rested o/n at 37°C in non-treated T25 flasks (Corning) in complete RPMI media (described above). Next day, 5 million cells from each individual were stained in 1 μM Violet Proliferation Dye 450 (#562158, BD Biosciences) following manufacturer’s instructions. After staining, the cells were resuspended to complete RPMI at 1 million cells/ml and 100 000 cells/well were plated on U-bottom 96-well plates and rested in an incubator at 37°C for 3–4 h. Samples were then stimulated as described above for activation marker assay. Cells were incubated with the stimulants at 37°C for 4 days and then prepared for flow as described above using the following antibody panel: eBioscience™ Fixable Viability Dye eFluor™ 660 (1:3000), CD4 AF488 (1:120, #557695), CD8 PE-Cy7 (1:300, #335822) and CD3 BV605 (1:40, #563219) (all from BD Biosciences). Data was analyzed with the Proliferation tool on FlowJo (v10.8.1, BD Biosciences).

### Statistical analysis and data visualization

Flow-based and imaging-based data were processed, visualized, and the statistical differences were calculated with RStudio version 2022.07.0 based on R version 4.2.2. or R version 4.3.0 or RStudio version 2023.12.0 based on R version 4.3.2 or RStudio version 2023.12.1 based on R version 4.3.3 or RStudio version 2024.12.0 based on R version 4.4.2. Wilcoxon matched-pairs signed-rank test was used to compare the relative intensities of western blotting data. The AUC values obtained from the proliferation and wound-healing assays were compared with Welch’s t-test or Mann–Whitney U test. The statistical significance of immunofluorescence data was calculated with Welch’s t-test. The optimal plotting space for outliers was arranged with ggbreak v0.1.0. package [60].

## Supplementary Material

Supplementary_Figures_ddaf081

Supplementary_Material_ddaf081
