# Supplementary material for: Genome sequencing reveals CCDC88A variants in malformations of cortical development and immune dysfunction
Source: Hum Mol Genet. 2025 May 22;34(15):1294–312. doi: 10.1093/hmg/ddaf081 (PMC12278729; doi:10.1093/hmg/ddaf081)
Supplement: Supplementary_Figures_ddaf081 [file supplementary_figures_ddaf081.pdf]

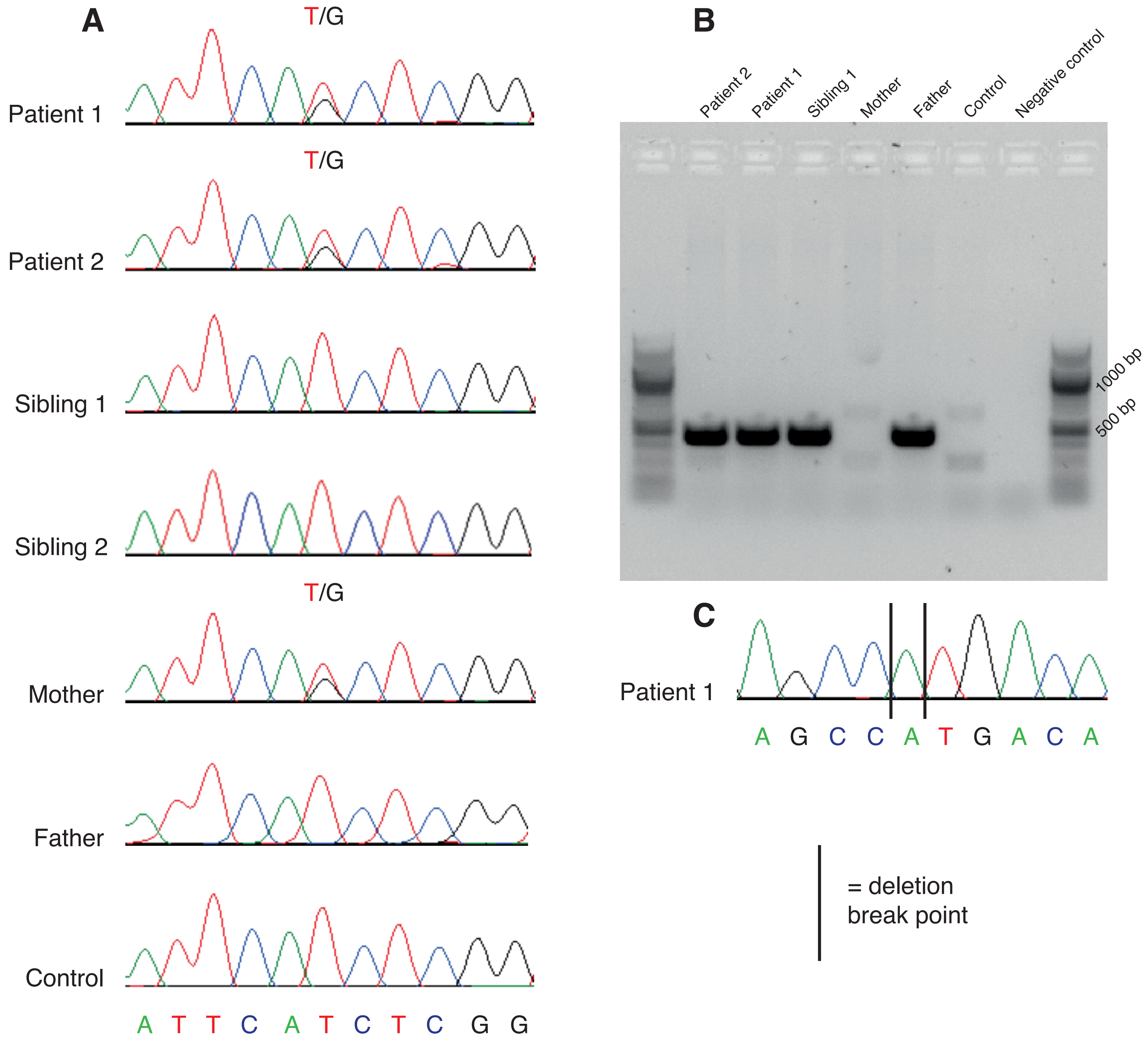

Figure S1. Variant verifications. (A) Capillary sequencing chromatograms verified that the affected children and the mother harbor the c.929A>C variant in exon 10 of CCDC88A (NM_001135597.1). (B) An agarose gel image of PCR fragments with the deletion of exons 14–16. The primers were designed near the predicted break points at both ends flanking the deletion. The PCR reaction was successful only for those samples that carried the deletion. The sample of sibling 2 was run subsequently and it showed no deletion (data not shown). (C) Capillary sequencing chromatograms determined the exact size (9796 bp) of the CCDC88A deletion, NC_000002.11:g.55556194_55565990del. Both deletion breakpoints ended with adenines and only one of them is included in the deletion, as such, the breakpoints can only be determined with one nucleotide accuracy. The sequencing electropherograms are in the reverse strand.

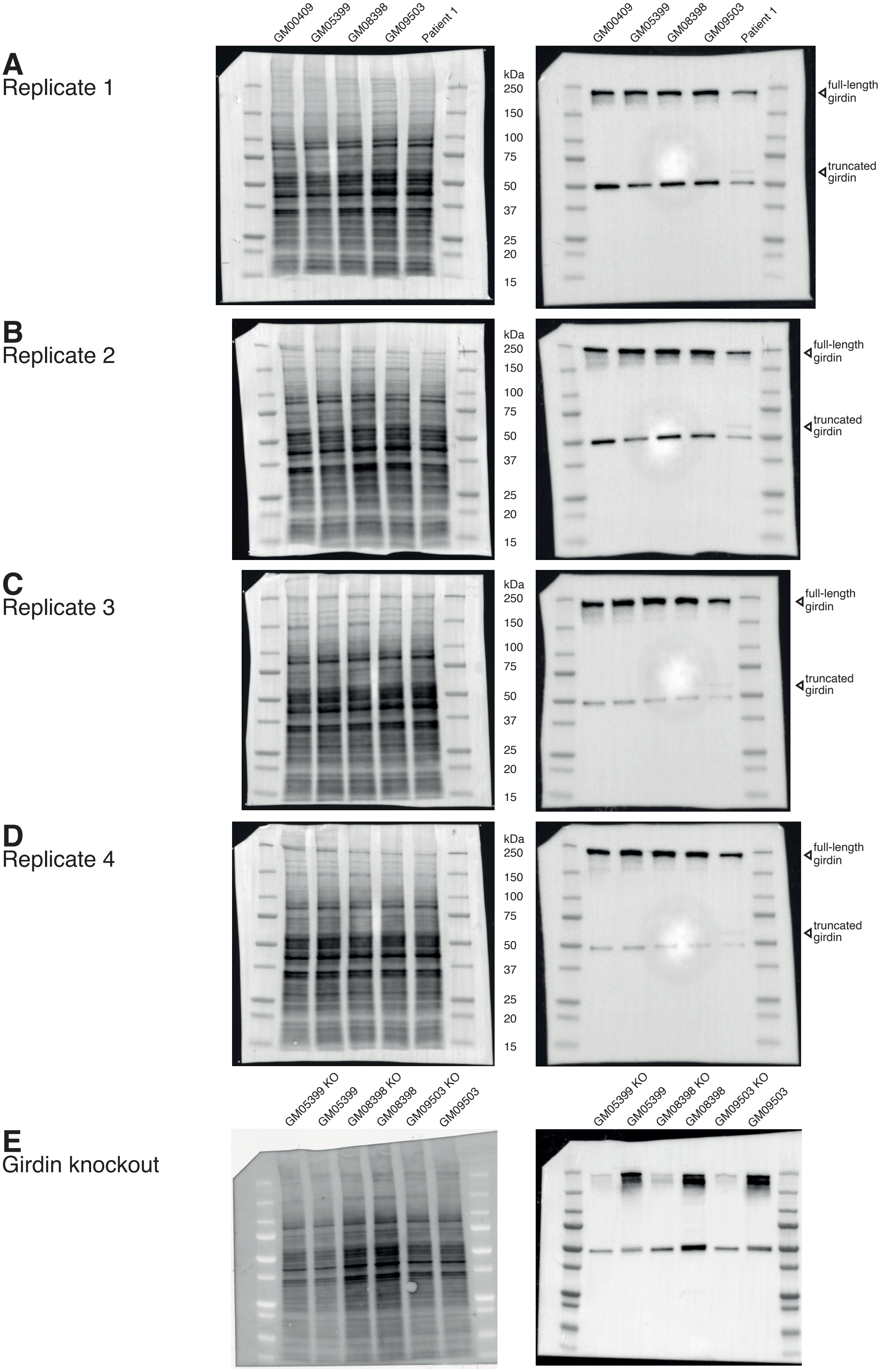

Figure S2. Original Western blot images that were used in the quantification. Total protein stainings used in the total protein normalization are on the left side. ECL reaction images are on the right side. (A-D) The same pipetting order was used on all blots in different replicates. (E) Girdin knockout experiment blot.

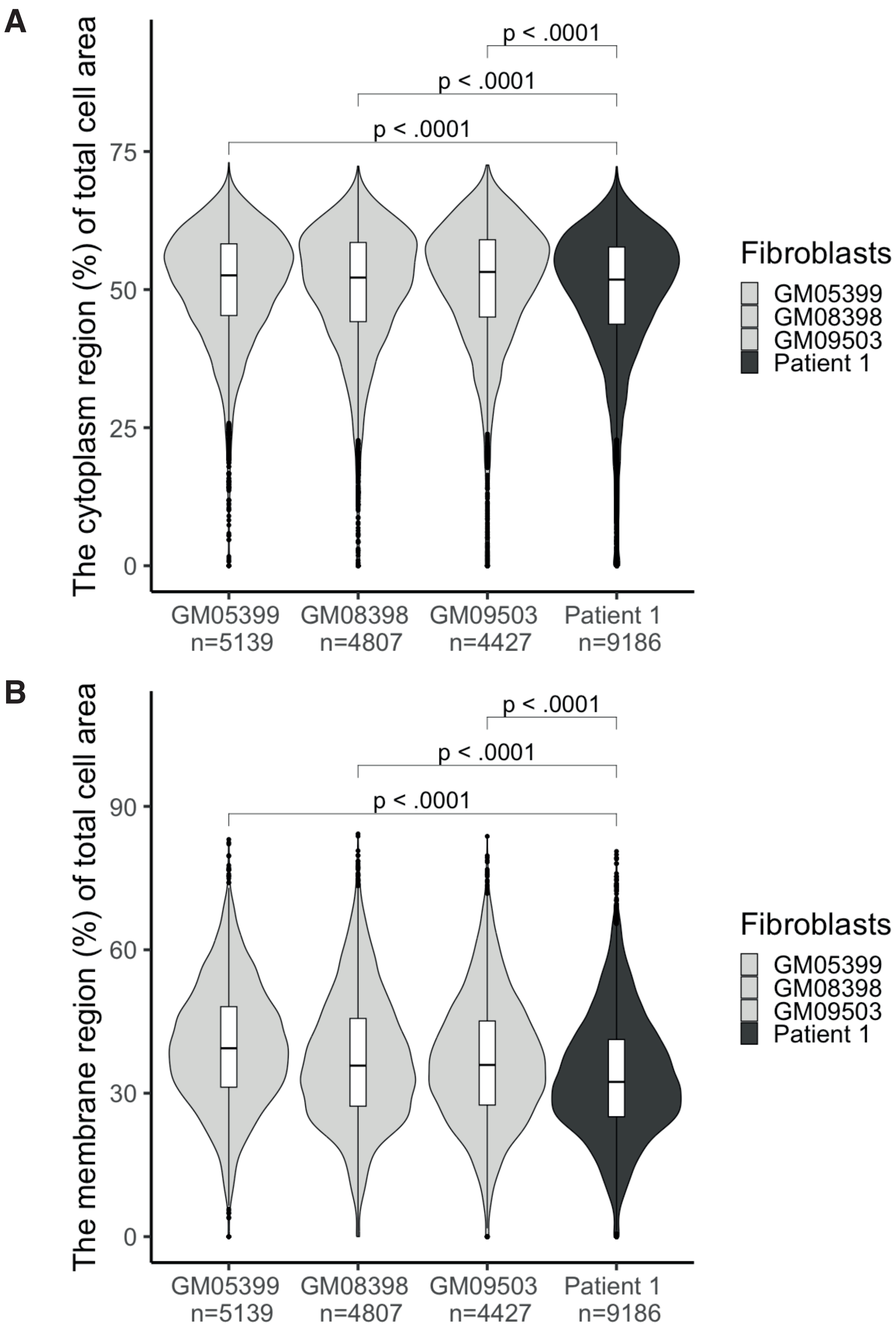

Figure S3. The cytoplasm and cell membrane regions are smaller in size Patient 1 fibroblasts compared with controls. Violin plots visualize how many percentages (A) the cytoplasm and (B) the membrane regions are of the total cell area. No EGF stimulation, data are pooled from three different technical replicates, unpaired t-test, horizontal lines of boxplots represent median values, and n = cell number.

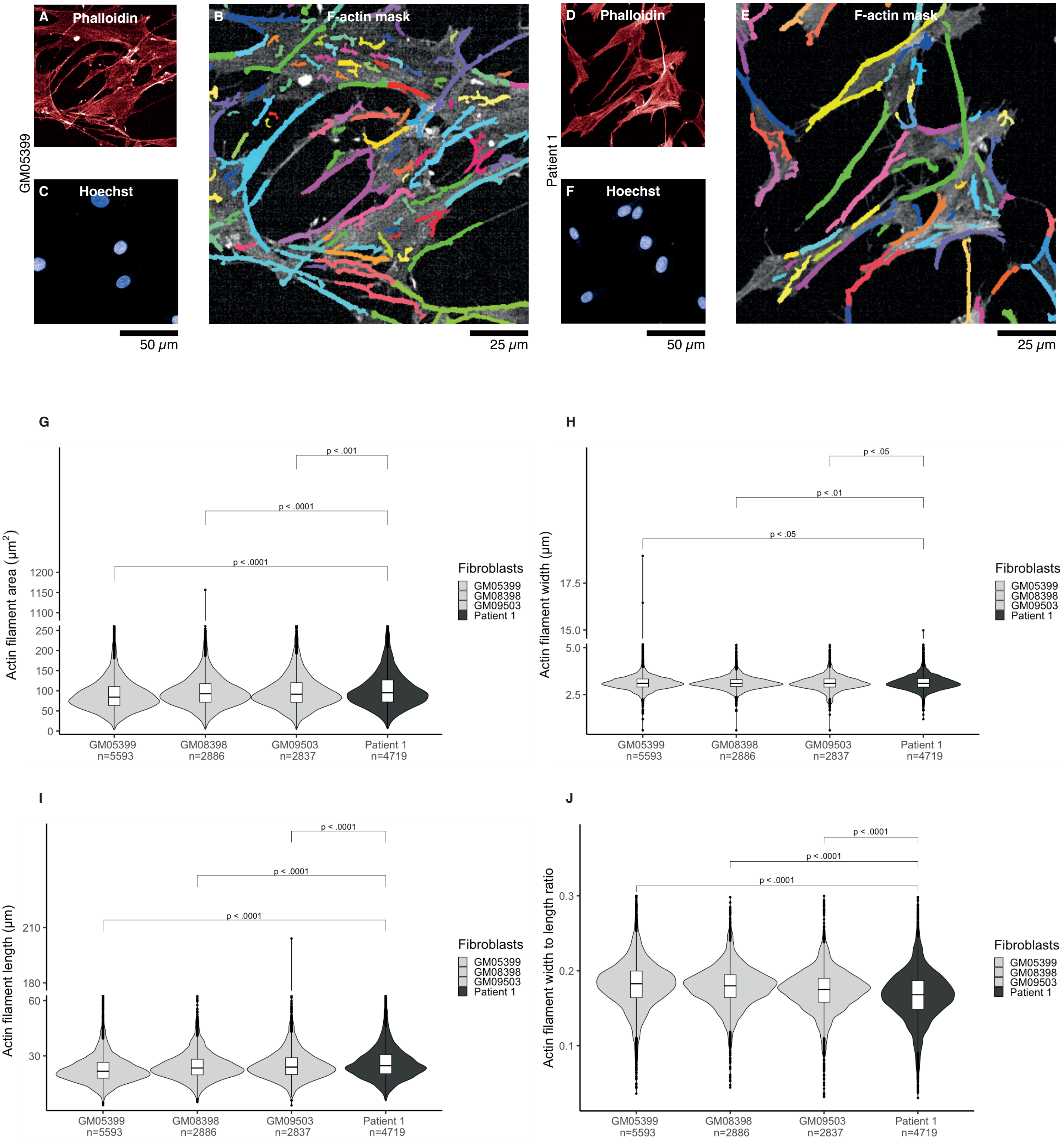

Figure S4. Actin filament structures are altered in Patient 1 fibroblasts. Exemplary images illustrate actin staining, an algorithm-based mask for filamentous actin (F-actin), and nucleus staining (A-C) GM05399 (D-F) Patient 1. Violin plots represent that (G) actin filaments take a bigger area, (H) are wider, and (I) are longer in Patient 1 fibroblasts compared with control fibroblasts. (J) The actin filament width-to-length ratio is smaller in Patient 1 fibroblasts than in controls which indicates that actin filaments in patient fibroblasts are especially lengthened rather than widened. In general, the actin filament structures of Patient 1 fibroblasts have more variation than the structures in controls (the violin shapes of the Patient 1 sample are not as wide as the violins of control samples). No EGF stimulation, data are from one technical replicate, unpaired t-test, horizontal lines of boxplots represent median values, and n = cell number.

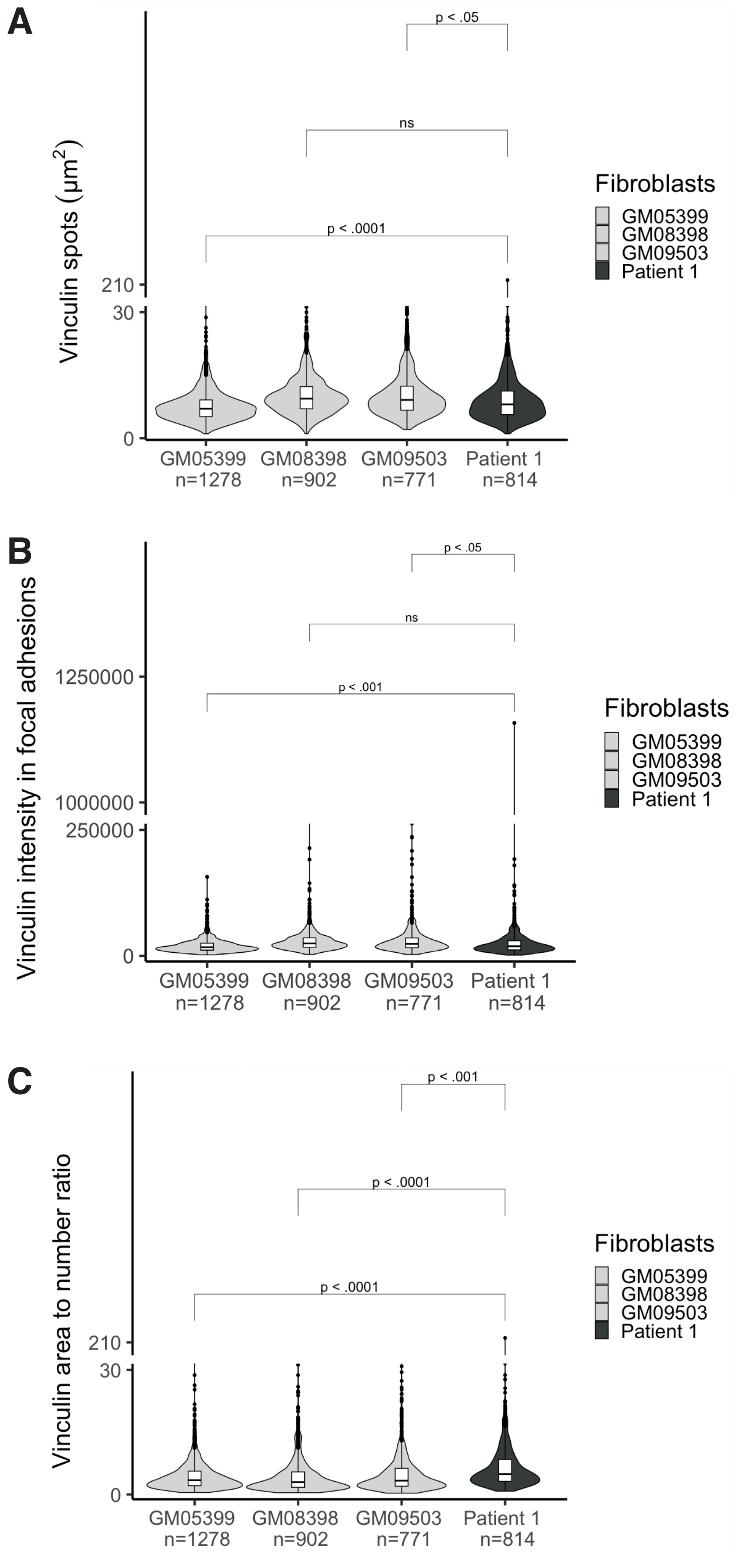

Figure S5. Vinculin analysis. Vinculin staining showed that (A) the spot size and (B) intensity vary in different fibroblasts used in the study. (C) Vinculin area-to-number ratio was increased in Patient 1 fibroblasts compared with controls. No EGF stimulation, data are from one technical replicate, unpaired t-test, horizontal lines of boxplots represent median values, and n = cell number.

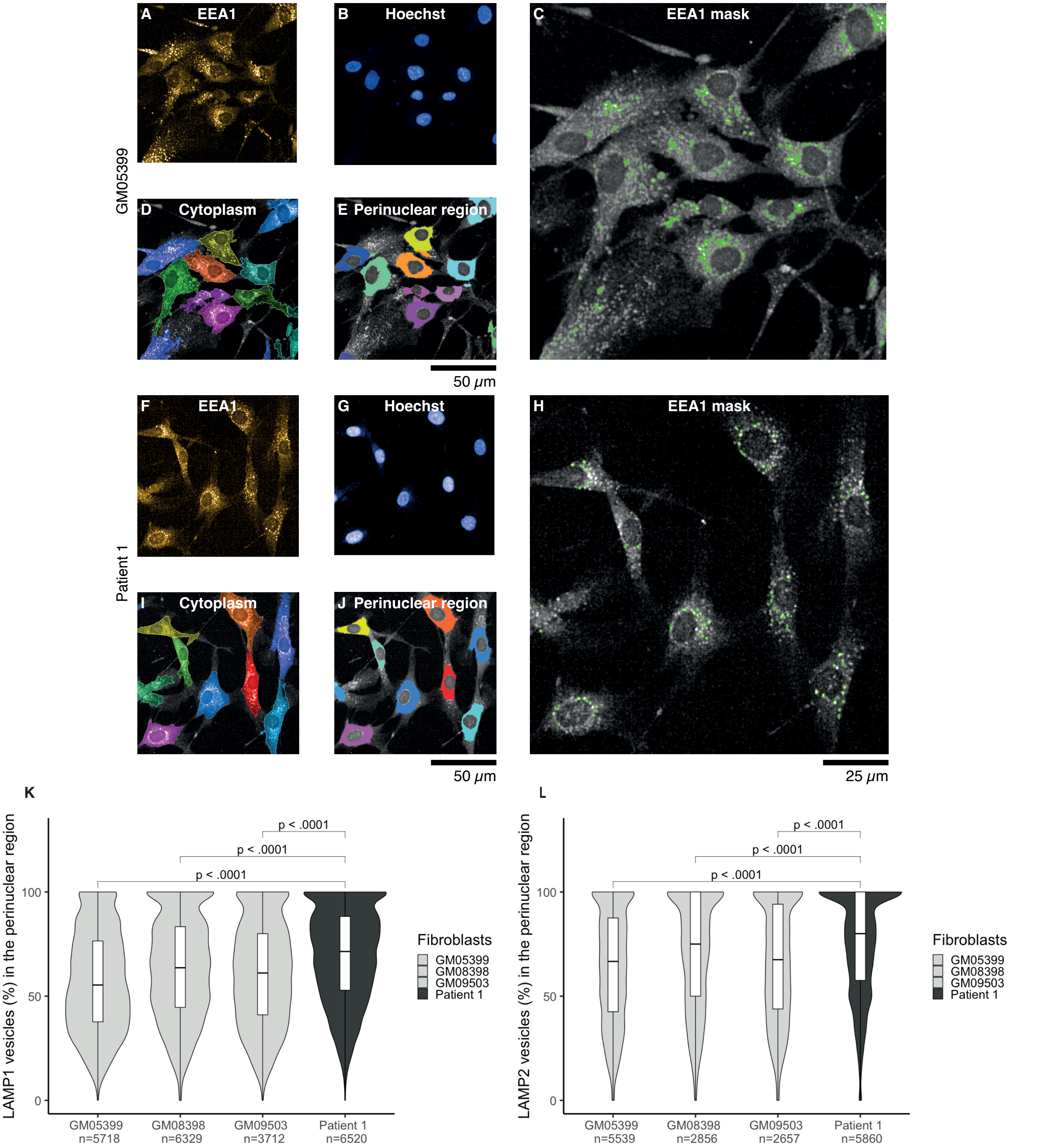

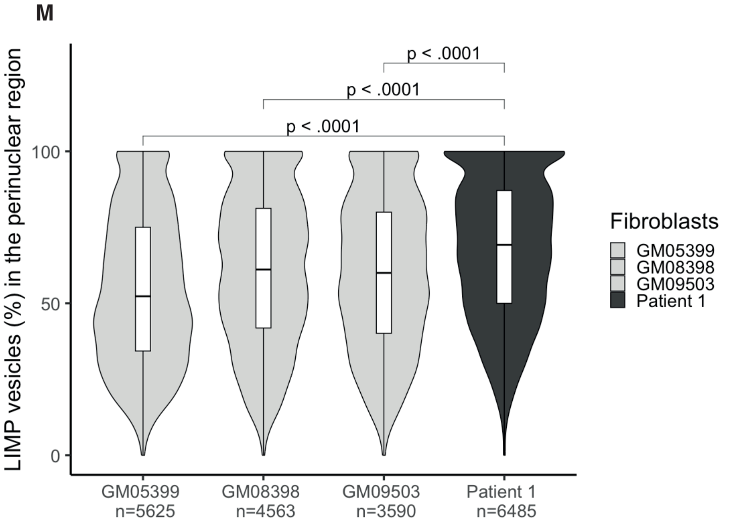

Figure S6. The vesicles of the autophagy-lysosomal pathway accumulate more in the perinuclear region of Patient 1 fibroblasts than in controls. Exemplary images illustrate early endosomes (EEA1) staining, nucleus staining, EEA1 algorithm-based mask, cytoplasm, and perinuclear region quantifications (A-E) GM05399 (F-J) Patient 1. Accumulation occurs for (K) lysosome-associated membrane proteins I (LAMP1), (L) lysosome-associated membrane proteins II (LAMP2), and (M) lysosomal glycoprotein III (LIMP) vesicles. No EGF stimulation, data are pooled from three different technical replicates, unpaired t-test, horizontal lines of boxplots represent median values, and n = cell number.

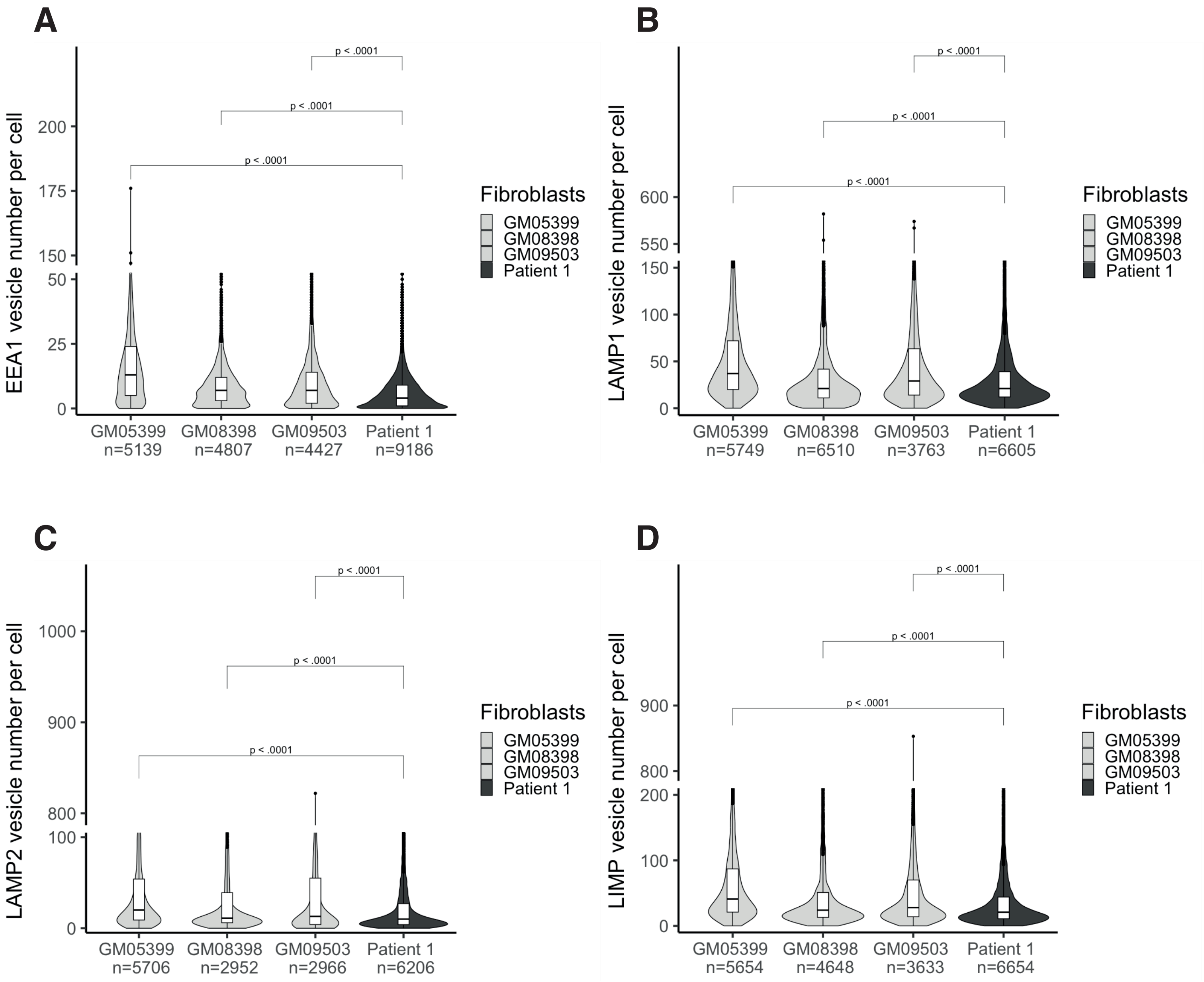

Figure S7. Patient 1 fibroblasts have fewer autophagy-lysosomal pathway vesicles than controls. The violin plots show the reduced vesicle numbers of (A) early endosomes (EEA1), (B) lysosome-associated membrane proteins I (LAMP1), (C) lysosome-associated membrane proteins II (LAMP2), and (D) lysosomal glycoprotein III (LIMP). Data are pooled from three different technical replicates, unpaired t-test, horizontal lines of boxplots represent median values, and n = cell number.

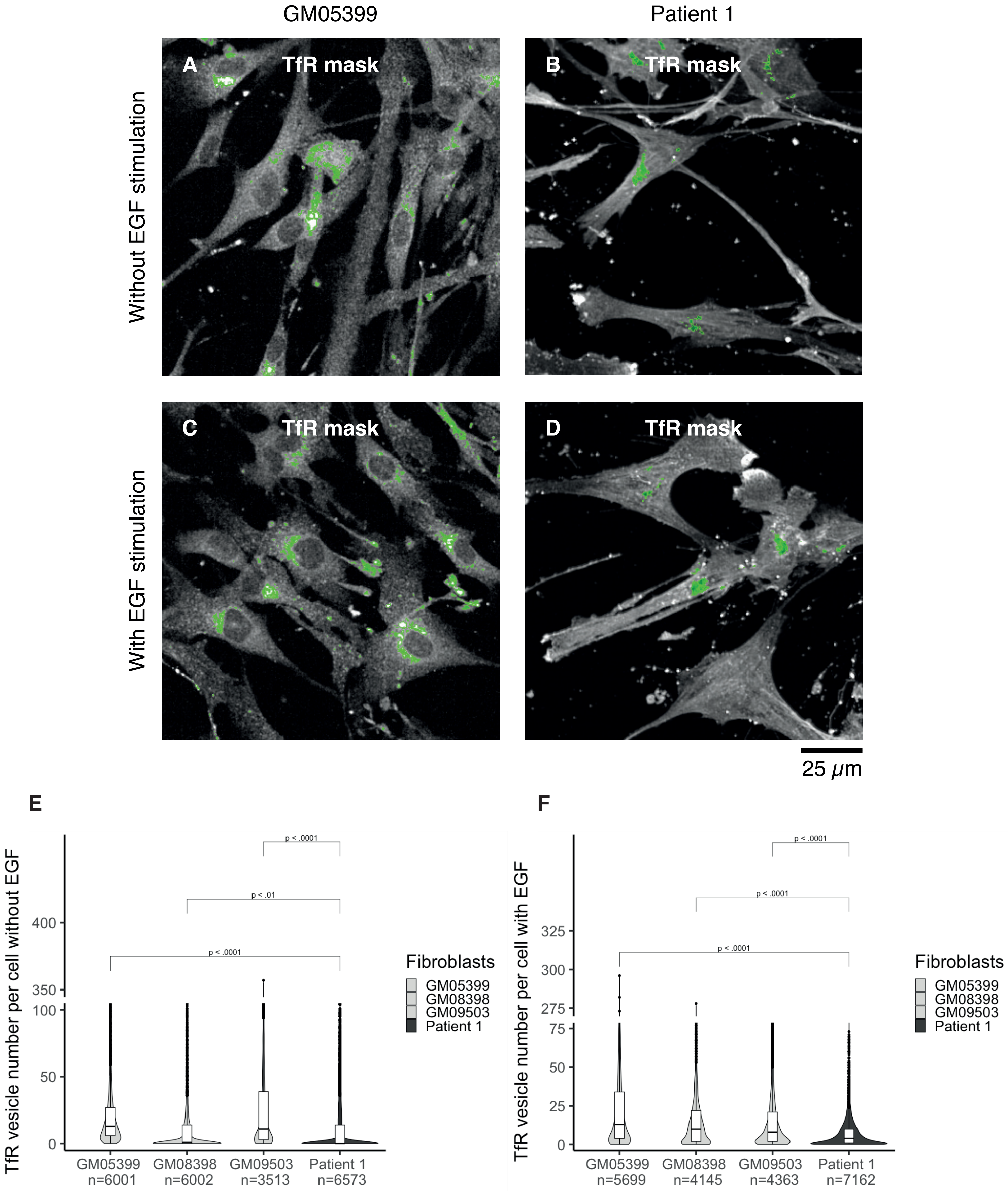

Figure S8. The number of transferrin receptors (TfR) is reduced in the Patient 1 fibroblasts compared with controls. Exemplary images show algorithm-based TfR mask without EGF stimulation (A) GM05399 (B) Patient 1 and TfR mask with EGF stimulation (C) GM05399 (D) Patient 1. Violin plots show that Patient 1 fibroblasts had the least TfR (E) without EGF stimulation and (F) with EGF stimulation. GM05399 was selected to represent controls in the images, data are pooled from three different technical replicates, unpaired t-test, horizontal lines of boxplots represent median values, and n = cell number.

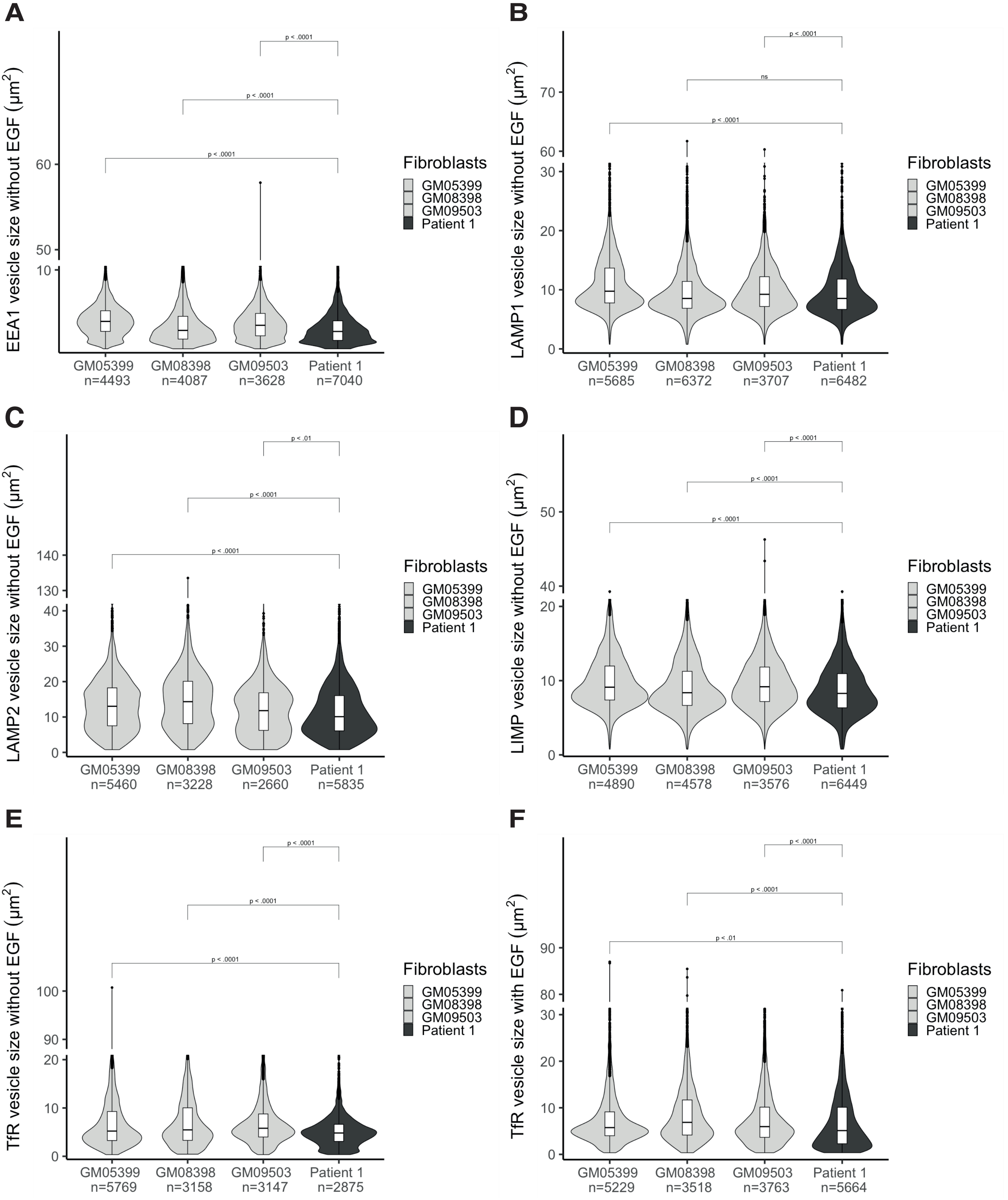

Figure S9. The autophagy-lysosomal pathway vesicles were mainly smaller in Patient 1 fibroblasts compared with controls. The violin plots show the reduced vesicle size of (A) early endosomes (EEA1), (B) lysosome-associated membrane proteins I (LAMP1) except no size difference in the LAMP1 vesicles between P1 and GM08398, (C) lysosome-associated membrane proteins II (LAMP2), (D) lysosomal glycoprotein III (LIMP), (E) TfR without EGF, and (F) TfR with EGF. Data are pooled from three different technical replicates, unpaired t-test, horizontal lines of boxplots represent median values, and n = cell number.

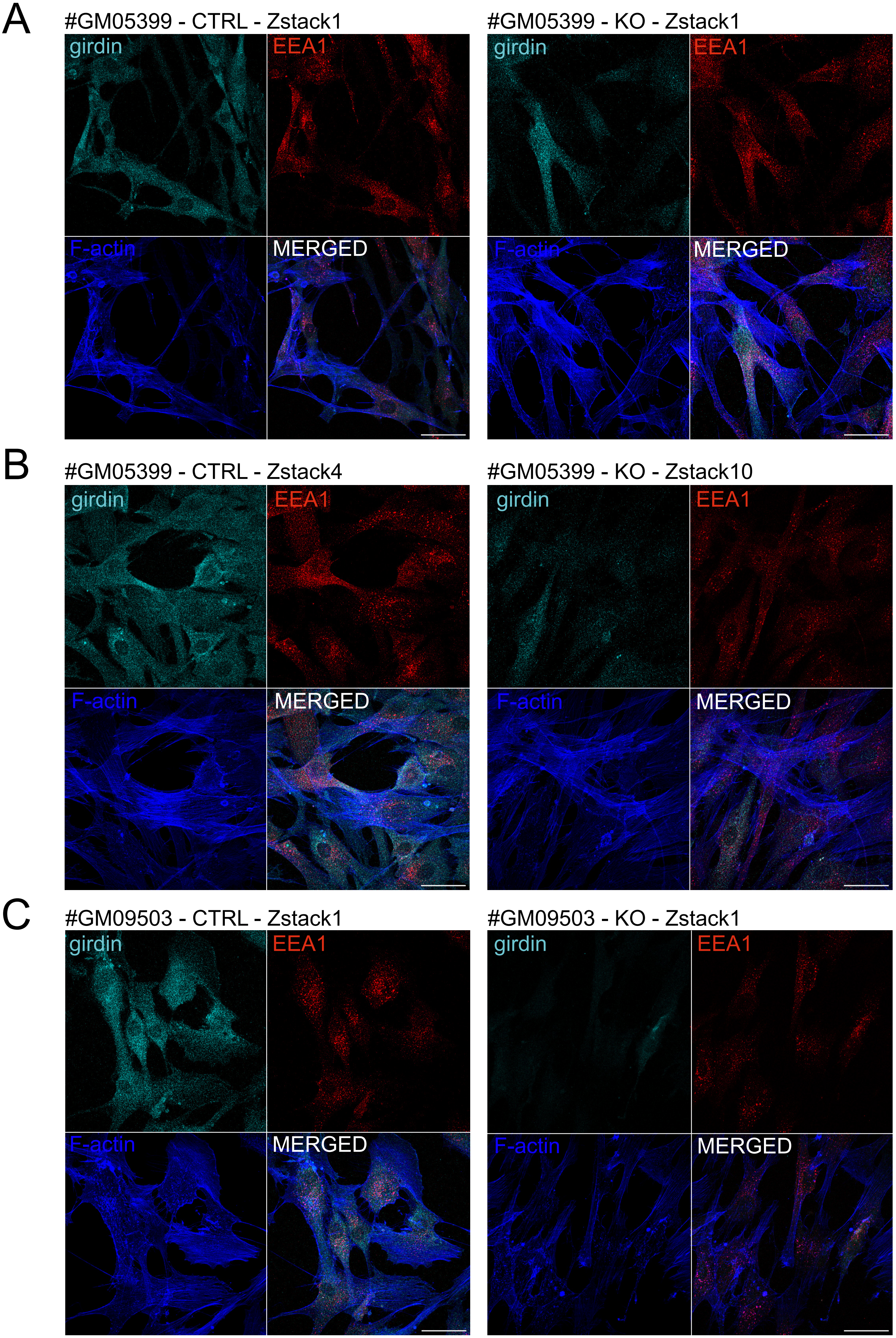
A
#GM05399 - CTRL - Zstack1
girdin
EEA1
F-actin
MERGED
#GM05399 - KO - Zstack1
girdin
EEA1
F-actin
MERGED
B
#GM05399 - CTRL - Zstack4
girdin
EEA1
F-actin
MERGED
#GM05399 - KO - Zstack10
girdin
EEA1
F-actin
MERGED
C
#GM09503 - CTRL - Zstack1
girdin
EEA1
F-actin
MERGED
#GM09503 - KO - Zstack1
girdin
EEA1
F-actin
MERGED

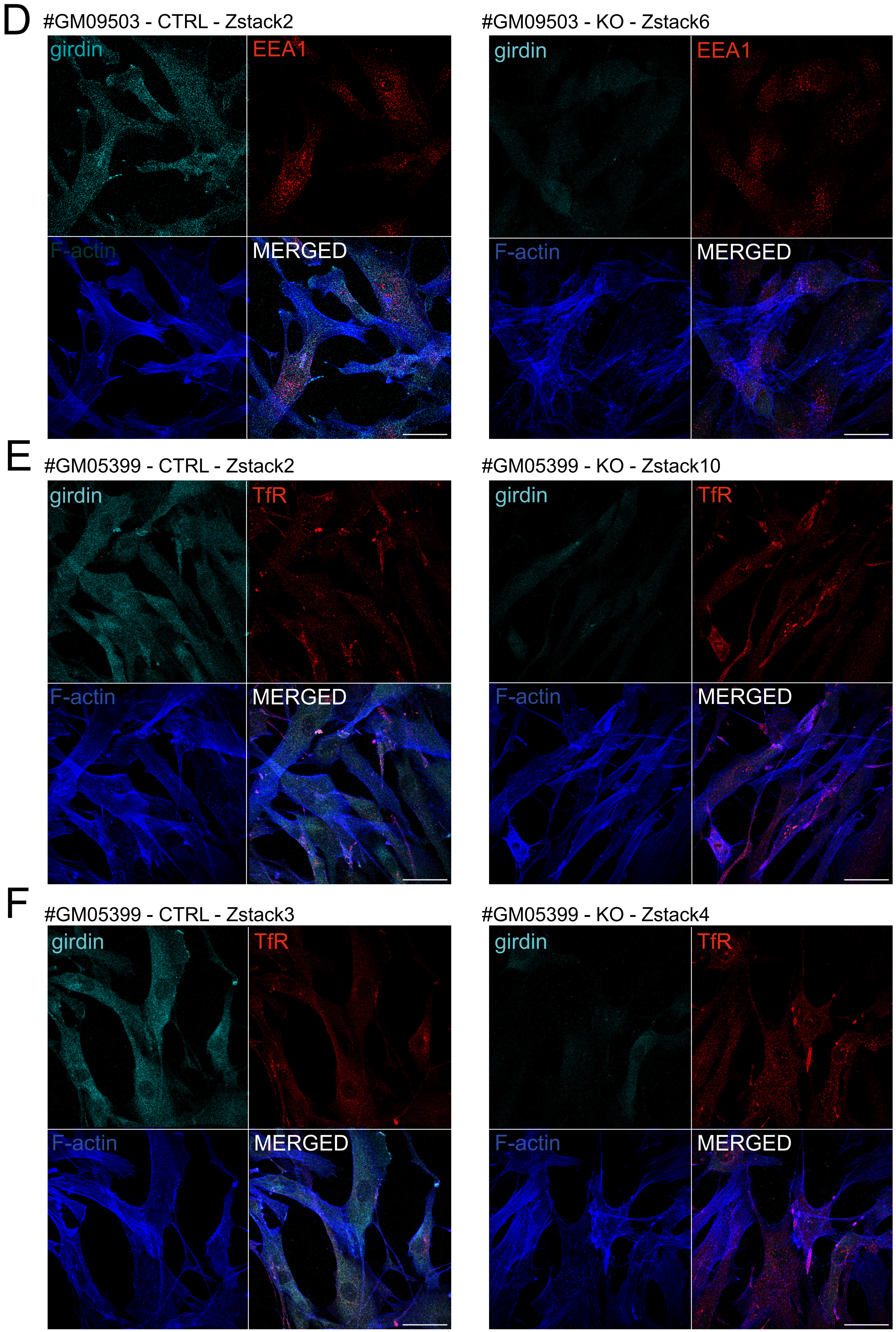
D
#GM09503 - CTRL - Zstack2
girdin
EEA1
F-actin
MERGED
#GM09503 - KO - Zstack6
girdin
EEA1
F-actin
MERGED
E
#GM05399 - CTRL - Zstack2
girdin
TfR
F-actin
MERGED
#GM05399 - KO - Zstack10
girdin
TfR
F-actin
MERGED
F
#GM05399 - CTRL - Zstack3
girdin
TfR
F-actin
MERGED
#GM05399 - KO - Zstack4
girdin
TfR
F-actin
MERGED

G #GM09503 - CTRL - Zstack4 #GM09503 - KO- Zstack1

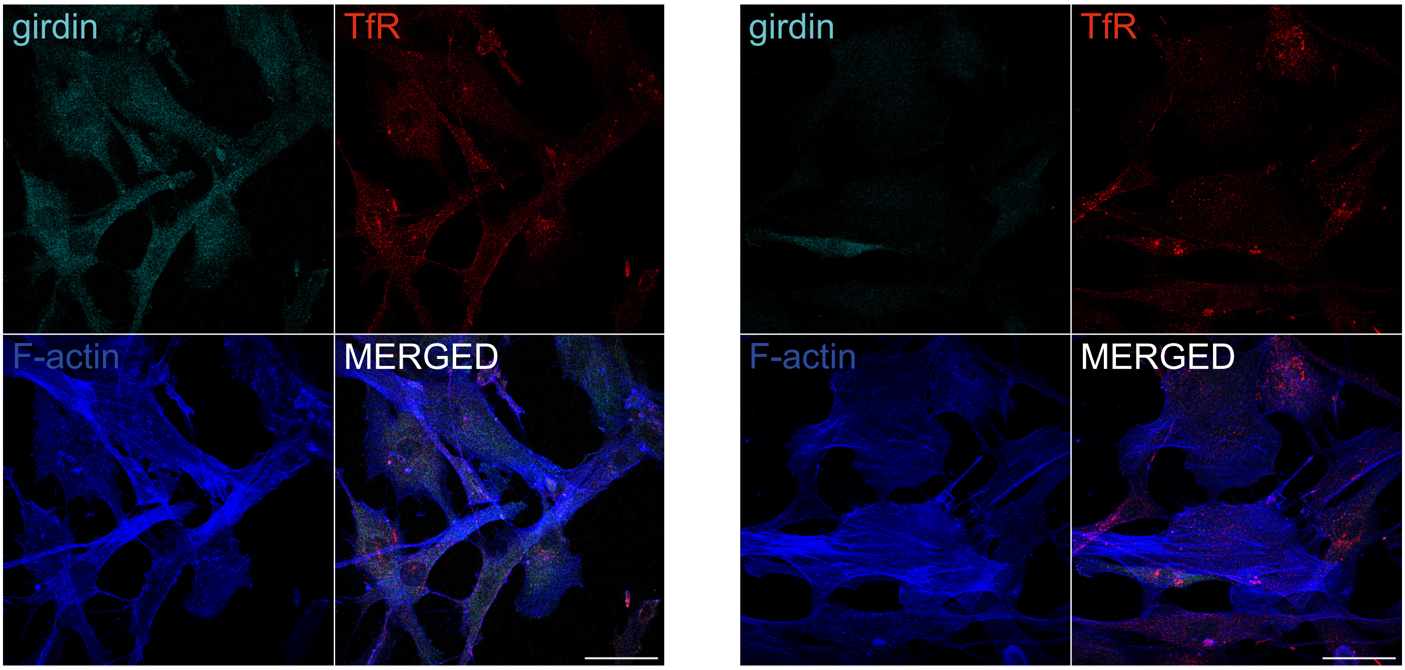

H #GM09503 - CTRL - Zstack5 #GM09503 - KO- Zstack4

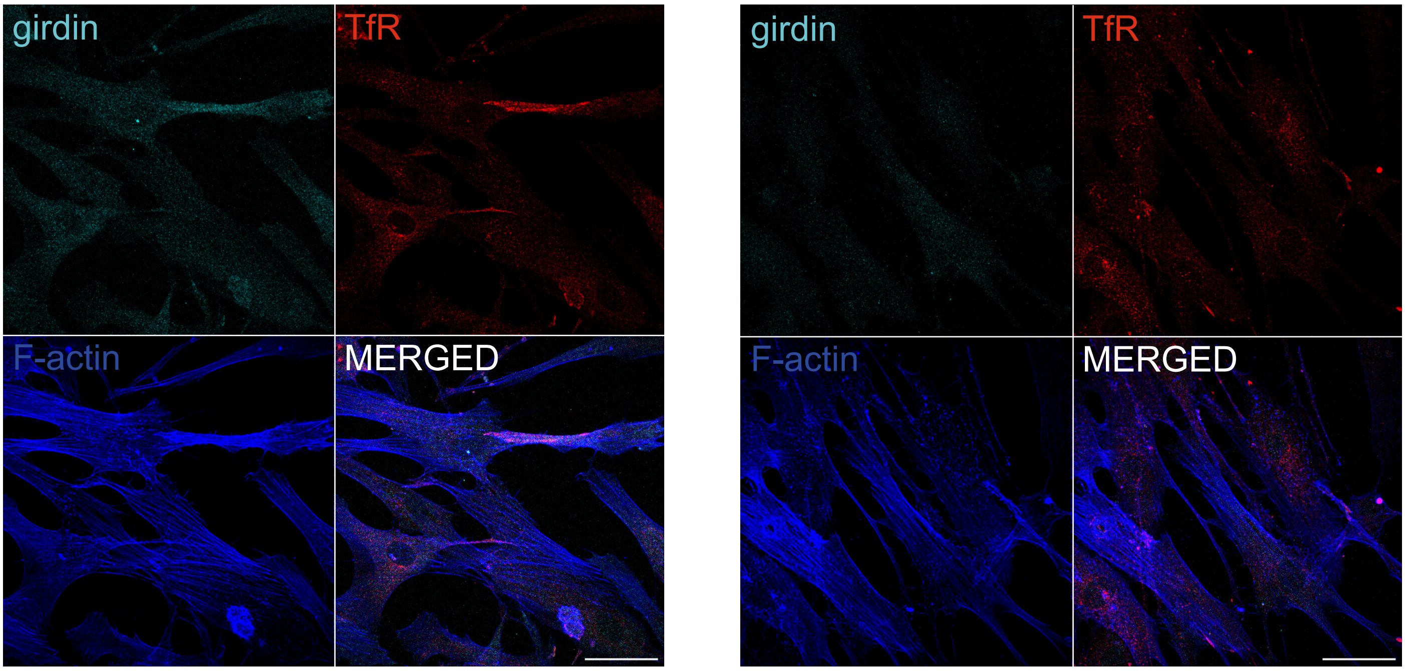

Figure S10. Representative confocal microscopy images of immunostainings in control and girdin-knockout fibroblasts. Girdin expression is shown in cyan, early endosomes in red (A-D), recycling endosomes in red (E-H), and the actin cytoskeleton in blue. Images are from GM05933 fibroblasts (A-B, E-F) and GM09503 fibroblasts (C-D, G-H). Scale bar = 50 µm. Maximum intensity projections of 4 µm Z-stacks were consistently background-subtracted across all images.

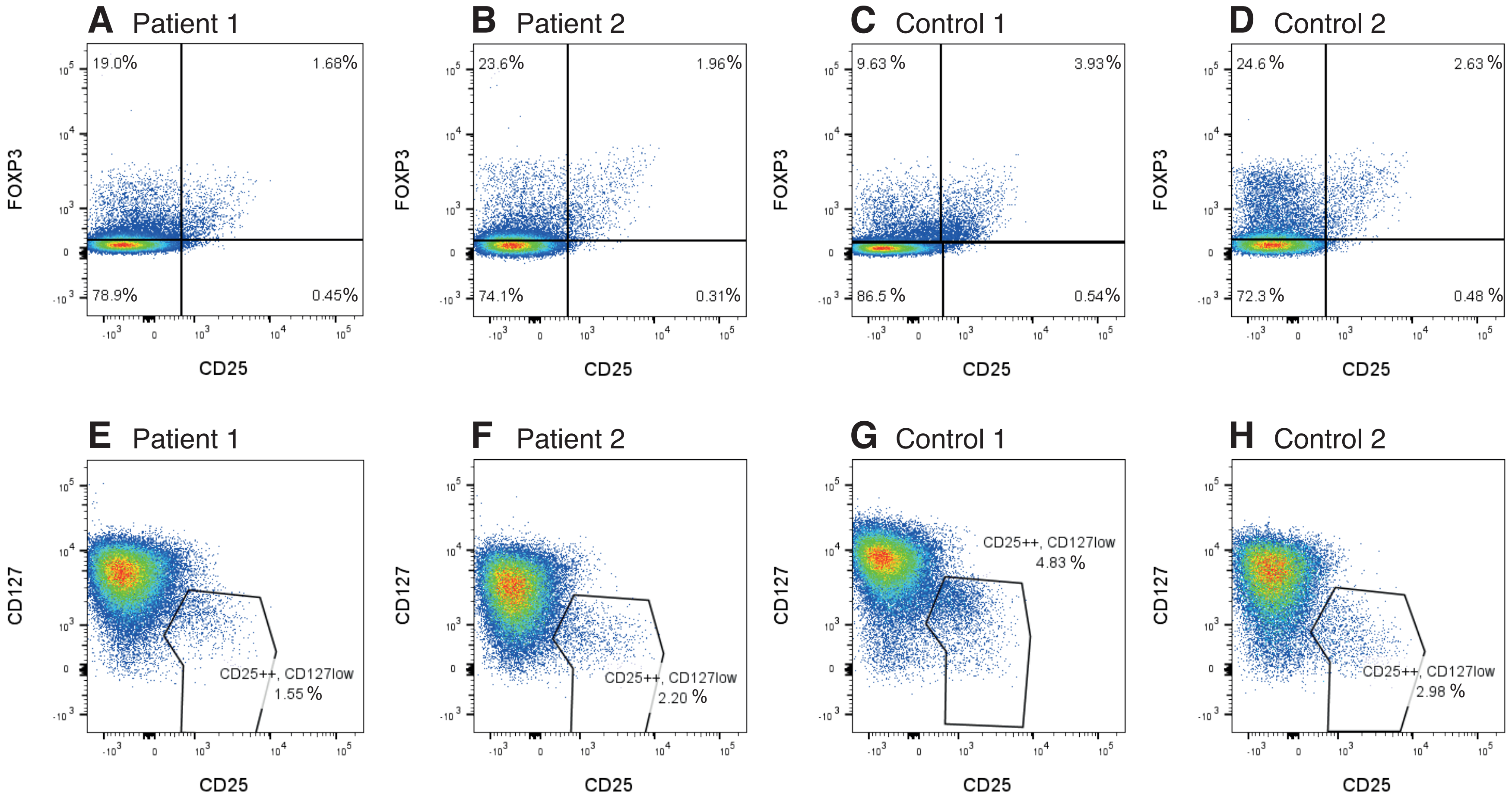

Figure S11. Flow-based immune cell phenotyping. (A-D) FOXP3+CD25highCD4+ analysis revealed a lower percentage of regulatory T cells (Tregs) in patient samples (1.68% and 1.96%) than in controls (3.93% and 2.63%). (E-H) The reduced Treg percentage was also observed using CD127low/negCD25highCD4+ staining in patient samples (1.55% and 2.20%) compared with controls (4.83% and 2.98%).

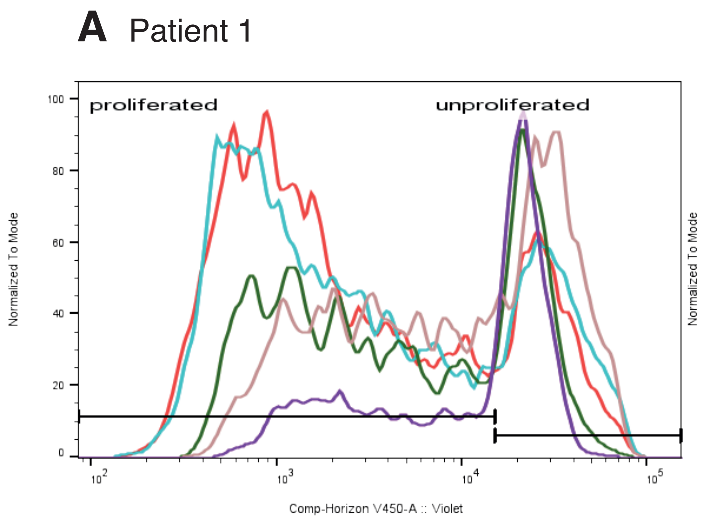

	Sample Name	Subset Name	Count
	QU 56 Hki_Teff1_Treg2_B_3_H03.fcs	proliferation	6494
	QU 56 Hki_Teff1_Treg1_B_2_G02.fcs	proliferation	9945
	QU 56 Hki_Teff2_Treg1_B_2_F02.fcs	proliferation	11527
	QU 56 Hki_Teff4_Treg1_B_2_E02.fcs	proliferation	12691
	QU 56 Hki_Teff_B_1_B01.fcs	proliferation	13455

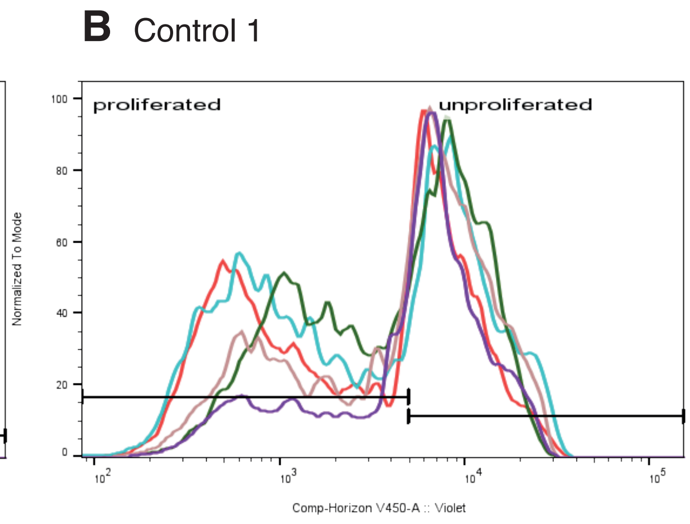

	Sample Name	Subset Name	Count
	kEK_Teff1_Treg2_B_2_H11.fcs	proliferation	7187
	kEK_Teff1_Treg1_B_3_G12.fcs	proliferation	9579
	kEK_Teff2_Treg1_B_2_F11.fcs	proliferation	7319
	kEK_Teff4_Treg1_B_3_E12.fcs	proliferation	6362
	kEK_Teff_B_2_B11.fcs	proliferation	8158

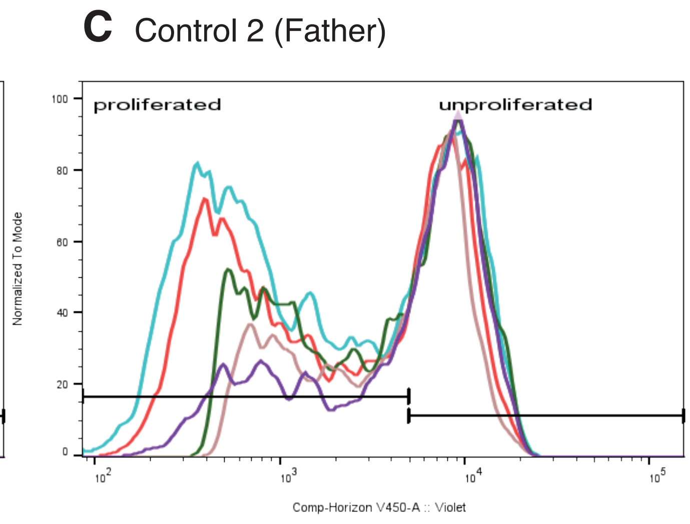

	Sample Name	Subset Name	Count
	Matkak isa Hki_Teff1_Treg2_B_2_H05.fcs	proliferation	7834
	Matkak isa Hki_Teff1_Treg1_B_3_G06.fcs	proliferation	6106
	Matkak isa Hki_Teff2_Treg1_B_3_F06.fcs	proliferation	8343
	Matkak isa Hki_Teff4_Treg1_B_2_E05.fcs	proliferation	10945
	Matkak isa Hki_Teff_B_1_B04.fcs	proliferation	9549

Figure S12. Treg suppression assay for CD4+CD127+CD25- effector T (Teff) cells upon Dynabeads T-Activator CD3/CD28 stimulation. (A) No significant changes were determined in the suppression efficiency of Patient 1 Tregs compared with control samples (B-C). Treg suppression assay for Patient 2 failed.

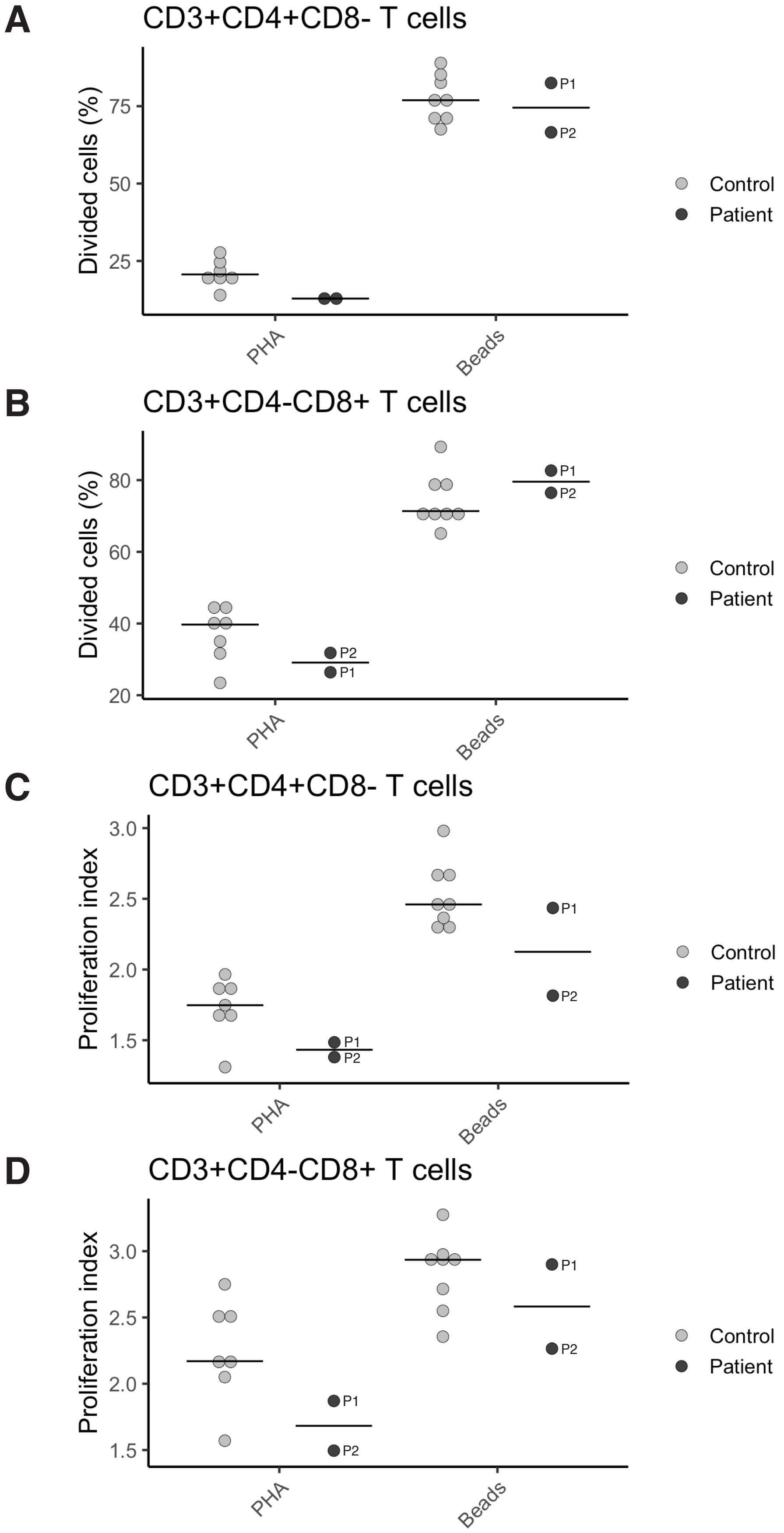

Figure S13. T cell proliferation responses of girdin-deficient patients. Patient and healthy control-derived PBMCs were stimulated for 4 days with PHA or anti-CD3/CD28-coated beads and proliferation was quantified with a flow-based assay. Data were analyzed with the Proliferation tool on FlowJo. Percentage of original (A) CD4 and (B) CD8 T cells that started dividing in response to the indicated stimulations. The proliferation index indicates the average number of divisions per dividing cell for (C) CD4 and (D) CD8 T cells. The results indicate that girdin deficiency may reduce the speed or robustness of the T cell proliferative response but it does not prevent initiation of proliferation. Horizontal lines in the plots represent median values.

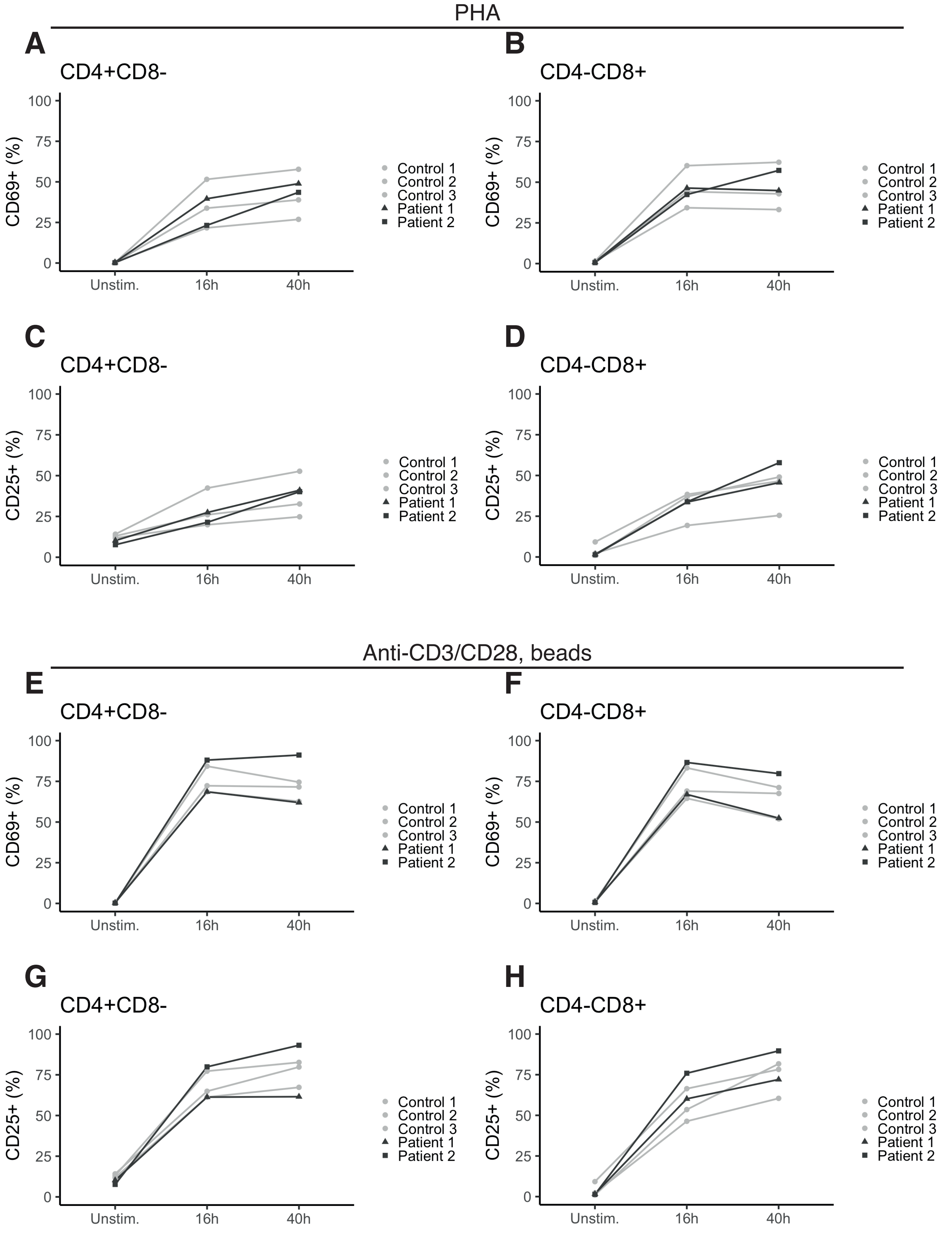

Figure S14. T cell activation marker assay. Upregulation of CD69 and CD25 in CD14-CD19-CD4+CD8- and CD14-CD19-CD4-CD8+ T cells were assayed at 16 or 40 hours after stimulation. Unstimulated cells were analyzed at a 16h time point. (A-D) PHA and (E-H) anti-CD3/CD28-coated bead stimulation. The assay did not show any consistent differences between patient and control samples.

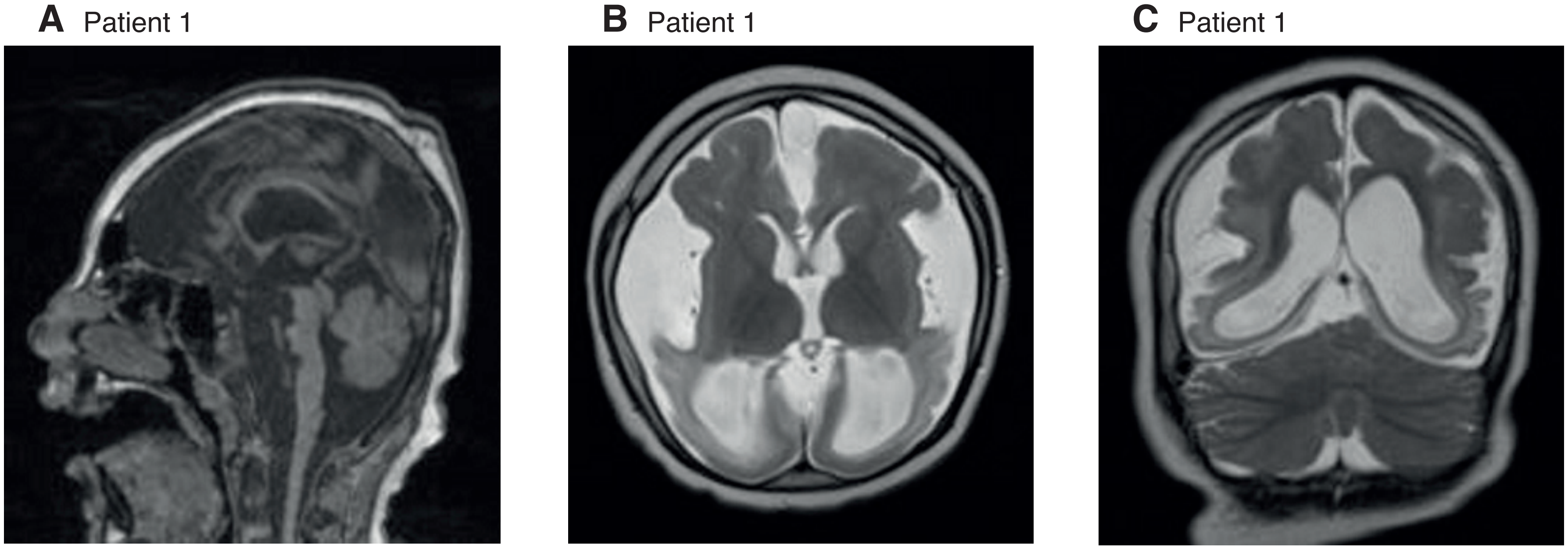

Figure S15. A repeated brain MRI. (A) T1 weighted image shows hypoplastic corpus callosum and pons. Vermis is normal. (B-C) T2 weighted axial and coronal images show temporo-parietal gliotic changes with unknown etiology. Subarachnoid space is enlarged because of atrophy. Cerebellar hemispheres are of normal size. The brain MRI of Patient 1 was repeated at 7 years 11 months of age.
