# Supplementary material for: Genome sequencing reveals CCDC88A variants in malformations of cortical development and immune dysfunction
Source: Hum Mol Genet. 2025 May 22;34(15):1294–312. doi: 10.1093/hmg/ddaf081 (PMC12278729; doi:10.1093/hmg/ddaf081)
Supplement: Supplementary_Material_ddaf081 [file supplementary_material_ddaf081.pdf]

Supplementary Material to

Genome sequencing reveals *CCDC88A* variants in malformations of cortical development and immune dysfunction

Authors and affiliations:

Johanna Lehtonen[1,2,3,4], Anna H Hakonen[5], Antti Hassinen[2], Sanne Iversen Lurås[1], Meri Kaustio[2], Virpi Glumoff[6], Francisca Hinrichsen[1], Weiwei Li[1], Anna-Maija Sulonen[2], Sanna Wickman[7], Henrikki Almusa[2], Minttu Polso[2], Maarit Palomäki[8], Sirpa Kivirikko[5], Kristiina Avela[5,9], Kaarina Heiskanen[10], Vilja Pietiäinen[2], Kristiina Aittomäki[11], Janna Saarela[1,2,4,5,*]

1) Centre for Molecular Medicine Norway (NCMM), University of Oslo, Oslo, Norway
2) Institute for Molecular Medicine Finland (FIMM), HiLIFE, University of Helsinki, Helsinki, Finland
3) Folkhälsan Research Center, Helsinki, Finland
4) Department of Medical Genetics, Oslo University Hospital, Oslo, Norway
5) Department of Clinical Genetics, HUSLAB, HUS Diagnostic Center, Helsinki University Hospital and University of Helsinki, Helsinki, Finland
6) Medical Research Laboratory Unit, Faculty of Medicine, University of Oulu, Oulu, Finland
7) Department of Pediatric Neurology, Hyvinkää Hospital, Helsinki and Uusimaa Hospital District, Hyvinkää, Finland
8) Department of Radiology, Helsinki University Hospital, Helsinki, Finland
9) Turku University Hospital, University of Turku, Turku, Finland
10) New Children's Hospital, HUS, Helsinki University Hospital and University of Helsinki, Helsinki, Finland
11) Department of Medical and Clinical Genetics, University of Helsinki, Helsinki, Finland

Patient descriptions

The patients were two siblings born at term to non-consanguineous Finnish parents. The parents had attended a mainstream school with curriculum adjustments due to their learning problems. In both pregnancies, fetal ventriculomegaly was noted at >30 gestational weeks, even though the ventricles had been normal in the second-trimester morphological ultrasound. The morphological ultrasound also showed a collection of fluid (8mm) in the abdominal region of patient 1 (P1) and the renal pelvises of patient 2 (P2) appeared dilated later during the pregnancy. Both patients had perinatal cardiac rhythm abnormalities (atrial flutter or supraventricular tachycardia), but no major cardiac problems thereafter (only secundum atrial septal defect in P1).

The brain magnetic resonance imaging (MRI) findings and the electroencephalograms (EEG) of both patients were clearly abnormal already during the first week of life (see details below). Epileptic medication (vigabatrin) of P1 was started at 7 months of age when he had recurrent paroxysmal symptoms (lip smacking, head and eye deviation, rhythmic laughter). P2 developed epileptic symptoms already during the first 24 hours of life (yawning, lip smacking, apneas, associated opisthotonus, as well as vomiting) and antiepileptic medication was commenced. In childhood, both patients continued having recurrent epileptic seizures several times per week despite polytherapy with three antiepileptic drugs (latest combination: valproate, levetiracetam, and lamotrigine), but most of the seizures were rather short-lived seizures not requiring hospitalization.

Both patients were hypotonic from infantile age and developed spasticity around the age of ~1 year (1 year 7 months for P1; 11 months for P2). The patients did not learn to move or crawl but could lift their heads when in a prone position, and they could move their (upper) extremities voluntarily. They were unable to sit unsupported and required also head support in the wheelchair to maintain head control.

The patients had profound intellectual disability and communicated by closing their eyes, crying, smiling, and making sounds reciprocally. Visual fixation and eye contact were at best very short-lived, but most often investigators described them to be absent. However, the patients reacted to light, as well as high-contrast pictures. Mild optic atrophy or pale optic discs were observed in the patients, but this was considered secondary to the cortical visual impairment. Hyperopia and alternating

strabismus were also noted in both patients and the retinal epithelium of P1 had a granular appearance.

The head circumferences (HC) were within normal limits at birth (HC of P1 was 0 standard deviations (SD) and HC of P2 – 1.5 SD), but severe microcephaly developed during the first year: head circumferences were 4–5 SD below average at 7 months of age and 7 SD below average in childhood.

Both patients have undergone tympanostomies (n=1 for P1 and n=4 for P2) and they have been hospitalized due to recurrent pneumonias. By 15 years of age, P1 has had altogether 35 infections requiring antibiotic treatment: 9 otitis media, at least 11 pneumonias, as well as other upper respiratory tract infections. He was also hospitalized for 9 days due to simultaneous infection with varicella zoster and adenovirus at 6 years 2 months of age. By 11 years of age, P2 has had altogether 34 infections requiring antibiotic treatment: 18 otitis media, at least 6 pneumonias, other upper respiratory tract infections, and 3 skin infections.

Due to feeding problems especially during infections, percutaneous endoscopy gastrostomy (PEG) tube was inserted in childhood (P1 at 5 years 3 months; P2 at 2 years 7 months). In addition, P1 has been operated due to retention of testis (3 years 8 months). P1 was evaluated due to scoliosis and painless neurological hip dislocation on the right side (6 years 10 months). P2 has also scoliosis.

At 8 years of age, P2 was investigated due to fluctuating diarrhea with signs of gastrointestinal bleeding and intermittent mucus, as well as repeatedly increased fecal calprotectin levels (375–996 μg/g; normal range <100). During the follow-up, fecal antitrypsin levels also increased from 10 μg/g to >1130 μg/g (normal range <268 μg/g). Macroscopical colonoscopy findings were suggestive of colitis on the left side of the colon (including the descending colon and sigma), but only unspecific patchy lymphatic hyperplasia was seen microscopically. However, a treatment trial with corticosteroids finally ameliorated the gastrointestinal symptoms and normalized the calprotectin levels. Gastrointestinal symptom aggravation soon after the 6-week corticosteroid regimen led to the initiation of permanent medication: as mesalazine could not be used with the feeding tube, azathioprine was chosen.

The brain MRI was performed on P1 and P2 during the first week of life (4 days and 5 days of age, respectively) with very similar findings (Figure S1). The MRIs showed cortical malformations with absent or reduced gyration (with a posterior-

anterior gradient), dysgyria, as well as polymicrogyria in the parietal (and frontal) regions. These findings were initially described as agyria and pachygyria, but since the thickness of the cortex was normal, the findings should rather be described as simplified gyral pattern. Posterior double cortex was also noted and subcortical band heterotopia was suspected. The Sylvian fissures were 'open' (i.e. the superior and inferior borders were not fully apposed). The pons was hypoplastic and bilateral intraventricular cysts were noted within the mildly dilated ventricles in both patients. The sloping forehead in the MRIs was suggestive of microcephaly although the head circumferences were not microcephalic at birth.

A repeat brain MRI of P1 at 7 years 11 months of age showed severe microcephaly, abnormal cortical gyration with lack of most gyri but polymicrogyria-like changes particularly in the perisylvian region and the posterior region, hypoplasia of the pons, findings suggestive of brain atrophy (i.e. increase in the size of the lateral ventricles and subarachnoid space), thin corpus callosum (posterior part not properly visualized). In addition, the parietal region showed bilateral gliotic changes of unknown cause that had developed after the previous MRI (Figure S2).

The EEG demonstrated (interictal) epileptiform discharges in both patients already during the first week of life. In P1, classical hypsarrhythmia had appeared by the age of 4 months. Despite a video EEG performed at ~5 months of age, ictal abnormality was recorded for the first time at ~6 years of age: voltage attenuation was associated with a tonic seizure (lasting approximately 1 min 20 sec). In P2, the EEG at the age of 1 year 5 months showed hypsarrythmia during sleep and focal discharges.

At 5 years and 1 month of age, the electroneuromyography (ENMG) of P1 did not show signs of neuropathy. The muscle biopsy sample of P1 at 5 years 3 months of age showed normal histology and mitochondrial respiratory chain activities.

Supplemental methods

Previous genetic testing

Prenatal chromosomal analysis, as well as oligonucleotide array-based comparative genomic hybridization (aCGH) (244K; Agilent Technologies), were normal in P1. No diagnostic changes had been observed in the following genetic testing utilizing blood DNA of P2: oligonucleotide array-based comparative genomic hybridization (180K; Agilent Technologies), next-generation sequencing (NGS) panel testing including 16 genes (*ARX*, *DCX*, *PAFAH1BI*, *TUBA1A*, *TUBB2B*, *GPR56*, *TUBA8*, *NDE1*, *RELN*, *LAMB1*, OCLN, *VLDLR*, *RTTN*, *CASK*, *TUBB3*, and *DYNC1H1*), copy number analysis of PAFAH1B1 and DCX by multiplex ligation-dependent probe amplification (MLPA), clinical exome sequencing (performed in 2015), and mtDNA sequencing (NGS).

Additional genome sequencing data findings

Virtual gene panel for brain malformations also yielded one heterozygous nonsense variant in exon 19 of *LAMA2* NM_000426:c.2578G>T, p.(Gly860*) that was shared by both patients and the father, and absent from population databases. This finding most likely represents the carrier status of recessively inherited merosin deficient muscular dystrophy (1), which is not compatible with the phenotype of our patients.

Table S1. Antibodies used in immunofluorescence staining.

Primary antibodies	Manufacturer	Host	Identifier	Used dilution
**Beta-tubulin**	Sigma-Aldrich	mouse	T4026	1:200
**EEA1**	BD Biosciences	mouse	610457	1:50
**Hoechst 33242**	Life technologies		H1399	1:5000
**Lamp1**	DHBH	mouse	H4A3	1:100
**Lamp2**	DSHB	mouse	H4B4	1:100
**Limp (Lamp3)**	DSHB	mouse	H5C6	1:100
**Phalloidin**	Invitrogen		A22287	1:50
**Alexa Fluor™ Plus 405 Phalloidin**	Invitrogen		A30104	1:400
**TfR**	Invitrogen	mouse	13-6800	1:100
**Vinculin**	Sigma-Aldrich	mouse	V9131	1:200
**Girdin (GIV)**	Abcam	rabbit	AB179481	1:100
**EEA1**	Invitrogen	mouse	MA5-31575	1:100
**CD107a (LAMP-1)**	eBioscience	mouse	14-1079-80	1:100
**Secundary antibodies**	**Manufacturer**	**Host**	**Identifier**	**Used dilution**
**Alexa Fluor 568 goat anti mouse**	Invitrogen	goat	A11004	1:1000
**Alexa Fluor 594 goat anti rabbit**	Invitrogen	goat	C14784	1:500
**Alexa Fluor 488 goat anti rabbit**	Invitrogen	goat	A28175	1:500

Table S2. Analysis Sequence for Harmony 4.9

**Input Image**	**Input**		
	Flatfield Correction : Basic<br>Stack Processing : Maximum Projection<br>Create Global Image<br>Min. Global Binning : Dynamic		
**Find Nuclei**	**Input**	**Method**	**Output**
	Channel : HOECHST 33342 (global)<br>ROI : Imaged Area (global)<br>ROI Region : Imaged Area	Method : C<br>Common Threshold : 0.2<br>Area : > 30 µm²<br>Splitting Coefficient : 6.3<br>Individual Threshold : 0.5<br>Contrast : > 0.14	Output Population : Nuclei (global)
**Calculate Morphology Properties**	**Input**	**Method**	**Output**
	Population : Nuclei (global)<br>Region : Nucleus	Method : Standard<br>Area<br>Roundness<br>Width<br>Length<br>Ratio Width to Length	Property Prefix : Nucleus
**Select Population**	**Input**	**Method**	**Output**
	Population : Nuclei (global)	Method : Filter by Property<br>Nucleus Area [µm²] : > 50<br>Nucleus Roundness : > 0.6<br>Nucleus Area [µm²] : < 900<br>Boolean Operations : F1 and F2 and F3	Output Population : Nuclei (global) Selected
**Calculate Intensity Properties**	**Input**	**Method**	**Output**
	Channel : Alexa 568 (global)<br>Population : Nuclei (global) Selected<br>Region : Nucleus	Method : Standard<br>Mean<br>Sum	Property Prefix : Intensity Nucleus Alexa 568 (global)
**Select Cell Region**	**Input**	**Method**	**Output**
	Population : Nuclei (global) Selected	Method : Resize Region [%]<br>Region Type : Ring Region<br>Outer Border : -100 %<br>Inner Border : 0 %	Output Region : Ring Region
**Find Cytoplasm**	**Input**	**Method**	**Output**
	Channel : Alexa 647 (global)<br>Nuclei : Nuclei (global) Selected	Method : D<br>Individual Threshold : 0.35<br>Restrictive Region : Imaged Area	
**Find Spots**	**Input**	**Method**	**Output**
	Channel : Alexa 568 (global)<br>ROI : Nuclei (global) Selected<br>ROI Region : Cell	Method : B<br>Detection Sensitivity : 0.2<br>Splitting Sensitivity : 0.833<br>Calculate Spot Properties	Output Population : Spots (global)
**Calculate Intensity Properties**	**Input**	**Method**	**Output**
	Channel : Alexa 488 (global)<br>Population : Spots (global)<br>Region : Spot	Method : Standard<br>Mean<br>Sum	Property Prefix : Intensity Spot Alexa 488 (global)
**Calculate Intensity Properties**	**Input**	**Method**	**Output**
	Channel : Alexa 568 (global)<br>Population : Spots (global)<br>Region : Spot	Method : Standard<br>Mean<br>Sum	Property Prefix : Intensity Spot Alexa 568 (global)
**Calculate Intensity Properties**	**Input**	**Method**	**Output**
	Channel : Alexa 647 (global)<br>Population : Spots (global)<br>Region : Spot	Method : Standard<br>Mean<br>Sum	Property Prefix : Intensity Spot Alexa 647 (global)
**Calculate Morphology Properties**	**Input**	**Method**	**Output**
	Population : Spots (global)<br>Region : Spot	Method : Standard<br>Area<br>Roundness<br>Width<br>Length<br>Ratio Width to Length	Property Prefix : Spot
**Select Cell Region**	**Input**	**Method**	**Output**
	Population : Nuclei (global) Selected	Method : Resize Region [%]<br>Region Type : Cytoplasm Region<br>Outer Border : 5 %<br>Inner Border : 45 %	Output Region : Cytoplasm Region
**Calculate Intensity Properties**	**Input**	**Method**	**Output**
	Channel : Alexa 488 (global)<br>Population : Nuclei (global) Selected<br>Region : Cytoplasm	Method : Standard<br>Mean<br>Sum	Property Prefix : Intensity Cytoplasm Alexa 488 (global)
**Calculate Intensity Properties**	**Input**	**Method**	**Output**
	Channel : Alexa 568 (global)<br>Population : Nuclei (global) Selected<br>Region : Cytoplasm Region	Method : Standard<br>Mean<br>Sum	Property Prefix : Intensity Cytoplasm Region Alexa 568 (global)
**Calculate Intensity Properties**	**Input**	**Method**	**Output**
	Channel : Alexa 647 (global)<br>Population : Nuclei (global) Selected<br>Region : Cytoplasm Region	Method : Standard<br>Mean<br>Sum	Property Prefix : Intensity Cytoplasm Region Alexa 647 (global)
**Calculate Morphology Properties**	**Input**	**Method**	**Output**
	Population : Nuclei (global) Selected<br>Region : Cytoplasm Region	Method : Standard<br>Area<br>Roundness<br>Width<br>Length<br>Ratio Width to Length	Property Prefix : Cytoplasm Region
**Select Cell Region**	**Input**	**Method**	**Output**
	Population : Nuclei (global) Selected	Method : Resize Region [%]<br>Region Type : Membrane Region<br>Outer Border : -5 %<br>Inner Border : 5 %	Output Region : Membrane Region
**Calculate Intensity Properties**	**Input**	**Method**	**Output**
	Channel : Alexa 488 (global)<br>Population : Nuclei (global) Selected<br>Region : Membrane Region	Method : Standard<br>Mean<br>Sum	Property Prefix : Intensity Membrane Region Alexa 488 (global)
**Calculate Intensity Properties**	**Input**	**Method**	**Output**
	Channel : Alexa 568 (global)<br>Population : Nuclei (global) Selected<br>Region : Membrane Region	Method : Standard<br>Mean<br>Sum	Property Prefix : Intensity Membrane Region Alexa 568 (global)

**Calculate Intensity Properties**	**Input**	**Method**	**Output**
	Channel : Alexa 647 (global)<br>Population : Nuclei (global) Selected<br>Region : Membrane Region	Method : Standard<br>Mean<br>Sum	Property Prefix : Intensity Membrane Region Alexa 647 (global)
**Calculate Morphology Properties**	**Input**	**Method**	**Output**
	Population : Nuclei (global) Selected<br>Region : Membrane Region	Method : Standard<br>Area<br>Roundness<br>Width<br>Length<br>Ratio Width to Length	Property Prefix : Membrane Region
**Select Region**	**Input**	**Method**	**Output**
	Population : Nuclei<br>Region : Membrane Region	Method : Restrict by Mask<br>Population : Selected filaments<br>Mask Region : Filaments	Output Region : Filaments on borders
**Calculate Intensity Properties**	**Input**	**Method**	**Output**
	Channel : Alexa 568<br>Population : Nuclei<br>Region : Filaments on borders	Method : Standard<br>Mean<br>Sum	Property Prefix : Intensity Filaments on borders Alexa 568
**Calculate Morphology Properties**	**Input**	**Method**	**Output**
	Population : Nuclei<br>Region : Filaments on borders	Method : Standard<br>Area<br>Roundness<br>Width<br>Length<br>Ratio Width to Length	Property Prefix : Filaments on borders
**Filter Image**	**Input**	**Method**	**Output**
	Channel : Alexa 488	Method : Sliding Parabola<br>Curvature : 10	Output Image : Sliding Parabola (2)
**Filter Image**	**Input**	**Method**	**Output**
	Channel : Sliding Parabola (2)	Method : Smoothing<br>Filter : Gaussian<br>Width : 2 px	Output Image : Vinculin
**Find Spots**	**Input**	**Method**	**Output**
	Channel : Vinculin<br>ROI : Nuclei<br>ROI Region : Filaments	Method : D<br>Detection Sensitivity : 0.6<br>Splitting Sensitivity : 0.05<br>Background Correction : 0.3<br>Calculate Spot Properties	Output Population : Focal adhesions
**Calculate Intensity Properties**	**Input**	**Method**	**Output**
	Channel : Alexa 488<br>Population : Focal adhesions<br>Region : Spot	Method : Standard<br>Mean<br>Sum	Property Prefix : Intensity Girdin Focal adhesions
**Calculate Intensity Properties**	**Input**	**Method**	**Output**
	Channel : Alexa 568<br>Population : Focal adhesions<br>Region : Spot	Method : Standard<br>Mean<br>Sum	Property Prefix : Intensity Vinculin Focal adhesions
**Calculate Morphology Properties**	**Input**	**Method**	**Output**
	Population : Focal adhesions<br>Region : Spot	Method : Standard<br>Area<br>Roundness<br>Width<br>Length<br>Ratio Width to Length	Property Prefix : Focal adhesions
**Calculate Morphology Properties**	**Input**	**Method**	**Output**
	Population : Nuclei (global) Selected<br>Region : Cell	Method : Standard<br>Area<br>Roundness<br>Width<br>Length<br>Ratio Width to Length	Property Prefix : Cell
**Calculate Intensity Properties**	**Input**	**Method**	**Output**
	Channel : Alexa 488 (global)<br>Population : Nuclei (global) Selected<br>Region : Cell	Method : Standard<br>Mean<br>Sum	Property Prefix : Intensity Cell Alexa 488 (global)
**Calculate Intensity Properties**	**Input**	**Method**	**Output**
	Channel : Alexa 568 (global)<br>Population : Nuclei (global) Selected<br>Region : Cell	Method : Standard<br>Mean<br>Sum	Property Prefix : Intensity Cell Alexa 568 (global)
**Calculate Intensity Properties**	**Input**	**Method**	**Output**
	Channel : Alexa 647 (global)<br>Population : Nuclei (global) Selected<br>Region : Cell	Method : Standard<br>Mean<br>Sum	Property Prefix : Intensity Cell Alexa 647 (global)
**Filter Image**	**Input**	**Method**	**Output**
	Channel : Alexa 647	Method : Sliding Parabola<br>Curvature : 1	Output Image : Sliding Parabola
**Filter Image**	**Input**	**Method**	**Output**
	Channel : Sliding Parabola	Method : Smoothing<br>Filter : Gaussian<br>Width : 0.5 px	Output Image : Gaussian Smoothed
**Filter Image**	**Input**	**Method**	**Output**
	Channel : Sliding Parabola	Method : Texture SER<br>Filter : SER Ridge<br>Scale : 2 px<br>Normalization by : Kernel	Output Image : SER Ridge
**Find Image Region**	**Input**	**Method**	**Output**
	Channel : SER Ridge<br>ROI : Nuclei<br>ROI Region : Cell	Method : Common Threshold<br>Threshold : 0.45<br>Split into Objects<br>Area : > 2 px²<br>Fill Holes	Output Population : Filaments<br>Output Region : Filaments
**Calculate Morphology Properties**	**Input**	**Method**	**Output**
	Population : Filaments<br>Region : Filaments	Method : Standard<br>Area<br>Roundness<br>Width<br>Length<br>Ratio Width to Length	Property Prefix : Filaments
**Calculate Intensity Properties**	**Input**	**Method**	**Output**
	Channel : Alexa 488	Method : Standard	Property Prefix : Intensity Filaments Alexa 488

	Population : Filaments<br>Region : Filaments	Mean<br>Sum	
**Select Population**	**Input**	**Method**	**Output**
	Population : Filaments	Method : Filter by Property<br>Filaments Ratio Width to Length : < 0.3<br>Intensity Filaments Alexa 488 Mean : > 100<br>Filaments Area [µm²] : > 3<br>Filaments Area [µm²] : < 2000<br>Boolean Operations : F1 and F2 and F3 and F4	Output Population : Selected filaments
**Calculate Morphology Properties**	**Input**	**Method**	**Output**
	Population : Selected filaments<br>Region : Filaments	Method : Standard<br>Area<br>Roundness<br>Width<br>Length<br>Ratio Width to Length	Property Prefix : Selected filaments
**Calculate Intensity Properties**	**Input**	**Method**	**Output**
	Channel : Alexa 488<br>Population : Selected filaments<br>Region : Filaments	Method : Standard<br>Mean<br>Sum	Property Prefix : Intensity Selected filaments Alexa 488
**Calculate Intensity Properties**	**Input**	**Method**	**Output**
	Channel : Alexa 568<br>Population : Selected filaments<br>Region : Filaments	Method : Standard<br>Mean<br>Sum	Property Prefix : Intensity Selected filaments Alexa 568
**Calculate Intensity Properties**	**Input**	**Method**	**Output**
	Channel : Alexa 647<br>Population : Selected filaments<br>Region : Filaments	Method : Standard<br>Mean<br>Sum	Property Prefix : Intensity Selected Filaments Alexa 647
**Calculate Properties**	**Input**	**Method**	**Output**
	Population : Nuclei (global) Selected	Method : By Related Population<br>Related Population : Spots (global)<br>Number of Spots (global)<br>Intensity Spot Alexa 488 (global) Mean<br>Intensity Spot Alexa 488 (global) Sum<br>Intensity Spot Alexa 568 (global) Mean<br>Intensity Spot Alexa 568 (global) Sum<br>Intensity Spot Alexa 647 (global) Mean<br>Intensity Spot Alexa 647 (global) Sum<br>Spot Area [µm²]<br>Spot Roundness<br>Spot Width [µm]<br>Spot Length [µm]<br>Spot Ratio Width to Length	Property Suffix : per Cell
**Select Region**	**Input**	**Method**	**Output**
	Population : Nuclei (global) Selected<br>Region : Ring Region	Method : Restrict by Mask<br>Population : Nuclei (global) Selected<br>Mask Region : Cytoplasm Region	Output Region : Ring region final
**Select Population**	**Input**	**Method**	**Output**
	Population : Spots (global)	Method : Select by Mask<br>Region : Spot<br>Mask Population : Nuclei (global) Selected<br>Mask Region : Ring region final<br>Select by : Geometrical Center	Output Population : Spots (global) in ring region final
**Calculate Properties**	**Input**	**Method**	**Output**
	Population : Nuclei (global) Selected	Method : By Related Population<br>Related Population : Spots (global) in ring region final<br>Number of Spots (global) in ring region final<br>Intensity Spot Alexa 488 (global) Mean<br>Intensity Spot Alexa 488 (global) Sum<br>Intensity Spot Alexa 568 (global) Mean<br>Intensity Spot Alexa 568 (global) Sum<br>Intensity Spot Alexa 647 (global) Mean<br>Intensity Spot Alexa 647 (global) Sum<br>Spot Area [µm²]<br>Spot Roundness<br>Spot Width [µm]<br>Spot Length [µm]<br>Spot Ratio Width to Length	Property Suffix : per Cell
**Select Population**	**Input**	**Method**	**Output**
	Population : Spots (global)	Method : Select by Mask<br>Region : Spot<br>Mask Population : Nuclei (global) Selected<br>Mask Region : Cytoplasm<br>Select by : Geometrical Center	Output Population : Spots (global) in cytoplasm

Acapella version: 5.0.1.124082. Timestamp: 2024-02-05 11:38:04 +0200.

Table S3. Cell organelles of the endolysosomal pathway were altered in P1 fibroblasts compared with control fibroblasts. The data is presented as mean±SD. EEA1 = Early endosomes; LAMP1, LAMP2 = lysosome-associated membrane proteins I and II; LIMP = lysosomal glycoprotein III; TfR = transferrin receptor; EGF = epidermal growth factor.

Vesicles (%) in the perinuclear region	GM05399	GM08398	GM09503	Patient 1
EEA1 without EGF	68±28	74±29	76±27	81±26
LAMP1 without EGF	57±24	63±24	61±25	69±23
LAMP2 without EGF	64±27	70±27	66±28	75±24
LIMP without EGF	55±25	61±25	60±25	68±23
TfR positive without EGF	65.9±27	68±28.7	67±28.3	72.5±26.2
TfR positive with EGF	65.8±28.5	73.3±25.5	71.7±27.3	81.6±25
Number of vesicles in the cytoplasm	GM05399	GM08398	GM09503	Patient 1
EEA1 without EGF	18±19	9±9	11±12	7±8
LAMP1 without EGF	56±56	39±55	53±67	32±35
LAMP2 without EGF	45±60	38±62	45±71	23±33
LIMP without EGF	66±70	51±75	60±80	36±42
TfR positive without EGF	21±23	13±25	28±40	11±22
TfR positive with EGF	26±34	17±22	16±23	7±10
Vesicle size (μm^2)	GM05399	GM08398	GM09503	Patient 1
EEA1 without EGF	4.2±2	3.6±2.7	3.8±2.3	3.2±2
LAMP1 without EGF	11.1±4.7	9.6±4.3	10.1±4.2	9.6±4.2
LAMP2 without EGF	13.5±7.8	14.9±9	12.2±7.5	11.6±7.6
LIMP without EGF	10±3.7	9.2±3.8	9.8±3.9	8.9±3.8
TfR positive without EGF	7.2±6.5	8±24.4	7.5±6.5	5.4±4.5
TfR positive with EGF	7.6±6.3	9.1±7.9	8±7	7.2±7

Table S4. Welch Two Sample t-test p-values.

Cell size (µm^2)	**GM05399**	**GM08398**	**GM09503**
**Patient 1**	2.2e-16	2.2e-16	2.2e-16
**GM05399**	-	2.2e-16	2.2e-16
**GM08398**	-	-	0.05676
Actin filament area (%) per cell	**GM05399**	**GM08398**	**GM09503**
**Patient 1**	2.2e-16	2.2e-16	2.2e-16
**GM05399**	-	2.2e-16	0.004527
**GM08398**	-	-	3.512e-07
Number of focal adhesions per cell	**GM05399**	**GM08398**	**GM09503**
**Patient 1**	2.2e-16	2.2e-16	2.2e-16
**GM05399**	-	2.2e-16	2.2e-16
**GM08398**	-	-	5.694e-07
EEA1 vesicles (%) in the perinuclear region	**GM05399**	**GM08398**	**GM09503**
**Patient 1**	2.2e-16	2.2e-16	2.2e-16
**GM05399**	-	2.2e-16	2.2e-16
**GM08398**	-	-	0.04053
LAMP1 vesicles (%) in the perinuclear region	**GM05399**	**GM08398**	**GM09503**
**Patient 1**	2.2e-16	2.2e-16	2.2e-16
**GM05399**	-	2.2e-16	7.029e-13
**GM08398**	-	-	7.043e-07
LAMP2 vesicles (%) in the perinuclear region	**GM05399**	**GM08398**	**GM09503**
**Patient 1**	2.2e-16	2.2e-16	2.2e-16
**GM05399**	-	2.2e-16	0.0005096
**GM08398**	-	-	1.047e-07
LIMP vesicles (%) in the perinuclear region	**GM05399**	**GM08398**	**GM09503**
**Patient 1**	2.2e-16	2.2e-16	2.2e-16
**GM05399**	-	2.2e-16	2.2e-16
**GM08398**	-	-	0.04565
TfR vesicles (%) in the perinuclear region EGF-	**GM05399**	**GM08398**	**GM09503**
**Patient 1**	2.2e-16	4.947e-10	1.53e-14
**GM05399**	-	0.0004059	0.05291
**GM08398**	-	-	0.1608
TfR vesicles (%) in the perinuclear region EGF+	**GM05399**	**GM08398**	**GM09503**
**Patient 1**	2.2e-16	2.2e-16	2.2e-16
**GM05399**	-	2.2e-16	2.2e-16
**GM08398**	-	-	0.01315
EEA1 vesicle number per cell	**GM05399**	**GM08398**	**GM09503**
**Patient 1**	2.2e-16	2.2e-16	2.2e-16
**GM05399**	-	2.2e-16	2.2e-16
**GM08398**	-	-	2.2e-16
LAMP1 vesicle number per cell	**GM05399**	**GM08398**	**GM09503**
**Patient 1**	2.2e-16	2.745e-16	2.2e-16
**GM05399**	-	2.2e-16	0.02465

**GM08398**	-	-	2.2e-16
LAMP2 vesicle number per cell	**GM05399**	**GM08398**	**GM09503**
**Patient 1**	2.2e-16	2.2e-16	2.2e-16
**GM05399**	-	2.361e-06	0.9409
**GM08398**	-	-	0.0001203
LIMP vesicle number per cell	**GM05399**	**GM08398**	**GM09503**
**Patient 1**	2.2e-16	2.2e-16	2.2e-16
**GM05399**	-	2.2e-16	7.369e-05
**GM08398**	-	-	1.201e-06
TfR vesicle number per cell EGF-	**GM05399**	**GM08398**	**GM09503**
**Patient 1**	2.2e-16	0.002235	2.2e-16
**GM05399**	-	2.2e-16	2.2e-16
**GM08398**	-	-	2.2e-16
TfR vesicle number per cell EGF+	**GM05399**	**GM08398**	**GM09503**
**Patient 1**	2.2e-16	2.2e-16	2.2e-16
**GM05399**	-	2.2e-16	2.2e-16
**GM08398**	-	-	0.4592
EEA1 vesicle size without EGF (μm^2)	**GM05399**	**GM08398**	**GM09503**
**Patient 1**	2.2e-16	2.2e-16	2.2e-16
**GM05399**	-	2.2e-16	3.954e-12
**GM08398**	-	-	0.0002215
LAMP1 vesicle size without EGF (μm^2)	**GM05399**	**GM08398**	**GM09503**
**Patient 1**	2.2e-16	0.855	5.311e-08
**GM05399**	-	2.2e-16	2.2e-16
**GM08398**	-	-	3.305e-08
LAMP2 vesicle size without EGF (μm^2)	**GM05399**	**GM08398**	**GM09503**
**Patient 1**	2.2e-16	2.2e-16	0.003215
**GM05399**	-	4.093e-13	5.524e-14
**GM08398**	-	-	2.2e-16
LIMP vesicle size without EGF (μm^2)	**GM05399**	**GM08398**	**GM09503**
**Patient 1**	2.2e-16	2.865e-05	2.2e-16
**GM05399**	-	2.2e-16	0.06983
**GM08398**	-	-	5.361e-12
TfR vesicle size without EGF (μm^2)	**GM05399**	**GM08398**	**GM09503**
**Patient 1**	2.2e-16	1.147e-08	2.2e-16
**GM05399**	-	0.09154	0.03412
**GM08398**	-	-	0.3259
TfR vesicle size with EGF (μm^2)	**GM05399**	**GM08398**	**GM09503**
**Patient 1**	0.002329	2.2e-16	1.706e-08
**GM05399**	-	2.2e-16	0.002033
**GM08398**	-	-	6.737e-09
The cytoplasm region (%) of total cell area	**GM05399**	**GM08398**	**GM09503**
**Patient 1**	2.2e-16	2.341e-06	1.406e-13

**GM05399**	-	0.0003111	0.5431
**GM08398**	-	-	0.007979
The membrane region (%) of total cell area	**GM05399**	**GM08398**	**GM09503**
**Patient 1**	2.2e-16	2.2e-16	2.2e-16
**GM05399**	-	2.2e-16	2.2e-16
**GM08398**	-	-	0.2613
Actin filament area (μm^2)	**GM05399**	**GM08398**	**GM09503**
**Patient 1**	2.2e-16	1.6e-07	0.000632
**GM05399**	-	5.275e-08	1.387e-11
**GM08398**	-	-	0.1318
Actin filament width (μm)	**GM05399**	**GM08398**	**GM09503**
**Patient 1**	0.04997	0.008521	0.01435
**GM05399**	-	0.4345	0.497
**GM08398**	-	-	0.9507
Actin filament length (μm)	**GM05399**	**GM08398**	**GM09503**
**Patient 1**	2.2e-16	2.2e-16	4.521e-06
**GM05399**	-	2.699e-16	2.2e-16
**GM08398**	-	-	0.001673
Actin filament width to length ratio	**GM05399**	**GM08398**	**GM09503**
**Patient 1**	2.2e-16	2.2e-16	2.2e-16
**GM05399**	-	0.0001165	2.2e-16
**GM08398**	-	-	2.172e-12
Vinculin spots (μm^2)	**GM05399**	**GM08398**	**GM09503**
**Patient 1**	1.789e-08	0.1819	0.01447
**GM05399**	-	2.2e-16	2.2e-16
**GM08398**	-	-	0.09465
Vinculin intensity in focal adhesions	**GM05399**	**GM08398**	**GM09503**
**Patient 1**	0.000101	0.2411	0.0322
**GM05399**	-	2.2e-16	2.2e-16
**GM08398**	-	-	0.1053
Vinculin area to number ratio	**GM05399**	**GM08398**	**GM09503**
**Patient 1**	1.782e-13	1.376e-10	0.000381
**GM05399**	-	0.3644	8.431e-05
**GM08398**	-	-	0.003093

Table S5*. Immunophenotype of the patients.*

Immune cells	Reference range 12–18 years	Patient 1 (P1; m) 15.5 years	Patient 2 (P2; f) 11.5 years
**Leukocytes E9/L**	**4.5–13.5**	**7.7**	**4.20**
Neutrophils	1.5–5.9	3.92	1.71
Eosinophils	0.0–0.46	0.12	0.60
Monocytes	0.15–0.28	0.61	0.37
**Lymphocytes E9/L**	**1.40–3.30**	**3.03**	**1.53 E9/L**
B cells (CD19+)	0.11–0.57	0.39	0.10
T cells (CD3+)	1.00–2.20	2.72	1.59
T helper (CD3+CD4+)	0.53–1.30	1.99	1.132
T killer (CD3+CD8+)	0.33–0.92	0.74	0.37
NK cells (CD3- CD16/56)	0.07–0.48	0.19	0.01
**Dendritic cells % leuc**	**% leuc**	**% leuc**	**% leuc**
Plasmacytoid (pDC) (lin-HLA-DR+CD123+CD11c-)	0.1–0.3	0.05	<0.01
Monocytoid (mDC) (lin-HLA-DR+CD123-CD11c+)	0.1–0.3	0.02	0.01
**Immunoglobulins g/L**	**g/L**	**g/L**	**g/L**
P-IgG	5.6–14.2	8.7	20.5
P-IgA	0.35–2.99	2.55	2.76
P-IgM	0.56–2.66	0.79	1.20
S-IgE kU/L	0–320 kU/L	<2 kU/L	26 kU/L
**Functional tests**			
Lymphocyte (CD4+ and CD8+) proliferative mitogen response	PHA<br><br>ConA	CD4+ 91% blasts, N<br>CD8+ 94% blasts, N<br><br>CD4+ 89% blasts, N<br>CD8+ 91% blasts, N	CD4+ 96% blasts, N<br>CD8+ 90% blasts, N<br><br>CD4+ 95% blasts, N<br>CD8+ 88% blasts, N
Neutrophil oxidative burst (Rhodamine test)		Normal	Normal
Treg expression/number: -FOXP3^{+}CD25highCD4^{+} stain	Controls: 3.9% and 2.63 %	1.68% (vs. 3.9%)	1.96% (vs. 2.63%)

Immune cells	Reference range 12–18 years	Patient 1 (P1; m) 15.5 years	Patient 2 (P2; f) 11.5 years
-CD127low/negCD25highCD4+ stain<br><br>Treg suppressive efficacy	4.83% and 2.98%	1.55% (vs. 4.83%)<br><br>Normal	2.20% (vs. 2.98%)<br><br>Assay failed
Tetanus antibodies (CIteAb) after toxoid vaccine (protein Ag)	>0.1 IU /mL	0.36 IU/mL, N	1.4 IU/ml, N
Diphteria antibodies (CodiAb) after dT vaccine	>0.1 IU/mL	0.07 IU/mL	0.28 IU/mL, N
Pneumococcus antibodies (SpnAb) against polysaccharide vaccine serotypes (Pneumovax)	>0.35 ug/mL	2/9 titers > 5.percentile for vaccinated at 7–17 years.<br>Titers >0.35 ug/mL against 4/9 vaccine serotypes.<br>Lowered response for age	3/9 titers > 5.percentile for vaccinated at 7–17 years.<br>Titers >0.35 ug/mL against 4/9 vaccine serotypes.<br>Lowered response for age

Table S6. *T and B lymphocyte flow cytometry results.*

**Lymphocyte differential (Fc)**	**Cell type**	**Control / Ref range (%)**	**Patient 1 (P1; m) 15.5 years**	**Patient 2 (P2; f) 11.5 years**
**$CD19^+$ B cells**	**$CD19^+$**			
Transitional	$CD38^{hi}IgM^{hi}$	m. 7%; 1–13%	4.6%	4.4%
Naive	$CD27^-IgD^+$	m. 66%; 51–83%	52.3%	59.4%
Memory	$CD27^+$	m. 28%; 13–48%	45.4%	35.3%
Marginal zone MZ	$CD27^+IgD^+IgM^+$	m. 12%; 5–18%	40.2%	12.8%
Switched memory	$CD27^+IgD^-IgM^-$	m. 16%; 9–26%	1.9%	12.0%
Plasmablasts	$CD38^{++}IgM^-$	m. 2%; 1–7%	0.1%	3.2%
Activated	$CD38^{low}CD21^{low}$	m. 6%; 3–9%	15.9%	7.4%
**$CD3^+$ T cells**	**$CD3^+$**			
Naive	$CCR7^+CD45RA^+$		54%	58%
DNT	$CD4^-CD8^-TCRab^+$		0.8%	0.8%
Treg	$CD25^{hi}CD127^{lo}$	m. 4.3%; 2.8–6.4%	3.0%, low normal	2.8%, low normal
**$CD3^+CD4^+$ cells**	**$CD3^+CD4^+$**			
Naive	$CCR7^+CD45RA^+$	m. 37.2%; 20.5–54.8%	55.2%	47.7%
TCM	$CCR7^+CD45RO^+$		41.3%	47.6%
TEM	$CCR7^-CD45RA^-$	19.9–52.4% (adults)	3.4%	4.9%
TEMRA	$CCR7^-CD45RA^+$		0.1%	0.2%
Activated	$HLADR^+CD38^-$	m. 5.2%; 2.4–9.6%	1.5%	2.4%
	$HLADR^-CD38^+$	m. 60%; 40.4–72.9%	73.5%	67.4%
	$HLADR^+CD38^+$	m. 2.0%; 0.9–4.6%	3.5%	2.8%
RTE	$CD45RA^+CD62L^+CD31^+$	m. 31.0%; 14.4–38.3%	42.6%	42.5%

**Lymphocyte differential (Fc)**	**Cell type**	**Control / Ref range (%)**	**Patient 1 (P1; m) 15.5 years**	**Patient 2 (P2; f) 11.5 years**
Treg	$CD25^{hi}CD127^{lo}$		4.2%	4.3%
**$CD3^{+}CD8^{+}$ T cells**	**$CD3^{+}CD8^{+}$**			
Naive	$CCR7^{+}CD45RA^{+}$	m. 43.8%; 18.8–71%	55.5%	84.0%
TCM	$CCR7^{+}CD45RO^{+}$		15.4%	5.9%
TEM	$CCR7^{-}CD45RA^{-}$	14.6–63.0% (adults)	9.1%	4.2%
TEMRA	$CCR7^{-}CD45RA^{+}$		19.9%	6.0%
Activated	$HLADR^{+}CD38^{-}$	m.12.9%; 3.8–32.4%	10.4%	2.7%
	$HLADR^{-}CD38^{+}$	m. 53.4%; 30.3–78.5%	37.4%	64.3%
	$HLADR^{+}CD38^{+}$	m. 7.0%; 1.4–21.4%	6.7%	9.6%

* m. = mean

Table S7. *The virtual gene panel includes 230 genes associated with brain malformations.*

ACTB	*DCLK2*	*INPP5K*	*PAFAH1B1*	*SLC25A19*
ACTG1	*DCX*	*ISCA1*	*PAFAH2*	*SLC25A24*
ADGRG1	*DDX3X*	*ISPD*	*PALLD*	*SMO*
AFDN	*DHX37*	*KATNB1*	*PAX6*	*SNAP29*
AHI1	*DOCK6*	*KIAA0586*	*PCGF2*	*SNRPN*
AIMP1	*DYNC1H1*	*KIAA1109*	*PEX1*	*SRD5A3*
AKT1	*EML1*	*KIF1BP*	*PEX10*	*SRPX2*
AKT3	*EMX2*	*KIF2A*	*PEX13*	*SUFU*
ARFGEF2	*EOMES*	*KIF5C*	*PEX14*	*SVIL*
ARHGAP31	*EPG5*	*KLHL15*	*PEX2*	*TAF13*
ARHGEF9	*ERCC1*	*KRAS*	*PEX26*	*TBC1D20*
ARX	*ERMARD*	*L1CAM*	*PEX7*	*TBL1X*
ASNS	*ETFA*	*LAGE3*	*PHGDH*	*TBR1*
ASPM	*ETFB*	*LAMA2*	*PI4KA*	*TBX1*
ATP6V0A2	*ETFDH*	*LAMB1*	*PIK3CA*	*TCOF1*
ATP6V1A	*EZH2*	*LAMC3*	*PIK3R2*	*TCTN1*
ATR	*FAM111A*	*LARGE1*	*POLG*	*TCTN2*
B3GALNT2	*FAT1*	*LRP2*	*POLR1C*	*TK2*
B3GNT6	*FAT4*	*MACF1*	*POMGNT1*	*TMEM216*
BICD2	*FDFT1*	*MAN1A2*	*POMGNT2*	*TMEM5*
BMPER	*FGD1*	*MAP1B*	*POMK*	*TMTC3*
CASK	*FGFR3*	*MAP3K4*	*POMT1*	*TP53RK*
CCBE1	*FH*	*MAST1*	*POMT2*	*TPRKB*
CCDC85C	*FIG4*	*MBOAT7*	*PRICKLE1*	*TRAIP*
CCDC88A	*FKRP*	*MECP2*	*PRKDC*	*TRMT10C*
CCDC88C	*FKTN*	*MLYCD*	*PTEN*	*TUBA1A*
CCND2	*FLNA*	*MPDZ*	*PTK2*	*TUBA3E*
CDK5	*FLVCR2*	*MTOR*	*PTPN11*	*TUBA8*
CENPF	*FOXG1*	*MXRA8*	*RAB18*	*TUBB*
CIT	*FTO*	*NANS*	*RAB3GAP1*	*TUBB2A*
COL18A1	*GFM2*	*NDE1*	*RAB3GAP2*	*TUBB2B*
COL3A1	*GNAI2*	*NDEL1*	*RAC1*	*TUBB3*
COL4A1	*GNAQ*	*NDN*	*RAPGEF2*	*TUBG1*
COL4A2	*GNB1*	*NEDD4L*	*RELN*	*TUBGCP6*
CPT2	*GPHN*	*NEK1*	*RHOA*	*USP18*
CRADD	*GPSM2*	*NF1*	*RMND1*	*VIPAS39*
CRK	*GPX4*	*NFIA*	*RNU4ATAC*	*VLDLR*
CSNK2A1	*GRIN1*	*NHEJ1*	*RP1*	*VPS33B*

CSPP1	*GRIN2A*	*NPHP3*	*RTTN*	*WDR26*
CTNNA1	*GRIN2B*	*NPRL2*	*SCLT1*	*WDR62*
CTNNA2	*HIC1*	*NRAS*	*SCN1B*	*WDR73*
CTU2	*HNRNPK*	*NSDHL*	*SCN3A*	*WDR81*
CUL4B	*HRAS*	*NUDC*	*SF3B4*	*YWHAE*
DAG1	*HSD17B4*	*OCLN*	*SHH*	*ZEB2*
DCHS1	*IBA57*	*OFD1*	*SIN3A*	*ZIC1*
DCLK1	*IER3IP1*	*OSGEP*	*SIX3*	*ZNF335*

* The virtual gene panel used in the study was based on searches in the Human Gene Mutation Database (HGMD, Germany; Professional 2019.1) (1) and in Online Mendelian Inheritance in Man (OMIM) (2). The used search terms in HGMD were: 'agyria', pachygyria', 'lissencephaly', 'cortical malformation' and 'polymicrogyria' and in OMIM: 'agyria', pachygyria', 'lissencephaly', 'cortical malformation' and 'polymicrogyria'. Besides, we also added genes to the panel that have been reviewed previously in the context of cortical malformations (3) and polymicrogyria (4).

Table S8. *The primers used in the variant validation and the reverse transcription PCR of CCDC88A.*

Primer name	*Amplicon length (bp)*	*Primer sequence (5' to 3')*
CCDC88A_D310A_F	*466*	GCAACATACCTCAACTCTTGCC
CCDC88A_D310A_R		CCTGGCCAACATGACGAAAC
CCDC88A_DEL_v1&3_F	*468*	CTCAAGGAACCCGGAAGACC
CCDC88A_DEL_v3_R		GCAGGGCTAGAGGAGTTACA
CCDC88A_D310A_cDNA_F	*607*	CAGTCACCCTGTGGTTCTCC
CCDC88A_D310A_cDNA_R		CCAGTTCCCAGCCAAGATGT
CRISPR-Cas-9 sgRNA		GAGCACGAGTTCCCTCTAGT
CRISPR-Cas-9 PAM		TGG
CCDC88A_qPCR_F	*183*	ACAAAAACCATGTTGGAAGACC
CCDC88A_qPCR_R	*183*	CTGTGCCATTTCCAAAGTCAT
GAPDH_F	*100*	ACCCACTCCTCCACCTTTGAC
GAPDH_R *	*100*	TGTTGCTGTAGCCAAATTCGTT

* Primers designed by (5).

References

1. Helbling-leclerc, A., Zhang, X., Cruaud, C., Tesson, F., Weissenbach, J., Tome, F.M.S., Fardeau, M., Tryggvason, K., Guicheney, P. and Inserm, U. (1995) Mutations in the laminin α2–chain gene (LAMA2) cause merosin–deficient congenital muscular dystrophy. *Nature Publishing Group*, **11**, 216–218.
2. Stenson, P.D., Ball, E. V., Mort, M., Phillips, A.D., Shiel, J.A., Thomas, N.S.T., Abeysinghe, S., Krawczak, M. and Cooper, D.N. (2003) Human Gene Mutation Database (HGMD®): 2003 Update. *Human Mutation*, **21**, 577–581.
3. Amberger, J.S., Bocchini, C.A., Schiettecatte, F., Scott, A.F. and Hamosh, A. (2015) OMIM.org: Online Mendelian Inheritance in Man (OMIM®), an Online catalog of human genes and genetic disorders. *Nucleic Acids Research*, **43**, D789–D798.
4. Romero, D.M., Bahi-Buisson, N. and Francis, F. (2018) Genetics and mechanisms leading to human cortical malformations. *Semin Cell Dev Biol*, **76**, 33–75.
5. Stutterd, C.A., Dobyns, W.B., Jansen, A., Mirzaa, G. and Leventer, R.J. (2018) Polymicrogyria Overview. *Polymicrogyria Overview*; University of Washington, Seattle, (2018) .
6. Sikand, K., Singh, J., Ebron, J.S. and Shukla, G.C. (2012) Housekeeping Gene Selection Advisory: Glyceraldehyde-3-Phosphate Dehydrogenase (GAPDH) and β-Actin Are Targets of miR-644a. *PLoS One*, **7**.
